# Large-scale fungal strain sequencing unravels the molecular diversity in mating loci maintained by long-term balancing selection

**DOI:** 10.1371/journal.pgen.1010097

**Published:** 2022-03-31

**Authors:** David Peris, Dabao Sun Lu, Vilde Bruhn Kinneberg, Ine-Susanne Methlie, Malin Stapnes Dahl, Timothy Y. James, Håvard Kauserud, Inger Skrede

**Affiliations:** 1 Section for Genetics and Evolutionary Biology, Department of Biosciences, University of Oslo, Oslo, Norway; 2 Department of Health, Valencian International University (VIU), Valencia, Spain; 3 Department of Ecology and Evolutionary Biology, University of Michigan, Ann Arbor, Michigan, United States of America; University College Dublin, IRELAND

## Abstract

Balancing selection, an evolutionary force that retains genetic diversity, has been detected in multiple genes and organisms, such as the sexual mating loci in fungi. However, to quantify the strength of balancing selection and define the mating-related genes require a large number of strains. In tetrapolar basidiomycete fungi, sexual type is determined by two unlinked loci, *MATA* and *MATB*. Genes in both loci define mating type identity, control successful mating and completion of the life cycle. These loci are usually highly diverse. Previous studies have speculated, based on culture crosses, that species of the non-model genus *Trichaptum* (Hymenochaetales, Basidiomycota) possess a tetrapolar mating system, with multiple alleles. Here, we sequenced a hundred and eighty strains of three *Trichaptum* species. We characterized the chromosomal location of *MATA* and *MATB*, the molecular structure of *MAT* regions and their allelic richness. The sequencing effort was sufficient to molecularly characterize multiple *MAT* alleles segregating before the speciation event of *Trichaptum* species. Analyses suggested that long-term balancing selection has generated trans-species polymorphisms. Mating sequences were classified in different allelic classes based on an amino acid identity (AAI) threshold supported by phylogenetics. 17,550 mating types were predicted based on the allelic classes. *In vitro* crosses allowed us to support the degree of allelic divergence needed for successful mating. Even with the high amount of divergence, key amino acids in functional domains are conserved. We conclude that the genetic diversity of mating loci in *Trichaptum* is due to long-term balancing selection, with limited recombination and duplication activity. The large number of sequenced strains highlighted the importance of sequencing multiple individuals from different species to detect the mating-related genes, the mechanisms generating diversity and the evolutionary forces maintaining them.

## Author summary

Fungi have complex mating systems, and basidiomycete fungi can encode thousands of mating types. Individuals with the same mating type cannot mate. This sexual system has evolved to facilitate sexual mating with offspring from different parents, increasing the chances to recombine into advantageous allelic combination and prune deleterious alleles. We explored the genomes of hundred and eighty strains, combined with experimental mating studies of selected strains, from a non-model organism (*Trichaptum*). We characterized the genomic regions controlling sex. The mating ability of the strains confirmed the role of the mating alleles observed in the genomic data. The detailed analyses of many strains allowed us to observe gene duplication and rearrangements within the mating loci, increasing the diversity within these loci. We supported previous suggestions of balancing selection in this region, an evolutionary force that maintains genomic diversity. These results supports that fungal strains are prone to outcross, which might facilitate the adaptation to new conditions.

## Introduction

Balancing selection is an evolutionary force that maintains genetic diversity [1] receiving long-term attention in evolutionary biology [2]. Heterozygote advantage [1], pleiotropy [3], negative frequency-dependent selection [4], rapid temporal fluctuations in climate [5], and segregation distortion balanced by negative selection [6,7] are modes of balancing selection. These different modes of balancing selection leave similar genomic signatures, such as an increased number of polymorphic sites around the region under balancing selection, and sometimes an enrichment of intermediate-frequency alleles around the selected genomic region [1]. When balancing selection has persisted for a long period, coalescent time of alleles may predate speciation events, and polymorphisms can become shared among distinct species, leading to trans-species polymorphisms [8]. Phylogenetic trees for balanced regions are characterized by the presence of long internal branches [9], and clades with a mixture of species caused by trans-species polymorphisms [10]. The development of methods to detect the genomic footprints of balancing selection [11–13] has unraveled, also with a low number of individuals due to sequencing costs, multiple loci under this type of selection. Well-known examples include: the major histocompatibility locus (MHC) in vertebrates [8]; the ABO histo-blood [14]; non-MHC genes, such as *TRIM5* and *ZC3HAV1* in humans [15,16]; self-incompatibility (SI) loci in plants [17,18] and self/nonself-recognition during vegetative growth in fungi [19]; multilocus metabolic gene networks, such as the *GAL* network in *Saccharomyces* [20,21]; and sexual mating loci in fungi [22].

In basidiomycete fungi, there are numerous examples of balancing selection acting on loci regulating the sexual cycle [22–26]. In this phylum, the sexual cycle involves fusion (plasmogamy) of two genetically distinct monokaryotic hyphae (n or one set of chromosomes), generating a dikaryotic (n+n) hyphae [27–29]. The dikaryon is considered a more stable and long-lived state than the monokaryotic phase, but there are controversies about this assumption due to limited studies [30,31]. Due to this dikaryotic state, plasmogamy is normally separated in time from karyogamy, the fusion of both parental nuclei [32]. In basidiomycetes, karyogamy and meiosis normally occur in specialized structures, the fruit bodies [32]. Mating between two monokaryotic hyphae is determined by one or two sets of multiple allelomorphic genes in the mating (*MAT*) loci. Two different mating systems have evolved among basidiomycetes, referred to as bipolar or tetrapolar mating systems [33]. Mating-type identity in some basidiomycetes, such as *Cryptococcus neoformans*, and members of the sister phylum Ascomycota i.e. *Saccharomyces cerevisiae*, is governed by a single *MAT* locus [34]. This case corresponds to the bipolar system, resembling the sexual system (male or female) in metazoans [35]. However, the ancestor of basidiomycetes developed an evolutionary innovation, the tetrapolar mating system, where two *MAT* loci regulate mating [36]. This new system hinders inbreeding more effectively, since only 25% of the spores from the same individual can mate, compared to 50% for the bipolar species [37]. At the same time, having multiple mating alleles in each *MAT* locus enables extremely effective outcrossing, where most monokaryotic spores or mycelia (derived from different individuals) can establish a dikaryotic mycelium when a compatible mating type partner is found [38].

In strict tetrapolar organisms, the *MATA* locus (syn. *b* or *HD*) contains a series of linked pairs of homeodomain-type transcription factor genes (*HD1-HD2*, syn. *bW-bE*), whereas the *MATB* locus (syn. *a* or *P/R*) is composed of tightly linked G-pheromone receptor genes (*STE3*, syn. *Rcb*, *pra*) and pheromone precursor genes (*Phe3*, syn. *Ph*, *mfa*) [23,39–46]. Nucleotide differences in mating-related genes, without sufficient amino acid changes in key functional domains, belong to the same allelic class [22]. Allelic classes for those genes in *MATA* and *MATB* configure the *MATA* and *MATB* type. The combination of *MATA* and *MATB* types defines mating type identity [34], which controls successful mating and completion of the life cycle [32]. When two monokaryotic (haploid) hyphae of compatible (distinct) *MATA* and *MATB* types conjugate, a structure involved in transferring one of the nuclei during cell division can be observed, called clamp connection, indicating a successful mating [47]. Proteins encoded by *MATA* genes initiate the pairing of the two parental nuclei within dikaryons, they promote clamp development, synchronize nuclear division and septum formation. Proteins encoded by *MATB* genes coordinate the completion of clamp fusion with the subapical cell after synchronized nuclear division and the release of the nucleus, which was initially trapped within the unfused clamp cell [48,49]. Once monokaryons have fused, the *MATB* proteins facilitate septum dissolution and nuclear migration [39]. Experimental crossings in various basidiomycetes, such as *Coprinopsis* and *Schizophyllum*, have been used to infer the number of *MATA* and *MATB* alleles, and results suggest that 12,800–57,600 mating types may exist [50].

However, the molecular confirmation and the knowledge of the diversity of such genomic regions are far behind, as multiple strains must be sequenced. One of the reasons to this delay, is the high nucleotide divergence among *MAT* alleles, which has complicated the study of molecular evolution of the fungal mating systems, where e.g. primer design has been a challenge. Moreover, until now, only a limited number of strains from different species have been analyzed, mainly due to sequencing costs, limiting the quantification of the strength of balancing selection, the presence of trans-species polymorphisms and the detection of mating and non-mating related genes. Due to limited availability of sequenced strains, how each gene within mating loci are involved in mating is unknown.

**Fig 1:**
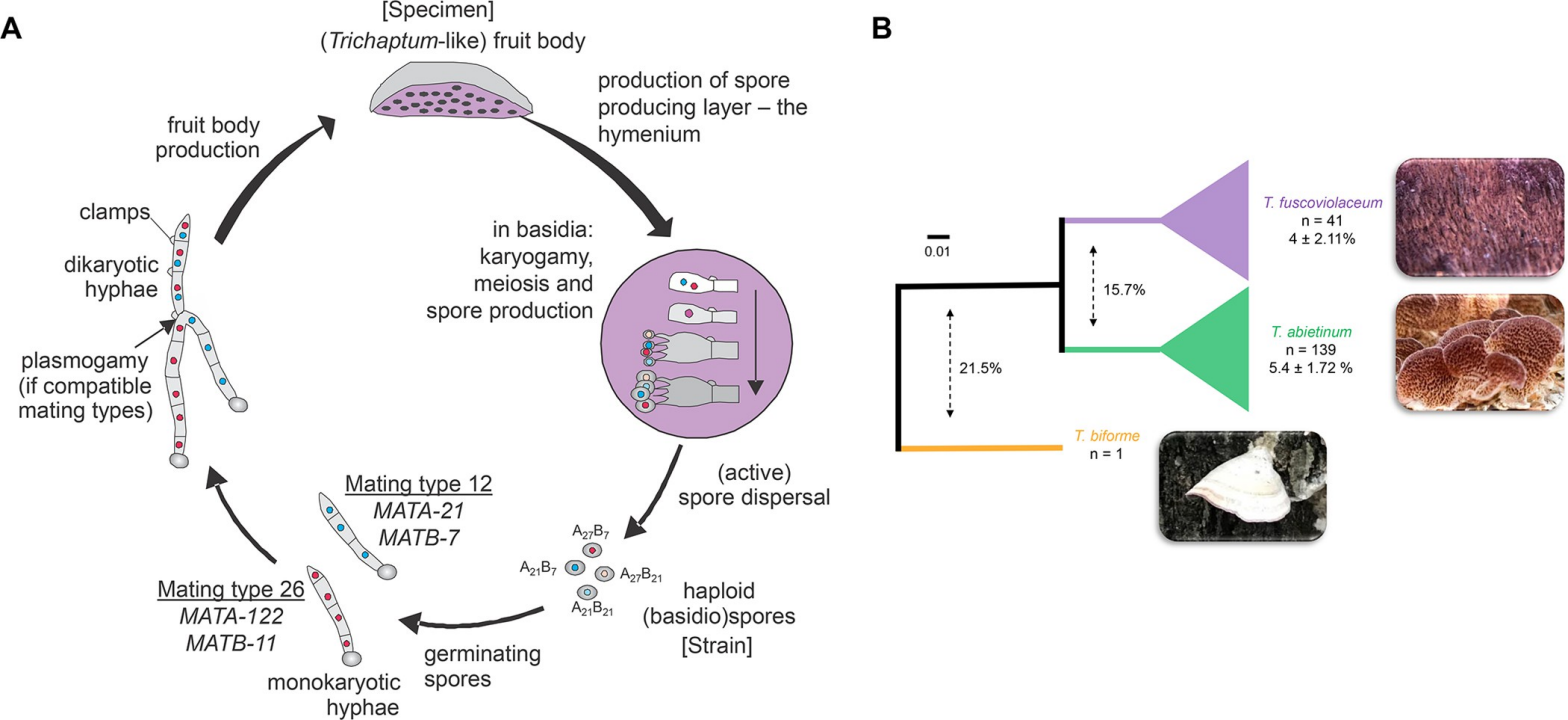
*Trichaptum abietinum* and *T. fuscoviolaceum* are sister-species. A) Schematic representation of the *Trichaptum* life cycle. As an example, *MATA* and *MATB* types, generating two compatible mating types are indicated. A specimen was the original dikaryotic sample, i.e. TA[Number], isolated from the wild environment and stored in a national museum or in our laboratory. Strains were isolated from fruiting bodies and due to their monokaryotic character, we added an M[Number] to the specimen name, TA[Number]M[Number]. Strains are stored in our personal collection at -80°C. B) Schematic Neighbor-Joining (NJ) phylogenetic tree reconstructed using (100 –ANI)/100 values. ANI values go from 100% (identical genomes) to 0% (distinct genomes). In a format (100 –ANI)/100, these values represent divergence. Full NJ and ASTRAL phylogenetic trees can be found in S1 Fig and in iTOL: https://itol.embl.de/shared/Peris_D. The number of strains (n) and the average (100 –ANI)/100 within species are indicated for each species clade. The L15831 genome is included increasing the *T*. *abietinum* collection to 139 strains. Dashed arrows indicate the average (100 –ANI)/100 of pairwise strain comparisons for the compared species. Colors highlight the species designation after the whole genome sequencing analysis.

The type of the mating system in two non-model *Trichaptum* sister species, *Trichaptum abietinum* and *Trichaptum fuscoviolaceum* (Hymenochaetales, Basidiomycota), have been tested in the past, likely because their fruit bodies readily produce monokaryotic spores that germinates and grows *in vitro*, making it easy to conduct crossing experiments in the lab [51]. *Trichaptum abietinum* and *T*. *fuscoviolaceum* are wood-decay fungi with circumboreal distributions [52]. Although, we know their life cycle (Fig 1A), details about how long these organisms spend in monokaryotic or dikaryotic states are still unknown. Previous mating studies have suggested a tetrapolar mating system for *Trichaptum* with an inferred number of 385 *MATA* and 140 *MATB* alleles in *T*. *abietinum* [53]. The mating studies have also revealed that three intersterility groups (ISGs) occur in *T*. *abietinum* [50–54]. However, so far we have no information about the underlying genomic architecture and molecular divergence of *Trichaptum* mating genes.

Here, we study the molecular evolution of the *MAT* genes in tetrapolar basidiomycetes, using a non-model organism. We first sequenced the full genome of a large set of new established monokaryotic cultures from sporulating fruit bodies, collected at different circumboreal locations. Then, we applied bioinformatics and *in vitro* crosses to: i) unravel the genomic location and the structure of the mating-related genes; ii) assess the allelic richness of *MAT* genes; iii) the divergence needed among the alleles in order for the fungi to recognize different mating types, then test whether the genotypic information mirrors phenotypic outcomes of *in vitro* sexual mating; iv) and reveal molecular signals of balancing selection.

## Results

### *Mating regions are highly dynamic in* Trichaptum *species*

To locate the chromosomal position of *MATA* and *MATB* and the genes delimiting the mating regions, we explored different genome assemblers using PacBio long reads and selected the best assembly (Table 1; canu) for one *T*. *abietinum* and one *T*. *fuscoviolaceum* strains. These two species genomes differed with an average 15.7% in a converted ANI (average nucleotide identity) value to divergence value (Fig 1B).

**Table 1:** PacBio assembly stats.

				Before ultrascaffolding			After correction & ultrascaffolding		
**Strain name**	**Descended from**	**Species**	**Assembler**	**Contigs**	**N50 (Kb)**	**L50**	**Scaffolds**	**N50 (Kb)**	**Bases (Mb)**
TA10106M1	TA-1010-6	*T*. *abietinum*	Canu	26	4,268.52	5	12	4,354.20	49.43
TF100210M3	TF-1002-10	*T*. *fuscoviolaceum*	Canu	118	2,011.66	10	12	5,547.79	59.09

**Fig 2:**
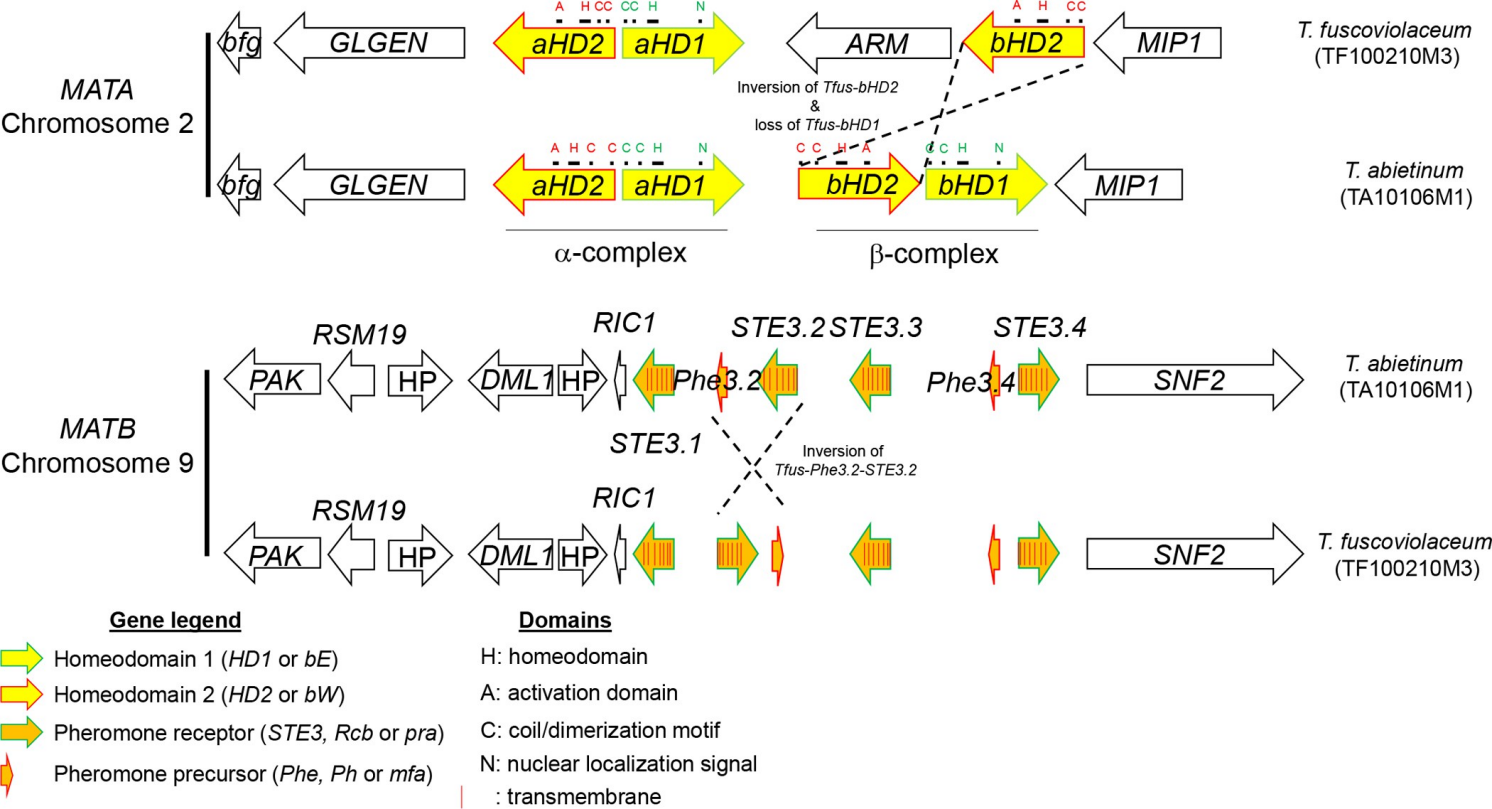
Two homeodomain complexes in *MATA* and four putative pheromone receptors in *MATB* were detected in *T*. *abietinum* and *T*. *fuscoviolaceum*. Schematic representation of the gene composition and direction in both reference genomes. Homeodomain, other functional domains, pheromone precursors and pheromone receptors genes are represented as indicated in the legend. The rest of the genes were colored in black, and the gene names were indicated inside the arrows. *aHD*: alpha-complex homeodomain*; ARM*: ARM-repeated containing protein; *bfg* beta-flanking gene; *bHD*: beta-complex homeodomain; *DML1*: mtDNA inheritance protein; *GLGEN*: glycogenin-1; HP: hypothetical protein; *MIP1*: mtDNA intermediate peptidase; *PAK*: serine/threonine protein kinase; *RSM19*: 37S ribosomal protein S19*; RIC1*: RIC1-domain containing protein*; SNF2*: Snf2 family dna-dependent ATPase; *STE3*: GPCR fungal pheromone mating factor. The Fig is not drawn to scale to facilitate visualization.

Both species potentially contained twelve chromosomes. The genome size of *T*. *abietinum* and *T*. *fuscoviolaceum* was 49 Mbp and 59 Mbp, respectively. Both genomes were highly syntenic with a few small inversions (S2 Fig). The *MATA* and *MATB* loci were located on chromosomes 2 and 9, respectively. *MATA* homeodomain genes were flanked by *bfg*, *GLGEN* on one end and *MIP1* coding sequences on the other (Fig 2). The *MATA* region, defined from *bfg* to *MIP1*, was 17.9 and 19.6 Kbp long in *T*. *abietinum* and *T*. *fuscoviolaceum*, respectively. Both reference genomes contained two homeodomain complexes: alpha- (*aHD*) and beta-complexes (*bHD*). In the reference *T*. *fuscoviolaceum MATA* region, one homeodomain pair, the *bHD1*, was lost, *bHD2* was inverted, and between the alpha and beta-complexes there was a gene encoding an *ARM*-repeat containing protein (Fig 2). *MATB* pheromone receptor and pheromone precursor genes were flanked by *PAK*, *RSM19*, *DML1*, *RIC1* and *SNF2* genes. All these genes together were defined as the *MATB* region, which was 30.3 Kbp long in both species. Four putative pheromone receptors and two pheromone precursor genes were annotated. The *MATB* region was syntenic between both species, except an inverted block containing *STE3*.*2* and *Phe3*.*2* genes in the *T*. *fuscoviolaceum* reference (Fig 2).

### MAT *genes displayed multiple alleles*

**Fig 3:**
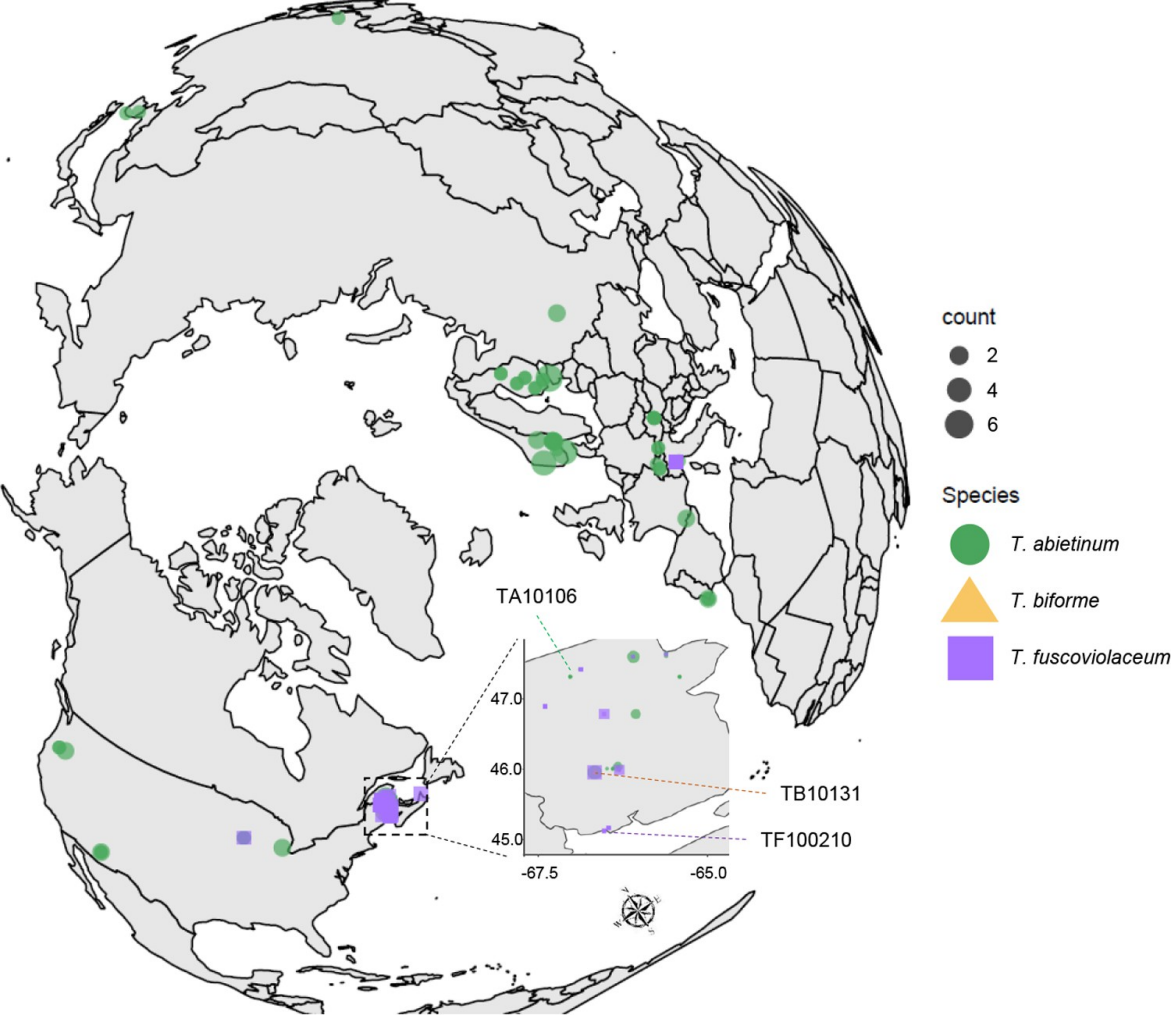
Circumboreal distribution of *Trichaptum* specimens. Geographic distribution of collected *Trichaptum* specimens. 77 European, 98 North American and 4 Asian *Trichaptum* specimens were collected in this study. Specimens were collected mainly from four plant hosts from the genus *Abies*, *Larix*, *Picea* and *Pinus* (Tables 2 and S1). Map was created using R and ggplot2.

The annotated mating genes in the reference genomes were used to search for those genes in the 178 Illumina sequenced strains, collected at circumboreal regions (Fig 3) and a *T*. *abietinum* assembly downloaded from JGI (S2 Table). *Trichaptum abietinum* was the most diverse species based on this collected dataset (average converted ANI 5.4%) (Fig 1B). *MATA* genes were assembled in one contig for 75 *T*. *abietinum*, 25 *T*. *fuscoviolaceum* and 1 *T*. *biforme*. In the case of *MATB*, genes in that region were found in one contig for 116 *T*. *abietinum*, 27 *T*. *fuscoviolaceum* and 1 *T*. *biforme*. For these strains, the mating genes have potentially the same chromosomal location than in reference strains. For the rest of the sequenced strains, the mating genes were found in multiple contigs due to assembly limitations using short reads. Most of those fragmented mating regions might be organized similar to reference strains; however, we observed unexpected coding sequences for 6 strains in the *MATA* region and 2 strains in the *MATB* region, which could suggest that these regions have split and were translocated to different chromosomes or positioned in a new chromosomal location (S3 Table).

**Fig 4:**
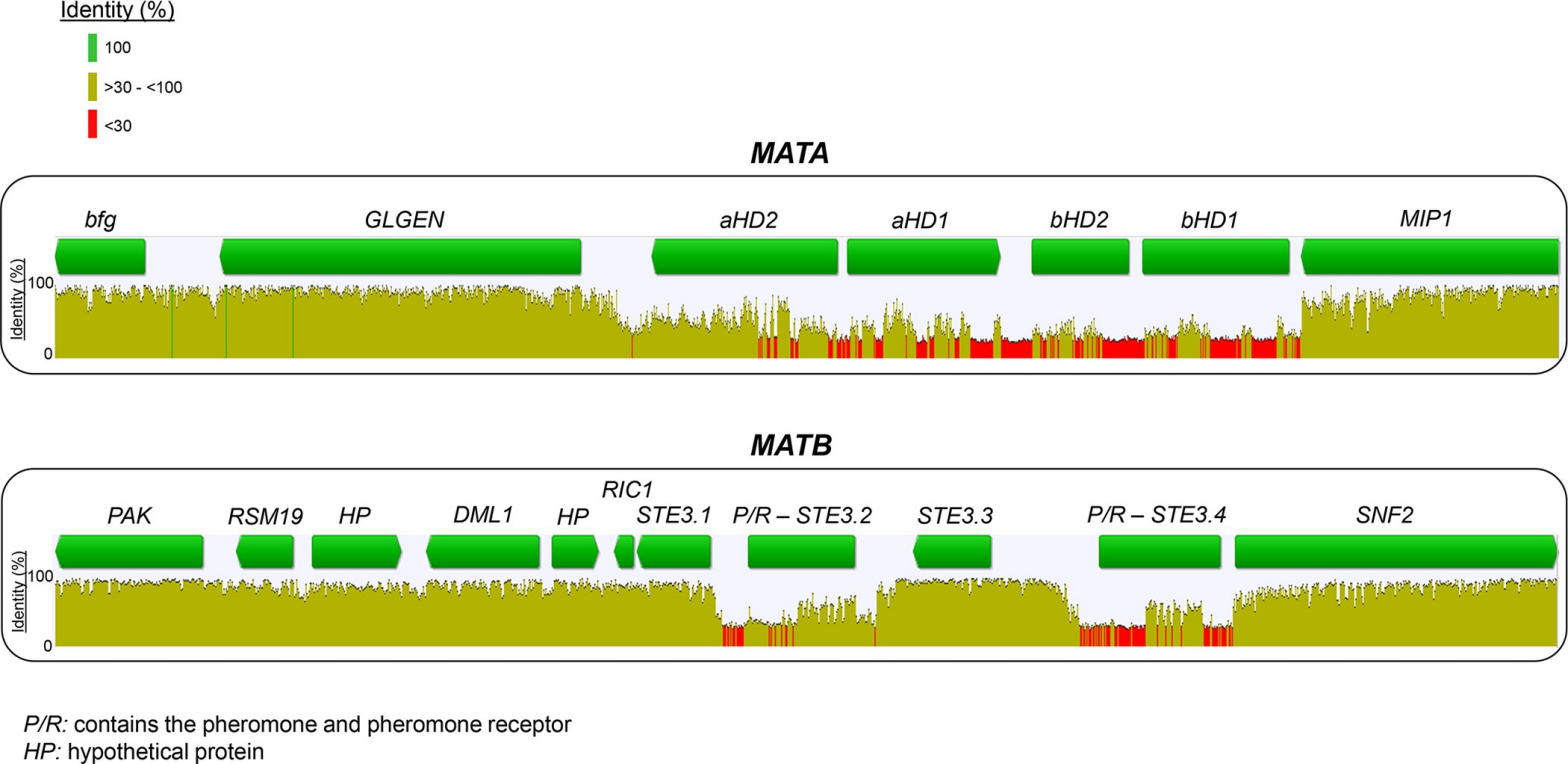
High nucleotide diversity among mating genes. Identity values of nucleotide alignments for *MATA* and *MATB* regions are displayed. Gene arrows indicate the coding direction; however, when gene direction differed among strains (Fig 5), we represented a green rectangle. Bar colors represented the level of identity according to the legend. Geneious’ identity values were calculated based on each nucleotide position and represent the percentage (y-axis) of sequences with an identical nucleotide compared to the consensus sequence. The *MATA* alignment includes 175 isolates (3 species), excluding those isolates found to split the region in potentially different chromosomal locations (S3 Table). The *MATB* alignment includes 179 isolates (3 species), excluding those isolates found to split the region in potentially different chromosomal locations (S3 Table).

**Fig 5:**
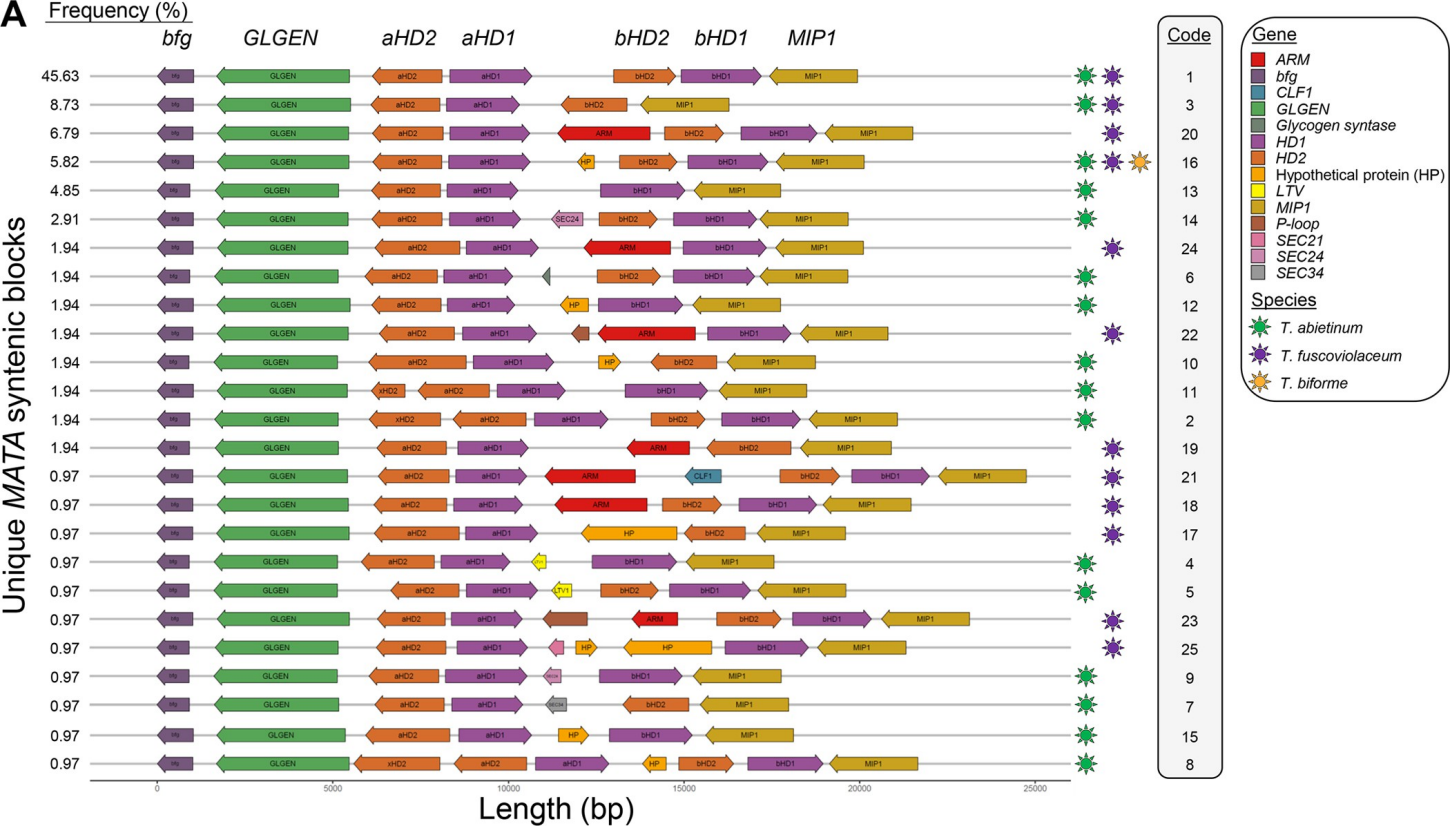
Mating A region is highly dynamic and show multiple rearrangements among *Trichaptum* strains. *MATA* gene order representations for *Trichaptum* strains with *MATA* genes assembled in one contig. The percentage of strains containing a specific *MAT* block order is indicated in the left. Genes were colored according to the legend on the right. Species containing a particular *MAT* block are represented by colored stars at the right of the *MAT* block and were colored according to the legend. Coding sequence direction is represented by the arrows. Code numbers link the strains in S2 Table with the displayed mating structure.

**Fig 6:**
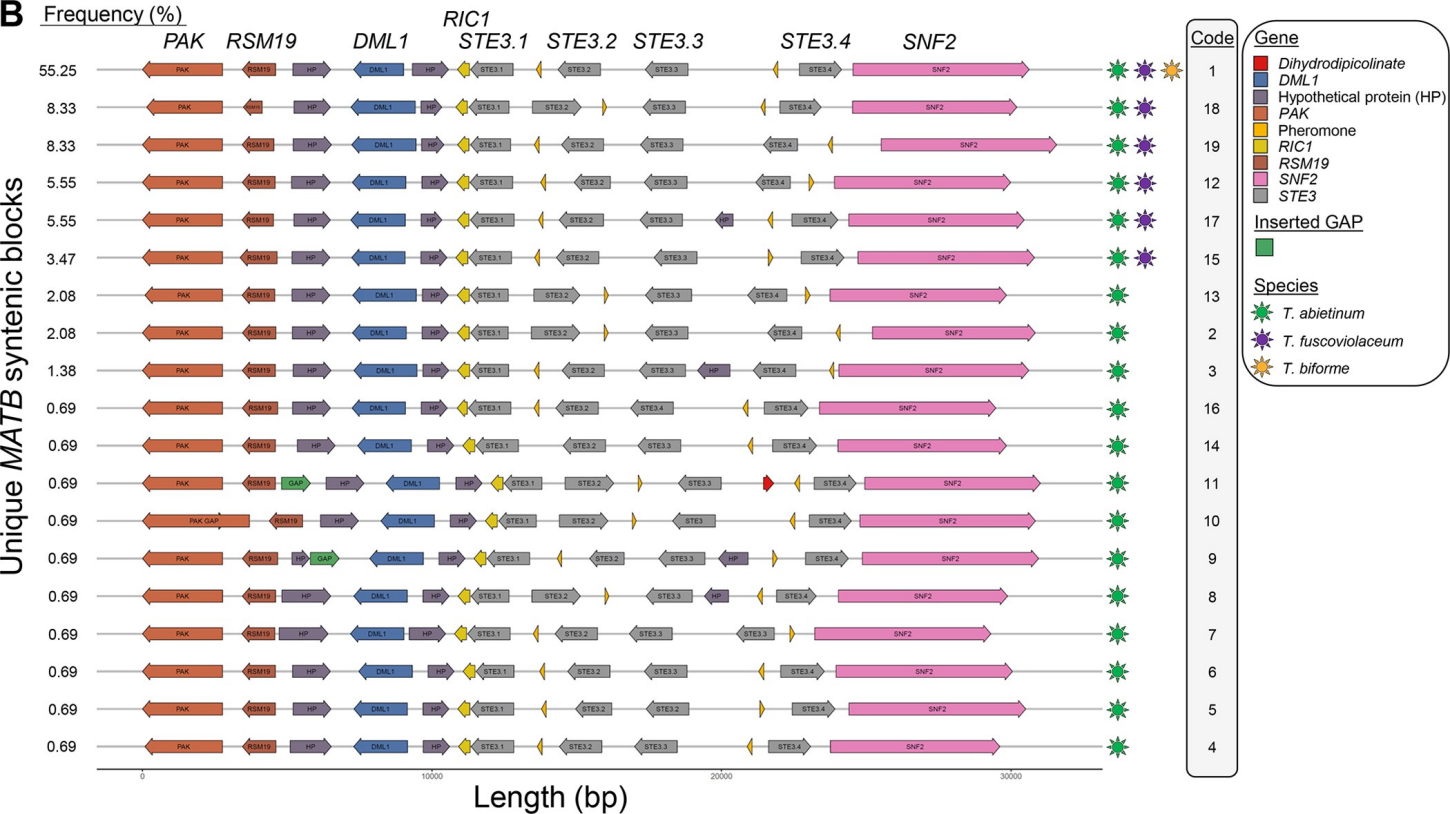
Mating B region is highly dynamic and show multiple rearrangements among *Trichaptum* strains. *MATB* gene order representations for *Trichaptum* strains with *MATB* genes assembled in one contig. We showed the *MATB* region for those strains where the assembly was contiguous from *RIC1* to *SNF2*. In some strains, the region from *RIC1* to *PAK* was contained in multiple contigs. Those contigs were joined (ultrascaffolding) and ordered according to the reference genomes (see Material and Methods section). To ultrascaffold, we inserted 999 Ns between joined contigs, annotated as a GAP in the legend. For that reason, GAP label is drawn. The percentage of strains containing a specific *MAT* block order is indicated in the left. Genes were colored according to the legend on the right. Species containing a particular *MAT* block are represented by colored stars at the right of the *MAT* block and were colored according to the legend. Coding sequence direction is represented by the arrows. Code numbers link the strains in S2 Table with the displayed mating structure.

An initial analysis of nucleotide conservation of the mating regions indicated that flanking genes were conserved, as well as *STE3*.*1* and *STE3*.*3*. However, the rest of putative mating genes were highly diverse (Fig 4). Gene order comparison among strains highlighted that the most common *MATA* and *MATB* syntenic blocks were both present in *T*. *abietinum* and *T*. *fuscoviolaceum*, and the frequent *MATB* syntenic block was present in the three species (Figs 5 and 6). *Trichaptum biforme* and five other *Trichaptum* strains, differentiated from the most frequent *MATA* configuration by the presence of a hypothetical protein (Fig 5). All this suggest that the most frequent *MATA* and *MATB* gene configurations, represented for the reference *T*. *abietinum* strain (Fig 2), were present in the ancestor of these three *Trichaptum* species. The gene order of *HDs* in the alpha-complex was conserved among all *Trichaptum* strains. However, frequent inversions of the *bHD2* gene and absence of one of the two *bHD* genes were detected. An interesting observation was the presence of an additional *HD2* gene (*xHD2*) upstream the alpha-complex in six *T*. *abietinum* strains (Fig 5). The coding sequence of *xHD2* was truncated, indicating an ongoing process of pseudogenization. In the *MATB* region, all strains contained two pheromone precursor genes, one located between *STE3*.*1* and *STE3*.*2*, and a second between *STE3*.*3* and *STE3*.*4*. The orientation of *STE3*.*2*, *STE3*.*4* and pheromone precursor genes varied among strains (Fig 6).

We were able to infer several domains and motifs in mating genes. *HD1* and *HD2* homeodomain genes contained three and four exons, respectively, whereas *STE3* genes, characterized by the presence of seven transmembrane domains, included 4 to 6 exons. Homeodomain genes were characterized by the presence of the typical homeobox domain (Fig 2). In each homeodomain protein alignment, we found conserved amino acid sequences in potentially functional homeodomains (S3 Fig), likely because they are essential for the activation of the expression of target genes. The nuclear localization signal was detected in HD1 proteins, with the presence of bipartite sequences (S3 Fig). Regions enriched in prolines are indicative of putative activation domains (AD), which were conserved in HD2 proteins (S3 Fig). It is important to note an additional conserved region at the C-terminal of HD1 proteins (S3 Fig). Coiled coils related with heterodimerization were likely located at the N-terminal (Fig 2).

Using the pheromone_seeker.pl script, we were able to detect most of the pheromone precursor genes. Basically, the script searches the prenylation signal, which is important to transport the pheromone precursor peptide to the plasma membrane, where cleavage occurs in the maturation site of the precursor. Maturation will release the active pheromone, consisting of the residue from near the maturation site (E) to the C in the CaaX motif (S4 Fig). Maturation approximately generates a peptide of 10–11 amino acids [55]. However, some pheromone precursors were not detected due to unexpected amino acids in the CaaX motif (S4 Fig). We found multiple examples in both pheromone precursors (Phe3.2 and Phe3.4), where the canonical CaaX motif contained a polar (p) amino acid (threonine, T), displaying an uncommon CpaX motif. Most of the pheromones contained an aspartic amino acid following the starting methionine. The presence of both aspartic and glutamic amino acids in the maturation site was highly conserved in *Trichaptum* pheromones.

Despite the dynamic nature of both mating regions (Fig 5), where rearrangements and gene losses were frequent, and the observed high nucleotide diversity (Fig 4), the results are highlighting the effects of natural selection retaining important residues located in domains proven to be linked to the activity of mating proteins.

### Distinct mating types generate compatible mating crosses within species

**Fig 7:**
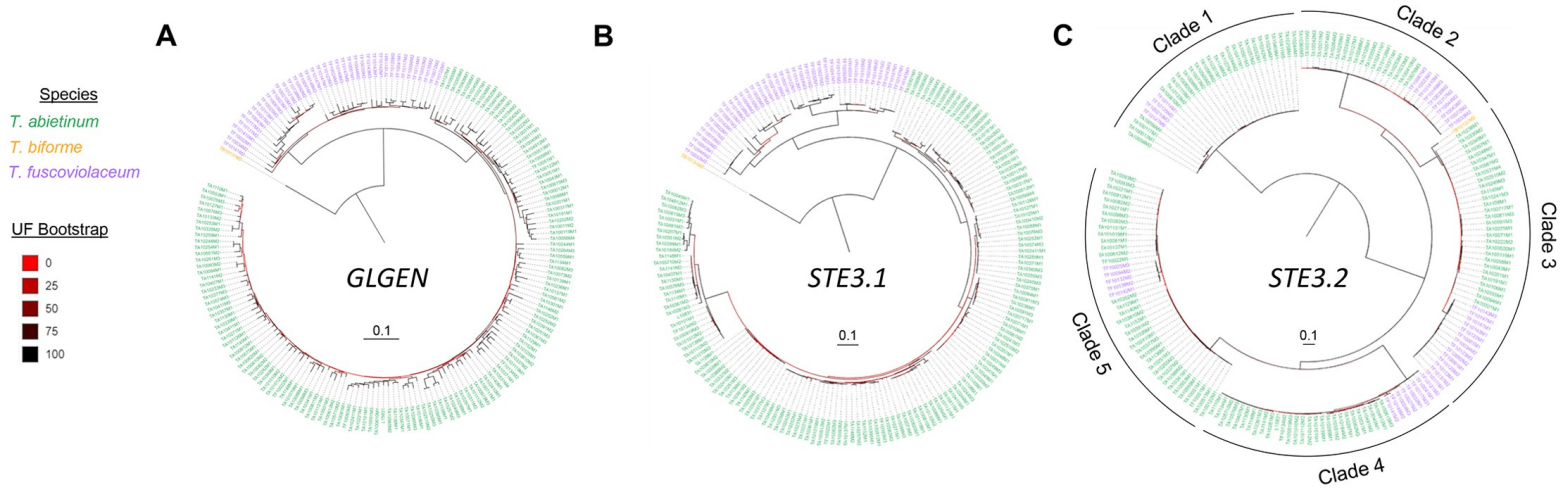
ML phylogenetic tree topology of mating proteins suggests balancing selection and trans-species polymorphisms. ML phylogenetic protein trees of *GLGEN* (a flanking gene), *STE3*.*1* (a potential non-mating pheromone receptor protein) and *STE3*.*2* (a mating pheromone receptor protein) are represented in panel A, B and C, respectively. Strains were colored according to the species designations as indicated in the legend. Branch support was assessed using the ultrafast bootstrap (UF bootstrap) method. UF bootstrap is indicated in each branch by a gradient color according to the legend. Scale bar is represented in number of amino acid substitutions per site. The rest of phylogenetic protein trees and more detailed trees for the represented here are found in S5 Fig.

Mating gene combinations define mating types. To predict mating types, we first quantified the number of clades in reconstructed phylogenetic trees (Figs 7 and S5). The number of clades in the phylogenetic trees varied from 5 to 28. Each clade was considered as a different allelic class. Sequences in the same allelic class encoded for proteins with an AAI higher than 86% (S6 Fig). The highest number of allelic classes was found among alpha-complex homeodomain genes where we detected evidence of recombination (S4 Table).

A combination of allelic classes for homeodomain gene pairs (*HD1* and *HD2*) in the alpha-complex and in the beta-complex defines the *MATA* type (S3 Table). In total, we predicted 207 *MATA* (23 alpha x 9 beta) and 189 *MATA* (21 alpha x 9 beta) types for *T*. *abietinum* and *T*. *fuscoviolaceum*, respectively. A combination of allelic classes for pheromone receptors genes *STE3*.*2* and *STE3*.*4* defined the *MATB* type (S3 Table). Predictions suggested 65 *MATB* types (5 *STE3*.*2* x 13 *STE3*.*4*) for both species. Note that *STE3*.*1* and *STE3*.*3* were not considered to be defining *MATB* types because as we describe in the next section, they were not predicted to be mating-related genes. The number of potential mating types predicted by combining *MATA* and *MATB* types is at least 13,455 (207 *MATA* x 65 *MATB*) mating types in *Trichaptum abietinum* and 12,285 (189 *MATA* x 65 *MATB*) mating types in *T*. *fuscoviolaceum*. Once we defined the mating types of strain samples, we calculated the AAI by pairwise comparisons of protein sequences of strains containing the same mating type. We detected high conservation within species for all proteins (AAI = 100%), and higher conservation of pheromone receptors between species (AAI > 95–98%) than for homeodomain genes (AAI > 78–83%), suggesting pheromone receptors were more constrained to accumulate non-synonymous mutations compared to homeodomains (S7 Fig).

These predicted mating types were helpful to set up mating experiments (S3 Table). We tested the outcome of crosses between selected monokaryotic strains from the same species and between species (S5 Table). We assumed a successful mating when clamp connections were formed (S8 Fig). Our expectations, based on the molecular characterization, were confirmed in all within species crosses. Crosses using strains with identical *MATA* types did not generate clamps when *MATB* types were expected to be compatible, and vice versa. These results demonstrate that identical (AAI > 86%) *MAT* allelic classes generate the first mating barrier.

We also included some strains derived from the same dikaryotic specimen (S3 Table), where most of them showed at least a pair of compatible *MATA* and/or *MATB* types. These strains helped us to unfold the original allelic class composition of the parental specimen (S3 Table). Due to the unlinked nature of *MATA* and *MATB* regions and limited number of studied strains from the same specimen, some strains had identical mating types, thus did not reveal the original mating type composition of the parental specimen.

No clamps were observed in crosses between species with compatible mating types suggesting other mechanisms are involved in the generation of pre-zygotic barriers between *Trichaptum* species.

### Long-term balancing selection left footprints in the mating regions

To infer the evolutionary history of these mating genes controlling the sexual cycle, and the flanking genes, and to test whether they agree with the species tree (Figs 1B and S1), we reconstructed Maximum Likelihood (ML) individual protein trees (S5 Fig). For most proteins encoded in flanking genes and for both STE3.1 and STE3.3 proteins, phylogenetic trees clustered strain sequences according to their species designation (Figs 7A, 7B, S5A and S5B, S5H-S5L and S5N). However, phylogenetic protein trees for homeodomains (aHDs and bHDs), two pheromone receptors (STE3.2 and STE3.4), MIP1 and SNF2 disagreed with the species tree (Figs 7C and S5C–S5G, S5M, S5P, and S5O). These trees were characterized by long internal branches and a mixture of species-specific sequences in different clades. All these results pointed to the presence of trans-species polymorphisms likely due to long-term balancing selection.

To further test whether long-term balancing selection is acting on the mating regions, we quantified nucleotide statistics and performed a multilocus HKA test using the mating genes and a collection of universal single-copy orthologs (BUSCO) genes. We first tested the reciprocal monophyletic nature of BUSCO gene collection. As expected from the species tree (S1B Fig), most of annotated BUSCO genes (eighty-three percent) showed reciprocal monophyly for both species, *T*. *abietinum* and *T*. *fuscoviolaceum*, and 98.64% of the rest of genes (174 genes of 1026 BUSCO genes) showed complete monophyly for one of the two species. This BUSCO dataset suggests a clear diversification of both *Trichaptum* species, and supports the utility of this dataset to set the neutral evolution values of the next analyzed nucleotide statistics.

**Fig 8:**
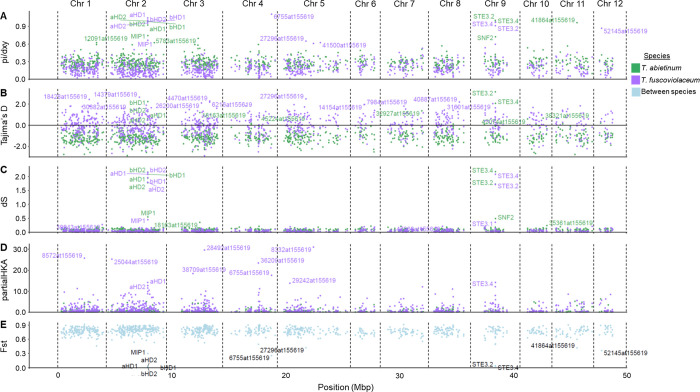
Multiple nucleotide statistics support long-term balancing selection in genes located in the mating region. Ratio of nucleotide diversity (Pi) and absolute divergence (dxy), Tajima’s D, average number of synonymous substitutions per synonymous sites (dS), and relative divergence (Fst) values for each single-copy orthologous and mating genes are reported in panels A), B), C) and E), respectively. Gene contribution to the significance of a HKA test (partial HKA) are represented in panel D). Gene names containing 1% of the highest values (panels A, B, C, and D) or 1% of the lowest values (panels E and F) are displayed. *T*. *fuscoviolaceum* gene names with the highest partial HKA values are displayed due to the significant result of the HKA test (*p*-value = 3.13 x 10^−39^). Each dot represents a gene and we used the annotation in *T*. *abietinum* to represent the position of each gene. Annotation file can be found in the Github page dedicated to this project. Dots were colored according to within species calculations (green or purple for *T*. *abietinum* and *T*. *fuscoviolaceum*, respectively) or between species comparison (cyan). Chr: chromosome. These analyses include all strains from both species.

We observed an elevated number of the average number of synonymous substitutions per synonymous sites (median dS > 1.71) and non-synonymous substitutions per non-synonymous sites (median dN > 0.22) for the mating genes compared to the flanking and BUSCO genes (Figs 8C and S9, median dS < 0.55, median dN < 0.10). dS and dN values in mating genes were more than 20x and 3x higher than values for BUSCO genes, respectively (S6 Table). This result was an additional support that balancing selection acts on the mating regions. Moreover, similar levels of dS and dN (S9 Fig, ratio comparison of 0.95–1.03) were observed within and between species in pairwise comparisons of mating genes, indicating that these polymorphisms were not species-specific and recent introgressions were not involved in the generation of trans-species polymorphisms. This was coherent with a scenario where alleles segregated before the diversification of the species. It is important to note that dS and dN values for two putative receptors, *STE3*.*1* and *STE3*.*3*, differed from the other mating genes and that they displayed similar low values as most flanking and BUSCO genes (S9 Fig). In addition, for these two putative non-mating pheromone receptor genes, the dS and dN values were 1.41–3.17 times higher between than within species pairwise comparisons, as we would expect if most of the mutations accumulated after the speciation of *T*. *abietinum* and *T*. *fuscoviolaceum*. *MIP1* and *SNF2* dS values were slightly more elevated than BUSCO genes (S6 Table), but values from between species comparisons were more elevated than within pairwise comparisons (S9 Fig). This indicated that the elevated dS values, compared with BUSCO genes, are caused by linkage disequilibrium, where the effects of balancing selection in the closest mating gene were not completely broken by recombination.

To infer whether other nucleotide statistics supported balancing selection, we explored gene values deviating from the rest of the genome (Fig 8). Homeodomain (*HD1s* and *HD2s*) and pheromone receptor genes (*STE3*.*2* and *STE3*.*4*) deviated from the distribution of 99% of values in at least four nucleotide statistics (elevated pi/dxy ratio, high dS values, low Fst and high Tajima’s D), all in agreement with a balancing selection scenario maintaining trans-species polymorphisms for multiple alleles (Fig 8). Five BUSCO genes were detected in at least two statistics, deviating from the rest of the genome (Fig 8). Those five genes were also detected to show a phylogenetic topology incongruent with a complete reciprocal monophyly, except 18163at155619 where only *T*. *fuscoviolaceum* sequences were monophyletic (S10 Fig). The detected genes encoded for an acetolactate synthase (27296at155619), a ribosomal protein L38e (52145at155619), a non-specific serine/threonine protein kinase (6755at155619), a protein kinase-domain-containing protein (18163at155619) and a NF-kappa-B inhibitor-like protein 1 (41864at155619).

The geographic distribution of *MATA* and *MATB* alleles did not suggest a bias towards a particular continent (S5 and S11 Figs), supporting an evolutionary scenario of long-term balancing selection for mating genes.

### New mating genes generated by duplications

The diversity of allelic classes might be generated by the accumulation of point mutations or, as stated above for the alpha-complex, by recombination of existing variants. However, we detected a new HD2 (xHD2) gene in some strains (Fig 5A and S3 Table). A ML phylogenetic tree of all HD2 protein sequences (S12 Fig) clustered xHD2 proteins in two allelic classes, aHD2.8 and aHD2.10; however, the closest aHD2 in these strains were from different allelic classes: aHD2.18 and aHD2.24, respectively (S3 Table). The limited presence of *xHD2* genes in other strains and the high similarity of the proteins to two aHD2 proteins points to two recent duplications and transfers to other mating regions.

Phylogenetic analyses of homeodomain proteins with other fungal sequences indicated that the beta-complex HD proteins were much older than Hymenochaetales (S13A Fig), which was in accordance with the lower identity values observed for pairwise comparisons within bHD than within aHD (S6 Fig). Except aHD1.12, the rest of aHD proteins were identified in *Trichaptum* species. A similar result can be observed for pheromone receptors, where most *Trichaptum* pheromone receptor proteins were closely related, except two proteins, encoded in *STE3*.*2* and *STE3*.*4* genes, which were related to pheromone receptor proteins from other fungal species (S13B Fig). It remains to be answered whether the alpha-complex was generated by a duplication from the beta-complex or a more complex scenario generated this additional homeodomain complex in *Trichaptum*.

## Discussion

### Mating genes diversity was maintained by balancing selection

Retaining multiple mating alleles appears to be beneficial as it promotes outcrossing [36]. The multiallelic character of mating types promotes a potential outcross event to occur in 98% of crosses [36,56]. How this mating diversity originated is not clear, but we demonstrated that some levels of recombination and duplications might play a role. Fifteen recombinant variants in the alpha-complex and two recent *aHD2* duplications were detected in *Trichaptum*. It was previously thought that recombination was suppressed or limited in the mating regions [57], and that duplication and diversification events were limited to Agaricales [42]. Recombination is suppressed by the presence of inversions and/or gene losses, which might generate hemizygous strains, observed in mating loci and genomic regions under balancing selection [58]. The rearrangements observed in *Trichaptum* beta-complex brings another layer of complexity to *MATA* region, which is comparable to the complexity previously described for *MATB* genes [36]. Rearrangements in both *MAT* loci might be an important factor suppressing recombination in these genes. On the contrary, the gene order conservation of the alpha-complex does not completely suppress recombination, in accordance with evidence of ongoing recombination between mating genes [59] and their flanking genes in other fungal organisms [60]. Our observations highlight how studying a high number of strains of the same species can unravel previously underestimated mechanisms that generate diversity in mating genes.

We have demonstrated that balancing selection is likely the main force retaining genetic diversity in the mating genes. Evidence of balancing selection has been proposed for homeodomain genes in the pathogenic root decay fungus *Heterobasidion* (Russulales) [26], as well as in pheromone receptors of *Mycrobotryum* species (Mycrobotryales) [24]. The action of balancing selection in *Trichaptum* and in other fungi appears to have occurred before the speciation event, generating multiple cases of trans-species polymorphisms [26]. The genetic signatures of balancing selection highlighted that two pheromone receptors in *Trichaptum* strains are likely non-mating genes, this could only have been unraveled by including multiple strains as we have done here. In Agaricomycotina, it is frequent to detect multiple pheromone receptors, some of them not involved in mating functions [40,42,61]. The role of these non-mating pheromone receptors will deserve further investigation.

It has long been speculated about the action of balancing selection in the *MATA* flanking gene, *MIP1* [25,60]. *MIP1* encodes a mitochondrial intermediate peptidase 1, which is a thiol-dependent metallopeptidase involved in the last step of protein maturation targeted to the mitochondria, where MIP1 cleaves off an octapeptide of immature proteins [62]. The genomic footprints detected in *MIP1* are likely due to the action of linkage disequilibrium, as *MIP1* is close to the beta-complex *HD* genes. It has been speculated that *MIP1* signals of balancing selection and trans-species polymorphisms might be due to a role in mating, such as MIP1 involvement in mitochondrial inheritance, functioning as a suppressor of selfish mtDNA [63]. However, this function is not well-supported. Other genes encoding proteins involved in mitochondrial functions have been found linked to mating genes [60]. In *T*. *abietinum and T*. *fuscoviolaceum*, we found *RSM19*, a 37S ribosomal protein S19, linked to *MATB*. However, we did not detect signals of balancing selection in this gene. In addition, some signals of balancing selection and trans-species polymorphisms were detected in *SNF2*, a gene located in the *MATB* region, encoding a DNA-dependent ATPase protein. The analogous signals of balancing selection between *SNF2* and *MIP1* might support that the balancing selection signal in both genes is due to linkage disequilibrium, and the signal is just a consequence of the action of balancing selection in the neighbor mating genes [60].

### Mating genes and organization resemble other basidiomycetes suggesting similar origin

Sampling and studying the genomes of a wide collection of *Trichaptum* strains have unraveled the dynamic nature of mating gene architectures. With two homeodomain complexes, *Trichaptum MATA* gene organization is similar to other Hymenochaetales, such as *Phellinus lamaoensis*, *Phellinus sulphurascens* (both species from the *Phyrrhoderma* genus) and *Schizopora paradoxa* [64]. The presence of homeodomain complexes with just one pair of homeodomain genes is also observed in Hymenochaetales [64]. In other Hymenochaetales species, such as *F*. *mediterranea* and *Porodaedalea pini*, the location of *GLGEN* gene is more distant and interrupted by multiple *ORFs* [64,65]. Notably, the *Phyrrhoderma* species and *F*. *mediterranea* [64,66] are bipolar, in contrasts to the tetrapolar *Trichaptum* strains. *Trichaptum* and other Hymenochaetales species, such as *Hypodontia* and *S*. *paradoxa*, have conserved the ancestral tetrapolar system of basidiomycetes [36].

According to mating studies, the formation of clamp connections is facilitated by the presence of at least one different allele at one of the multiple *MATA HD* complexes and one at the *MATB* P/R loci. Here, we demonstrated by mating experiments and genomic analyses that protein identity must be lower than 86% to function as different mating type, although important protein domains and motifs are conserved. We inferred that around 270 *MATA* types (30 alpha x 9 beta) and 65 *MATB* types (5 *STE3*.*2* x 13 *STE3*.*4*) are segregating in *Trichaptum* species, which indicates around 17,550 mating types. These numbers are close to the estimated number of alleles, 20,000 mating-types, in a previous study of *T*. *abietinum* [50], suggesting that our sequencing efforts, molecularly characterized most of the *Trichaptum* mating alleles. In other tetrapolar basidiomycete species, such as the model species *Coprinopsis cinerea* and *Schizophyllum commune*, the number of mating types is also similar, around 12,800 (160 *MATA* x 81 *MATB*) and 23,328 (288 *MATA* x 81 *MATB*), respectively [51]. We inferred that beta-complex HD alleles were segregating in other Agaricomycetes, suggesting that these HD proteins are much older than alpha HD, a result that is supported by the ongoing recombination events in the alpha HD. Moreover, we cannot discard that alpha-complex alleles may be exclusively specific of *Trichaptum*. Allele *aHD1*.*12* points to potential alpha-complex alleles segregating in other Hymenochaetales, but just thirteen Hymenochaetales species have been fully sequenced, and usually only one representative of each species, except for the three sequenced *Pyrrhoderma noxium* strains. Thus, there are few available genomes to compare.

A new pheromone precursor motif containing a polar amino acid in CaaX motifs was detected by this large-scale sequencing effort. We are not aware of CpaX motifs in other Basidiomycetes, although this motif was observed in pheromones of some Ascomycetes species [67,68]. The whole genome sequence of other Hymenochaetales and other fungal orders, and the increased number of strains from multiple species, will clarify the evolutionary history of the alpha-complex and protein patterns observed here.

### Trichaptum*—A valuable toolset for studies of genes related with sex*

The dataset contributes with a large number of genome assemblies from two non-model species and a representative for a third species of the *Trichaptum* genus. The existence of at least two North American intersterility groups (ISGs) that are partially compatible with a third European group in *T*. *abietinum* indicates three potential differentiated lineages [52–54]. Even though we did not perform a population genomic analysis in this study, multiple well-differentiated clades can be inferred by using ANI values and BUSCO phylogenetic species trees, supporting some population structure in this strain collection. The presence of ISG in *T*. *fuscoviolaceum* is not previously confirmed based on mating studies [52,54]. However, we hypothesize that there are at least two potential lineages due to the presence of two well-differentiated *T*. *fuscoviolaceum* clades, as suggested by Seierstad *et al*. [52]. ANI dissimilarity values between these lineages were nearly as high as values detected in *T*. *abietinum*, supporting the hypothesis about population structure in *T*. *fuscoviolaceum*. However, the difference in the levels of populations and the presence of clear ISG in one species and not in the other might be the reason of the differences in the distribution of Tajima’s D values, with more BUSCO genes with negative Tajima’s D values in *T*. *abietinum* than in *T*. *fuscoviolaceum*.

The potential number of *Trichaptum* lineages together with these new genome sequences and diversity of mating types, provide an exceptional tool for comparative genomics and functional genomics to study the evolution of sex in fungi and mechanisms involve in the sexual cycle.

## Conclusion

We have demonstrated the importance of sequencing several strains of fungal species to detect mating-related genes, and to unravel the strength and footprints of long-term balancing selection in mating genes. Events previously thought of as uncommon in mating genes, such as recombination and duplications, have been detected in mating-related genes with conserved gene order. The *Trichaptum* dataset highlights how diverse and dynamic the mating loci are. These mating genes play a fundamental role in promoting outcrossing events and have consequently been targets of long-term balancing selection. The action of balancing selection leaves signatures of multiple trans-species polymorphisms beyond the genus level. Comparative genomics and phylogenomics were important tools to locate mating genes and characterize the number of alleles retained by balancing selection. Mating proteins with less than 86% identity generated compatible mating types, as we demonstrated by experimental crosses. Despite the number of alleles and the high diversity among them, important domains and motifs are still conserved due to their critical role during the life cycle. Questions regarding the effects of mutations in the interaction between homedomain proteins or receptors and pheromones, especially the presence of non-aliphatic amino acids in the CaaX motif (i.e. a CapX motif), and which role the linked mating genes, such as *MIP1*, are playing during the life cycle are exciting areas of research. This newly sequenced collection of *T*. *abietinum* and *T*. *fuscoviolaceum* makes a step-forward to re-establish these fungal organisms as a model system in evolutionary research.

## Material and methods

### Trichaptum *collection*

A total of 180 *Trichaptum* strains from the northern hemisphere were included in the study: 138 *T*. *abietinum* (67 European, 67 North American and 4 Asian), 41 *T*. *fuscoviolaceum* (10 European and 31 North American) and one North American *T*. *biforme* (S1 Table). GPS coordinate format conversion was generated with GMScale 0.5.1 to plot the geographic distribution in R, using ggmap 3.0.0, ggplot2, ggrepel 0.8.2, and mapdata 2.3.0. *Trichaptum abietinum* were frequently isolated from *Picea* trees, whereas *T*. *fuscoviolaceum* was frequently associated with *Abies* (Tables 2 and S1).

**Table 2:** Distribution of host trees for *Trichaptum* specimens.

Genus host	*T*. *abietinum*	*T*. *biforme*	*T*. *fuscoviolaceum*
*Abies*	18	1	23
*Larix*	10	0	0
*Picea*	74	0	2
*Pinus*	25	0	10
Unknown	11	0	6

### Monokaryon generation and genomic DNA isolation

To facilitate the study of highly diverse genomic regions, such as the mating loci, and to avoid heterozygosity issues in other genomic regions, we isolated single fungal spores produced by fruit bodies from dikaryon cultures (original isolated specimens, n+n), and formed monokaryotic cultures (haploid strains, n) in the lab. These monokaryotic cultures were made by hydrating dried field collected fruit bodies in the lab, and allowing the fruit bodies to eject spores onto 3% malt extract agar plates with 10 mg/L tetracyclin, 100 mg/L ampicillin, 25 mg/L streptomycin and 1 mg/L benomyl. Four germinated single spores were transferred to four new 3% malt extract agar plates with identical mixture of antibiotics and benomyl, resulting in monokaryotic cultures. All monokaryotic cultures were checked for clamp connections, which supports that only one spore was picked and mating did not already occurred among spores from the same fruit body. One of the four monokaryotic cultures were selected (except for seven specimens were more than one monokaryotic culture were included) for further analyses (S3 Table). Before DNA extraction, monokaryon cultures were grown for 2–3 weeks on nitex nylon (Sefar AG, Heiden, Switzerland) on 3% malt extract agar plates.

Two different DNA extraction protocols were used depending on the sequencing method. For Illumina sequencing, tissue from 1/4^th^ plate was scraped off the nylon and directly homogenized in 2 ml Lysing Matrix E tubes (MP Biomedicals, Santa Ana, CA, USA) on a FastPrep-24 (MP Biomedicals, Santa Ana, CA, USA) for 2 x 20 seconds at 4.5 m/s^2^. Genomic DNA was extracted using the E.Z.N.A HP Fungal DNA kit (Omega Bio-Tek, Norcross, GA, USA) supplemented with 30 μl RNaseA (Qiagen, Hilden, Germany). For PacBio sequencing, tissue from 10 plates were scraped off the nylon and directly homogenized in a mortar with liquid N_2_. Genomic DNA was extracted using a phenol:chloroform protocol followed by a macro (500 μg) Genomic tip (Qiagen, Hilden Germany) protocol, as described in Skrede *et al* [69].

### Genome sequencing and assembly

In order to get the chromosome location and sequences of mating genes, we first Illumina sequenced the total collection of strains and provided two high-quality representative genomes for the *Trichaptum* (*T*. *abietinum* TA-1010-6-M1 and *T*. *fuscoviolaceum* TF-1002-10-M3) genus by additionally sequencing long reads (PacBio) (S2 Table).

Illumina libraries were generated by the Norwegian Sequencing Centre using the following protocol: 1 μg of genomic DNA was sheared using 96 microTUBE-50 AFA Fiber plates (Covaris Inc., Woburn, MA, USA) on a Covaris E220 system (Covaris Inc., Woburn, MA, USA). The target fragment size was 300–400 bp. gDNA samples were cleaned on a small volume Mosquito liquid handler (TTP labtech) with a 1:1 ratio of Kapa Pure beads (Roche, Basel, Switzerland) and eluted in Tris-Cl, pH 8.0. Library preparation was carried out with 500 ng sheared DNA using Kapa Hyper library prep kit (Roche, Basel, Switzerland). Barcodes were added using the Illumina UD 96 index kit (Illumina). Final libraries were PCR-amplified during 5 cycles with Kapa HIFI PCR kit (Roche, Basel, Switzerland) before standard library quality control with standard sensitivity NGS Fragment kit (Agilent, Santa Clara, CA, USA). Quantification was performed in a qPCR with Kapa Library quantification kit (Roche, Basel, Switzerland). The first batch of library strains were sequenced with HiSeq 4000 system, and the second with NovaSeq I (S2 Table). 2x150 paired-end Illumina reads were generated by both systems. Barcodes and adapters were trimmed from final Illumina sequences using Trim_galore 0.6.5 [70].

PacBio libraries were prepared by the Norwegian Sequencing Centre using Pacific Biosciences Express library preparation protocol (Pacific Biosciences of California, Inc, USA) without any prior fragmentation. Size selection of the final PacBio libraries was performed using BluePippin (Sage Science, Beverly, USA) and 15 Kbp cut-off. PacBio libraries were sequenced on one 1M SMRT cell using Sequel Polymerase v3.0 and sequencing chemistry v3.0. Loading was performed by diffusion and movie time was 600 min for *T*. *abietinum* and 900 min for both *T*. *fuscoviolaceum* runs.

We assembled the genome of reference *T*. *abietinum* using PacBio reads by different assemblers: Flye 2.6 [71], Canu 1.9 [72], MECAT2 [73], SMARTdenovo 1.0.0 [74] and wtdbg2 2.5 [75]. Statistics of draft assemblies using these assemblers can be found in https://perisd.github.io/TriMAT/. Quality of the draft PacBio genome and percentage of consensus between draft genome and Illumina reads were quantified by quast 5.0.2 [76] and polca [77], respectively. The best draft PacBio assembly based on quality statistics, canu (Table 1), was selected and Illumina-corrected using HyPo [78]. Scaffolds with less than 100 PacBio reads of support and less than 10 Kbp of length were removed from the final corrected genome assembly. *T*. *abietinum* ultrascaffolding was done using a Hymenochaetales species, *P*. *noxium* KPN91, which genome was assembled using PacBio reads (Accession No. GCA002287475) [79]. We first checked chromosome correspondence using D-GENIES [80] and manually ultrascaffolded in Geneious 6.1.6 [81]. Chromosomes were named according to *P*. *noxium* chromosome similarity. We applied the same pipeline to the *T*. *fuscoviolaceum* reference assembly, except that ultrascaffolding was performed using RaGOO [82], and the *T*. *abietinum* genome assembly as reference. Visual inspection of syntenic comparisons were performed using mummer 3.23 [83] and D-GENIES. This approach allowed us to correct the order of the ultrascaffolded chromosome 3 of *T*. *abietinum*, which contained 3 scaffolds. We assumed that the order of chromosome 3 must be more similar between sister-species *T*. *abietinum* and *T*. *fuscoviolaceum* than between *T*. *abietinum* and *P*. *noxium*, and the 3 scaffolds were resorted accordingly. The other ultrascaffolded *T*. *abietinum* chromosome 7, reminded untouched. In both *Trichaptum* assemblies, ultrascaffolded chromosomes contain artificial 10,000 Ns separating joined scaffolds. *Trichaptum fuscoviolaceum* chromosomes were composed of multiple scaffolds, except chromosome 5 that was not ultrascaffolded. Details about the ultrascaffolded canu scaffolds can be found in the GitHub page dedicated to this work. Assembly statistics of the final genomes (Table 1), such as N50, genome size, and completeness of universal single copy orthologous genes, were assessed using quast and BUSCO 4.1.2 [84]. The training BUSCO database was agaricomycetes_odb10, which contains 2898 genes. We were able to detect 71% of the telomeric repeats (TTAGGG) [85], 20 and 14 of the 24 expected telomeric regions for each *T*. *abietinum* and *T*. *fuscoviolaceum* reference strains, respectively. For *T*. *abietinum* at least repeats in one telomere was detected for all chromosomes, supporting the 12 chromosome designation for this species, and suggesting that *Trichaptum* genomes were mostly telomere-telomere completed.

Genomes of the 178 strains, sequenced by the Illumina platform, were assembled with iWGS wrapper [86]. We selected assemblies generated by SPAdes 3.14 [87] based on quast quality reports. Genome completeness was assessed with BUSCO. In addition, we included a DOE Joint Genome Institute (JGI) MycoCosm Illumina-sequenced and assembled *T*. *abietinum* strain (L15831, [88]).

### Trichaptum *species classification and species tree reconstruction*

Species designation of strains was first supported based on a fast method, fastANI 1.1 [89]. With fastANI, we calculated the pairwise average nucleotide identity (ANI) among genome assemblies, whose values were then converted to a percentage dissimilarity matrix by subtracting ANI from a value of 100%. The dissimilarity data was used as distance to reconstruct a Neighbor-Joining (NJ) phylogenetic tree in MEGA v5 [90].

The utilization of gene nucleotide and amino acid sequences of universal single copy orthologs annotated with BUSCO assessed the species designation by fastANI. Individual BUSCO protein alignments were generated with MAFFT 7.455 [91]. Amino acid alignments were back translated to nucleotides using pal2nal v14 [92]. Codon columns with gaps were removed from the alignments using trimal 1.4.1 [93]. Gene sequences present in all strains that retained at least 30% of positions and with more than 300 nucleotides (100 amino acids) were selected for additional analyses. In total, 1026 BUSCO genes (35% of the genes) passed our filters. Maximum Likelihood (ML) phylogenetic trees of trimmed genes were reconstructed using IQTree 2.0.3 [94]. The best fitted evolutionary nucleotide model for each gene was estimated by ModelFinder [95] implemented in IQTree. Individual gene trees were pooled in a unique file, which was the input to reconstruct the species tree by applying a coalescent model implemented in ASTRAL 5.7.4 [96]. Species tree branch support was assessed by calculating the gene concordance factor implemented in IQTree. To assess reciprocal monophyly of BUSCO genes, ML phylogenetic trees were read in R using treeio v1.12 [97] and converted to ape v5.4 format [98]. Once species designation were associated to phylogenetic tip labels, the trees were rooted using *T*. *biforme* strain as an outgroup. Monophyly test was performed using spider v1.5 [99]. ML phylogenetic trees of BUSCO genes detected as top 1% in at least two nucleotide diversity statistics (see below) were drawn to a pdf using ggtree v2.2.4 [100].

### Mating gene annotation, alignments and phylogenetics

Mating regions encoding the genes involved in the sexual cycle are conserved among basidiomycetes [36]. We first searched for conserved flanking genes to delimit the mating sites in these new PacBio genomes. Mating A (*MATA*) region was located using *MIP1* (mtDNA intermediate peptidase), *bfg* (beta-flanking gene) and *GLGEN* (Glycogenin-1) gene sequences. Mating B (*MATB*) region was delimited using *PAK* (syn. *CLA4*, serine/threonine protein kinase). We found both mating regions by performing a blast search in Geneious [101] using *P*. *noxium* flanking gene sequences as subject. Delimitation of genes and coding sequences in mating regions were performed using FGENESH and the *P*. *noxium* gene-finding parameters [102]. Some annotated open reading frames (ORFs) required manual curation, as the boundaries of exons vary from one strain to another stochastically. Gene designation of ORFs was assessed by BLASTing the ORF sequences, using the blastx program. An additional annotation comparison to infer the number of exons in different ORFs was done using MAKER2 [103], where we included the transcriptome dataset of L15831 *T*. *abietinum* as input [88].

The annotation of domains and motifs was performed using different strategies. Typical homeodomain/homeobox domains in HD proteins were annotated with CD-search using the CDD v3.18–55570 PSSMs database [104]. To differentiate *HD1* and *HD2* genes, we first screened the nuclear localization signal (NLS) domain using NLS Mapper [105]. NLS is characteristic of HD1 proteins [39,106,107]. Conserved regions enriched in proline amino acids were suggested as potential regions for activation domains (AD) for homeodomain proteins [108]. Coiled coil regions involved in the dimerization of the two homeodomain proteins were detected with Coiled coils v1.1.1 Geneious plugin. Sequence logos for each HD protein domain were generated by using the ggseqlogo v1.0 [109] R package after selecting representative sequences (unique sequences, -c 1) with CD-HIT v4.8.1 [110]. Proteins with seven-transmembrane G protein-coupled receptor superfamily domains are usually indicative of *STE3* pheromone receptors [111]. The 7 transmembrane domains of the pheromone receptor protein were annotated with PredictProtein [112]. Pheromone precursor genes were screened in close proximity to the detected pheromone receptors using pheromone_seeker.pl script [113]. Briefly, the perl script searches common amino acid features encoded in pheromone precursor genes, such as the prenylation signal, or CaaX motif (C, cysteine; aa, two aliphatic amino acids; X is any amino acid) in the C-terminal of the pheromone precursor [40,61]. Hits with a length shorter than 100 bp or longer than 200 bp, and/or distant to *STE3* genes were considered as false positives. Consequently, we removed those hits from the annotations. Additionally, pheromone precursors in strains missing at least one hit close to *STE3*.*2* or *STE3*.*4* were searched using conserved pheromone amino acid sequences of strains in the same clade for *STE3*.*2* or *STE3*.*4* phylogenetic trees. Pheromone maturation sites were located by searching glutamic/arginine (ER) or aspartic acid/arginine (DR) amino acid motifs [39].

Once we had annotated the mating regions in the reference genomes, we were able to search for these genes in the Illumina sequenced and assembled genomes of the rest of strains. We first generated local blast databases for Illumina genomes. We BLASTed the reference flanking genes to pull out the mating regions. In case a mating region (*MATA* or *MATB*) was not contiguous (<43% and <20% of strains for *MATA* and *MATB*, respectively), but split on different contigs, we assumed those regions kept the same gene order as in the reference genomes, and we ultrascaffolded the contigs for each mating region accordingly. 999 Ns were added between joined contigs. Similar to the reference genome assemblies, we defined the mating regions to the scaffold/ultrascaffolded segment containing sequences from *bfg* to *MIP1* for *MATA* region, and from *PAK* to *SNF2* for *MATB*. Once regions were located and/or ultrascaffolded, we used the previous FGENESH pipeline for annotating ORFs. Gene identification was performed by BLASTing the genes from reference genomes against the mating regions. Additional identification was performed by searching family matches in the InterPro-5-RC6 database [114]. All annotations were stored in gff3 files generated by Geneious. Due to limitations of Illumina sequencing some genes in the mating regions were not detected probably because they were not covered by the Illumina reads (S3 Table).

For calculating the frequency of each unique gene block for each region, we followed a conservative approach. We took into account only mating regions that were assembled contiguously by SPAdes and did not need an ultrascaffolding step (S3 Table). The criteria apply from *bfg* to *MIP1* (*MATA*) and from *RIC1* to *SNF2* (*MATB*) genes (S3 Table). Gff3 files were the input to plot *MAT* gene order in R using dplyr 1.0.2, gggenes 3.3.2, ggplot2 3.3.2, and rtracklayer 1.48.0.

To calculate the nucleotide identity conservation of mating regions, we first aligned *MATA* and *MATB* sequence regions independently using FFT-NS-1 algorithm, 200PAM/k = 2 score matrix and default gap opening penalty and offset value with the MAFFT 7.017 version implemented in Geneious. Gaps present in more than 20% of strains were removed with trimal. Identity plots for each region were generated in Geneious.

For phylogenetics, we first generated amino acid sequence alignments using MAFFT and back translated to nucleotides with pal2nal. Again, we were conservatives and codon columns with gaps were removed from the alignments using trimal. The trimmed alignment was converted to amino acid for ML phylogenetic tree reconstruction with IQTree. An evolutionary protein model for each protein was estimated by ModelFinder. Homeodomain and pheromone receptors were classified in clades/allelic classes according to visual inspection of ML phylogenetic trees and pairwise amino acid identity percentages calculated in Geneious. Note here that allelic classes refer to similar protein sequences enclosed in a clade and not to haplotype sequences.

Mating genes, flanking genes and the species tree were plotted with iTOL 6.5 [115]. *T*. *biforme* was used as the outgroup to root the trees when possible. To detect whether a mating related gene was segregating before the speciation event, we selected a random protein sequence of each allelic class to infer the phylogenetic relationship with proteins from other Hymenochaetales species, two reference species of Agaricales and one species from Polyporales.

### Nucleotide statistics, tests to detect balancing selection and recombination

Trimmed codon-based sequence alignments of mating genes, their flanking genes and BUSCO genes were the input for the calculation of nucleotide statistics. Pairwise sequence estimation of synonymous and nonsynonymous substitution rates were calculated using the model of Yang and Nielsen [116] implemented in the yn00 program of PAML 4.9 [117]. We calculated nucleotide statistics, absolute nucleotide divergence (dxy) and relative divergence (Fst) using the PopGenome 2.7.5 package in R 4.0.2 [118]. Sequences were split in different alignments based on the species designation inferred from the species tree phylogeny. Each species-specific alignment was the input to calculate nucleotide diversity (π, Pi) and Tajima’s D using PopGenome. A multilocus test for detecting balancing selection was performed with HKAdirect 0.70b [13]. We generated species-specific input tables for HKAdirect using PopGenome. The input tables consisted of the number of samples (nsam), segregating sites (S), absolute divergence (Divergence) and length for each species-specific gene (length_pol and length_div). We set factor_chrm to 1 because genes are encoded in the nuclear genome. The input tables were necessary to run the multilocus test.

dS and dN boxplots, and genome-wide gene nucleotide statistic plots were generated in R using cowplot 1.0.0, dplyr, ggplot2, ggrepel, PopGenome, reshape2 1.4.4, and rtracklayer.

To detect evidence of recombination, homeodomain and pheromone receptor individual nucleotide alignments were analyzed in RDPv4 [119]. Recombination events significantly detected by all seven methods (RDP, GENECONV, Bootscan, Maxchi, Chimaera, SiSscan and 3Seq) were reported.

### Crosses of monokaryotic strains

To test the compatibility of the inferred mating types, we designed putative compatible and incompatible crosses (S5 Table). Mating types were defined according to S3 Table and based on the phylogenetic analyses and AAI. For example, mating type 158 (A_1_B_56_) is defined by the presence of *MATA*-1 and *MATB*-56 (S3 Table). *MAT*A-1 is the combination of aHD.1 (aHD2 allelic class 1 plus aHD1 allelic class 9, S5 Fig) and bHD.2 (bHD2 allelic class 2 plus bHD1 allelic class 4). And *MATB*-56 is composed by *STE3*.*2* allelic class 5 plus *STE3*.*4* allelic class 10 (S5 Fig). The mating classification was arbitrary. For that reason, for simplicity, selected candidates were described as having or not having a compatible alpha-/beta-complex and *STE3*.*2/STE3*.*4* in S5 Table. We expected a compatible cross when one of the *MATA* complexes (aHD or bHD) and one of the pheromone receptors (STE3.2 or STE3.4) were distinct among the selected strains.

A total of 21 and 10 crosses were designed for crosses within *T*. *abietinum* and *T*. *fuscoviolaceum*, respectively, and 10 crosses between both species. Crosses were performed by plating monokaryons on 3% malt extract agar plates at 4 cm distance between the two monokaryons. After 2–4 weeks, hyphal growth generated contact zones between both monokaryons. Then, a small piece from the middle area of the contact zone was extracted and re-plated on a new 3% malt extract agar plate. After one week of growth, we examined clamp connections by placing a sample of the culture on a slide under a Nikon Eclipse 50i (Nikon Instruments Europe BV, Amsterdam Netherlands). Images of the microscopic slides were acquired under a Zeiss Axioplan-2 imaging with Axiocam HRc microscope camera (Zeiss, Oberkochen Germany). All crosses were performed in triplicates.

### Bioinformatic tools

All bioinformatic tools, programs and most scripts were implemented in UNINETT Sigma2 SAGA High-Performance Computing system (technical details here: https://bit.ly/2VkIXM2), except most R steps. R analyses were performed in Windows 10 operative system, implemented in RStudio 1.3.1073 with an R version 4.0.2. Bioinformatic tools were installed through conda [120] under the SAGA module Anaconda2/2019.03. Non-computational demanding and/or simple python steps were implemented in Jupyter notebooks using python modules installed through conda under Windows 10 Anaconda 1.9.12 version.

### Dryad DOI

https://doi.org/10.5061/dryad.fxpnvx0t4 [122]

## Supporting information

S1 Fig Phylogenetic trees suggest some population structure in *Trichaptum* species. A) Neighbor-Joining tree using the (100 –ANI)/100 values as distances to reconstruct the tree. Scale bar represents (100 –ANI) / 100. B) Coalescent species tree using 1026 BUSCO ML phylogenetic trees. Scale bar represents coalescent units. Bar colors represent the species designation according to the legend. Circles in branches represent the concordance factor support (0: none ML tree agrees– 100: all 1028 ML trees agree). More detailed phylogenetic trees can be found in iTOL: https://itol.embl.de/shared/Peris_D.
(PDF)

S2 Fig Genomes of *T*. *abietinum* and *T*. *fuscoviolaceum* are mostly syntenic. D-GENIES dot-plot of our two reference genomes. Alignment matches are represented by dots and the identity values are colored according to the legend. *MAT* region locations are indicated. Dot identity values are defined as: (number of residue matches for a segment / alignment segment length) * 100. These identity values are calculated from column 10 and 11 in PAF (Pairwise mApping Format) files generated by minimap2 [121], program implemented in D-GENIES.
(PDF)

S3 Fig Amino acid sequence conservation in homeodomain proteins. Sequence logo plots of protein domains involve in the function of homeodomain proteins. Although the C-terminal domain was not related to a function, it was displayed due to its high conservation in protein sequences. Amino acids are colored according to chemistry as indicated in to the legend.
(PDF)

S4 Fig Non-common CpaX motifs were detected in *Trichaptum* pheromone precursor proteins. Phe3.2 and Phe3.4 sequence alignments of unique pheromone precursor proteins are represented in panels A) and B). Sequence logo, generated by Geneious R6, is represented at the top of each alignment to highlight conserved amino acids. Polar amino acids in the CaaX motif are squared in red. Red lines split the pheromone precursor sequences according to the allelic class of the closest mating-related pheromone receptor gene, as indicated in the sequence names on the left (PheX.X.Y, where Y is the allelic class). Letters in sequence names (i.e. -A, -B, etc) indicate unique sequences.
(PDF)

S5 Fig ML protein phylogenetic trees show signals of balancing selection in mating genes and linked genes. ML phylogenetic trees of individual proteins from the *MATA* and *MATB* regions are represented. Species designation and continental isolation are indicated by colored bars according to the legend. Branch support was assessed using the ultrafast bootstrap (UF bootstrap) method. UF bootstrap is indicated in each branch by a gradient color according to the legend. Scale bar is represented in number of amino acid substitutions per site.
(PDF)

S6 Fig Pairwise amino acid identity within mating proteins. Pairwise amino acid identity was calculated for protein sequences within an allelic class and between protein sequences from different allelic classes. Dots represent the average value for within or between pairwise comparisons. Median values for all proteins are represented by horizontal lines inside the boxes, and the upper and lower whiskers represent the highest and lowest values of the 1.5 * IQR (inter-quartile range), respectively. Box plots and dots were colored according to the species where the pairwise comparison was performed. Horizontal dashed line represents the maximum value of 100 - % amino acid identity. We considered 86% amino acid identity a threshold to classify sequences in an allelic class.
(PDF)

S7 Fig Pairwise amino acid identity of mating proteins from strains with identical mating types. Pairwise amino acid identity was calculated for protein sequences within an allelic class of the same species (2 pairwise comparisons for *T*. *fuscoviolaceum*) and between species (2 pairwise comparisons between 2 *T*. *abietinum* and 2 *T*. *fuscoviolaceum*). Dots represent the average value for within or between pairwise comparisons. Horizontal dashed line represents the 86% amino acid identity threshold detected in S5 Fig.
(PDF)

S8 Fig Experimental crosses support predicted compatible and incompatible mating types. Example plate and microscope pictures of the strain cross experiments are displayed on the left and on the right, respectively. Codes on the right, such as TFx1, indicate the type of cross (S5 Table). Pictures of additional crosses are indicated in S5 Table and they can be found in https://perisd.github.io/TriMAT/. When types were distinct in both mating loci clamp connections (red arrows) are observed in septae. Strain names and the inferred allelic classes for each mating gene (S2 Table) are displayed. Compatible *MATA* complexes or pheromone receptors are highlighted in green in each strain. *Tabi*, *Trichaptum abietinum*; *Tfus*, *Trichaptum fuscoviolaceum*.
(PDF)

S9 Fig dS and dN values for mating, flanking and BUSCO genes supports balancing selection in mating genes. Panels A) and C) report the pairwise dS within each species (colored according to the legend) or between species (black) for each gene in the *MATA* and *MATB* regions, respectively. Similarly, panels B) and D) report the pairwise dN. Median values for all genes are represented by horizontal lines inside the boxes, and the upper and lower whiskers represent the highest and lowest values of the 1.5 * IQR (inter-quartile range), respectively. Median values for BUSCO genes are represented by horizontal dashed lines and they are colored according to the legend, green and purple for within *T*. *abietinum* and *T*. *fuscoviolaceum* comparisons, respectively, and black between species comparisons.
(PDF)

S10 Fig Detected BUSCO genes are shown to have some signal of non-reciprocal monophyly. Maximum-Likelihood phylogenetic trees of five detected BUSCO genes based on nucleotide statistics (Fig 7) are represented. Scale bar is represented in number of nucleotide substitutions per site.
(PDF)

S11 Fig Geographic distribution of mating alleles supports long-term segregation. Stacked bar plots are represented for each mating gene. For each allelic class a bar colored according to the geographic location is drawn.
(PDF)

S12 Fig Two recent duplications of *aHD2* genes generated xHD2 proteins. ML phylogenetic trees of a protein sequence alignment containing xHD2, aHD2 and bHD2. xHD2 sequences are highlighted with red arrows. Branch support was assessed using the ultrafast bootstrap (UF bootstrap) method. UF bootstrap is indicated in each branch by a gradient color according to the legend. Scale bar is represented in number of amino acid substitutions per site.
(PDF)

S13 Fig Some mating alleles are older than *Trichaptum* genus. Selected regions of ML phylogenetic trees of trimmed (trimal–gt 0.8) protein sequence alignments containing HD2-HD1 and STE3 are displayed in panels A) and B), respectively. Branch support was assessed using the ultrafast bootstrap (UF bootstrap) method. UF bootstrap is indicated in each branch by a gradient color according to the legend. Scale bar is represented in number of amino acid substitutions per site. *Trichaptum* proteins are highlighted by red arrows or enclosed in a red bar. Allelic classes are indicated in the protein name (i.e. aHDX.Y, where Y is the allelic class). Protein sequences were retrieved from DOE-JGI MycoCosm and download from NCBI as indicated: 1. Hymneochaetales JGI protein list: Fomme: *Fomitiporia mediterranea* (MF3/22), Onnsc: *Onnia scaura* (P-53A), Phefer: *Phellinidium ferrugineofuscum* (SpK3Phefer14), Pheign: *Phellinus ignarius* (CCBS575), Phevit: *Phellinus viticola* (PhevitSig-SM15), Pheni: *Phellopilus* (*Phellinus*) *nigrolimitatus* (SigPhenig9), Porchr: *Porodaedalea chrysoloma* (FP-135951), Pornie: *Porodaedalea niemelaei* (PN71-100-IP13), Resbic: *Resinicium bicolor* (OMC78), Ricfib: *Rickenella fibula* (HBK330-10), Ricmel: *Rickenella mellea* (SZMC22713), Schpa: *Schizopora paradoxa* (KUC8140), Sidvul: *Sidera vulgaris* (OMC1730). 2. Downloaded from NCBI: [HYMENOCHAETALES] *Fomitiporia mediterranea* (MF3/22), *Pyrrhoderma noxium* (KPN91), *Shanghuangporus baumii* (Bpt 821), *Rickenella mellea* (SZMC22713); [AGARICALES] *Laccaria bicolor* (S238N-H82), *Coprinopsis cinerea* (Okayama7#130); [POLYPORALES] *Rhodonia* (*Postia*) *placenta* (Mad-698-R). To remove protein redundancy in protein collection of species retrieved from JGI, a blastp using the downloaded NCBI protein sequences and HDs and STE3s protein representatives of each allelic class was performed. For each input sequence two hits were used for sequence alignments, a protein sequence with the lowest e-value and the protein sequence with the highest coverage value. Complete ML phylogenetic trees are deposited in a shared iTOL folder: https://itol.embl.de/shared/Peris_D.
(PDF)

S1 Table Strains used in this study. Geographical and source of isolation.
(XLSX)

S2 Table Whole genome sequencing statistics. (XLSX)

S3 Table Mating gene allelic designation and predicted mating types. (XLSX)

S4 Table Sequences detected as recombinant. (XLSX)

S5 Table dS and dN average pairwise comparisons between mating genes and BUSCO genes (dX Gene/dX BUSCO). (XLSX)

S6 Table Experimental mating crosses. (XLSX)
