# Supplementary material for: Large-scale fungal strain sequencing unravels the molecular diversity in mating loci maintained by long-term balancing selection
Source: PLoS Genet. 2022 Mar 31;18(3):e1010097. doi: 10.1371/journal.pgen.1010097 (PMC8970355; doi:10.1371/journal.pgen.1010097)
Supplement: S1 Fig — A) Neighbor-Joining tree using the (100 –ANI)/100 values as distances to reconstruct the tree. Scale bar represents (100 –ANI) / 100. B) Coalescent species tree using 1026 BUSCO ML phylogenetic trees. Scale bar represents coalescent units. Bar colors represent the species designation according to the legend. Circles in branches represent the concordance factor support (0: none ML tree agrees– 100: all 1028 ML trees agree). More detailed phylogenetic trees can be found in iTOL: https://itol.embl.de/shared/Peris_D. (PDF) [file pgen.1010097.s001.pdf]

A

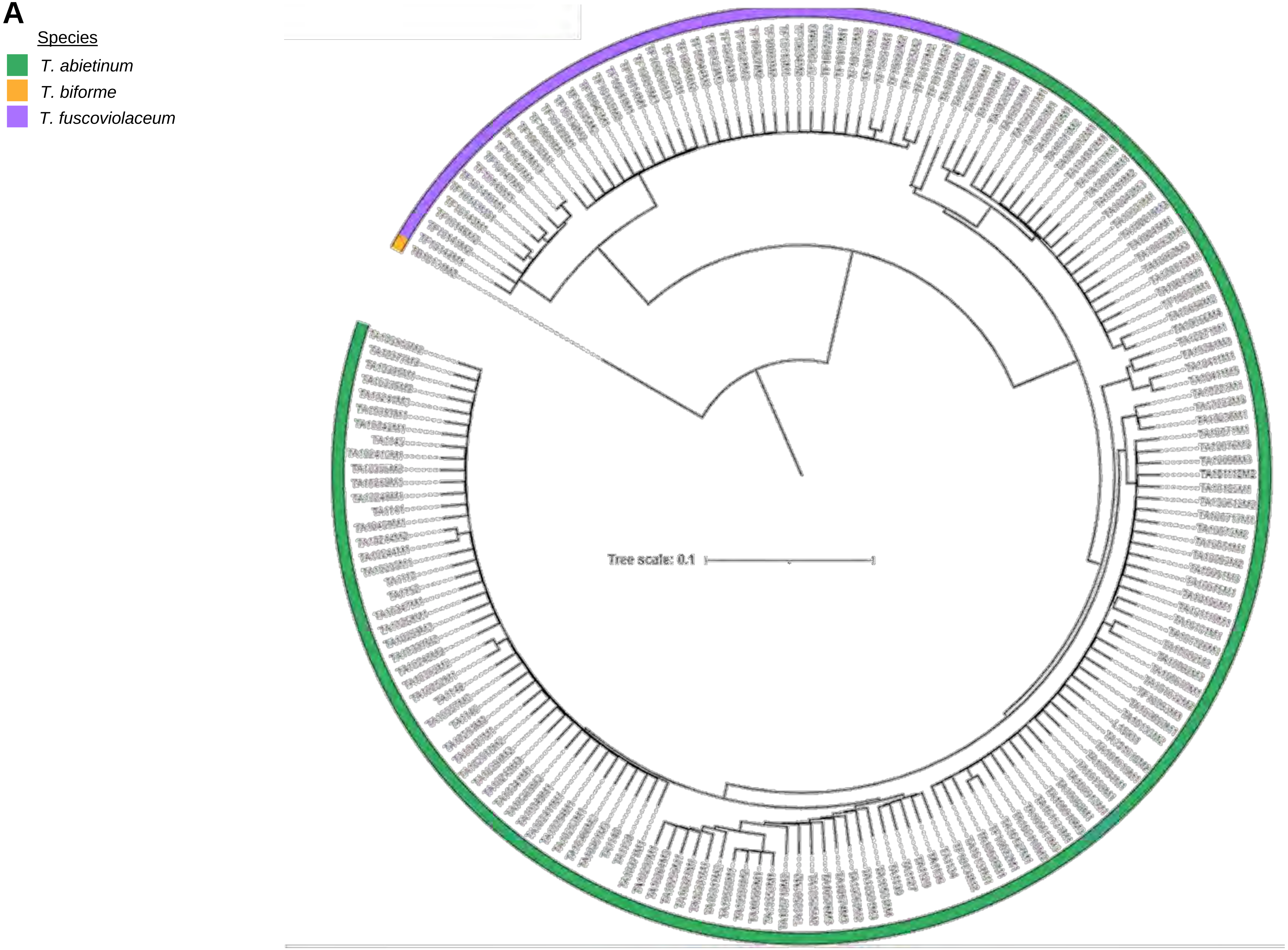
Species
T. abietinum
T. biforme
T. fuscoviolaceum
Tree scale: 0.1

B

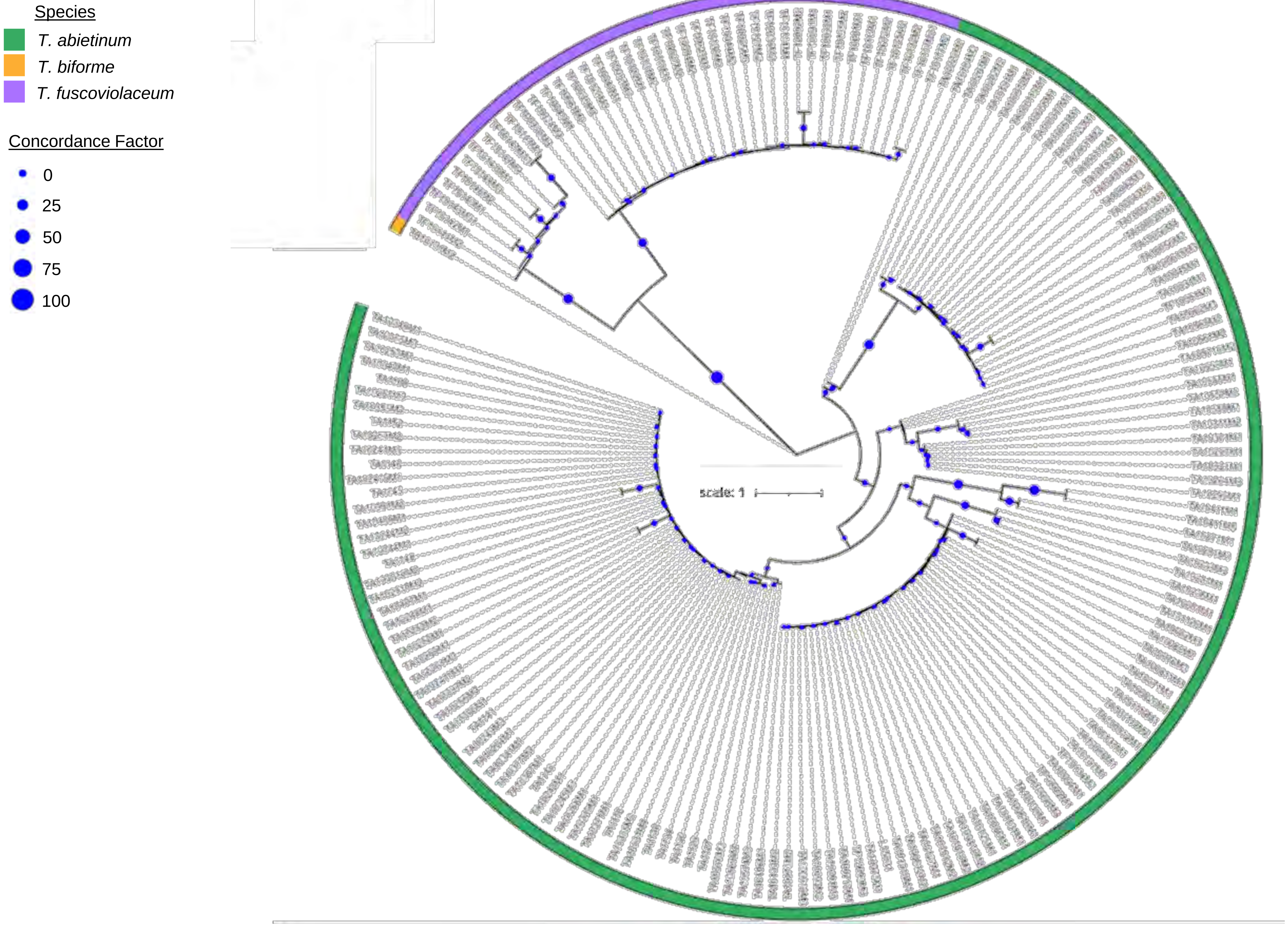
Species
T. abietinum
T. biforme
T. fuscoviolaceum
Concordance Factor
0
25
50
75
100
scale: 1
