# Supplementary figures and images for: Large-scale fungal strain sequencing unravels the molecular diversity in mating loci maintained by long-term balancing selection
Source: PLoS Genet. 2022 Mar 31;18(3):e1010097. doi: 10.1371/journal.pgen.1010097 (PMC8970355; doi:10.1371/journal.pgen.1010097)

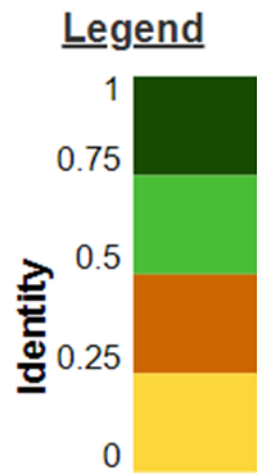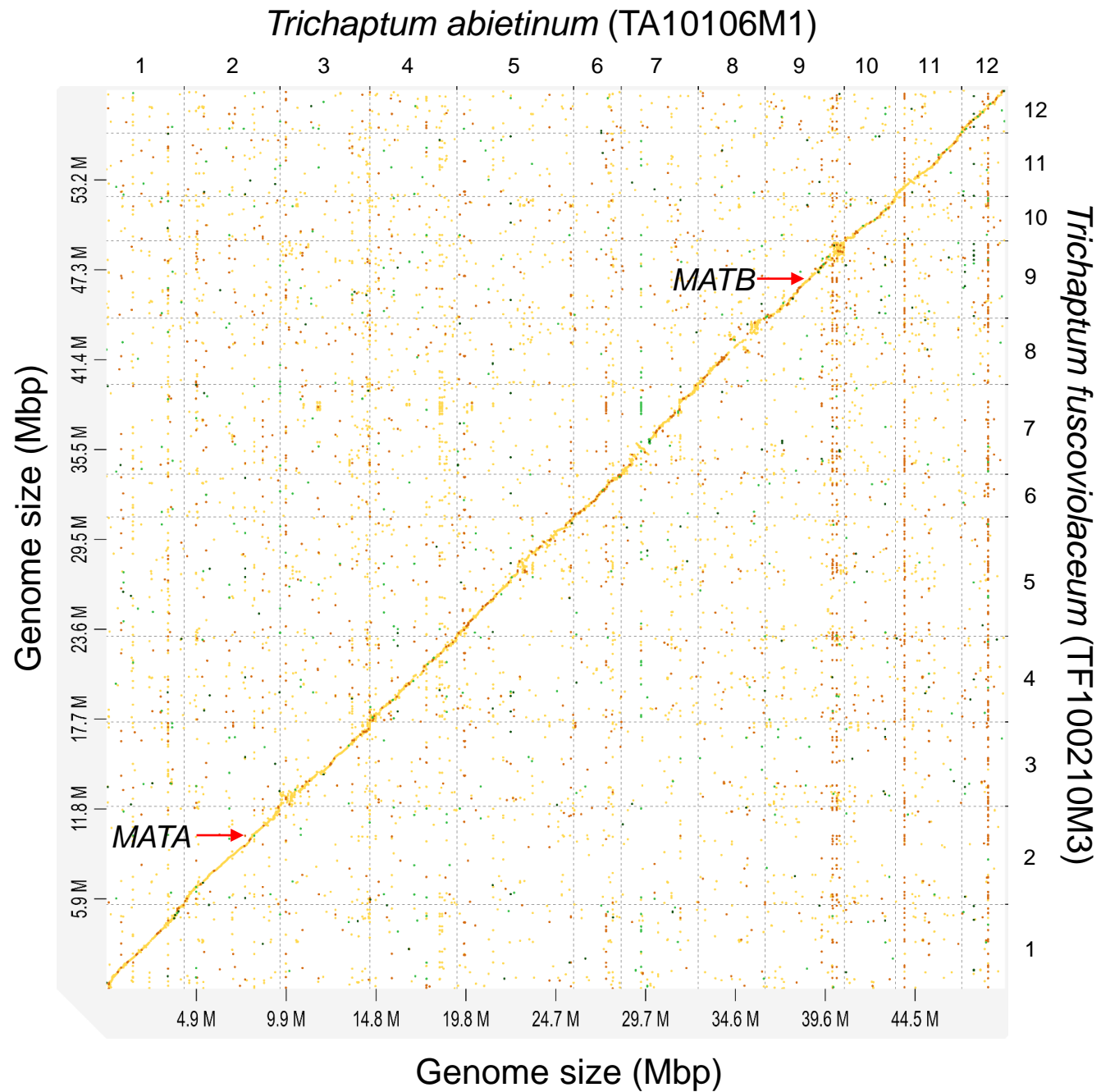

Supplement: S2 Fig — D-GENIES dot-plot of our two reference genomes. Alignment matches are represented by dots and the identity values are colored according to the legend. MAT region locations are indicated. Dot identity values are defined as: (number of residue matches for a segment / alignment segment length) * 100. These identity values are calculated from column 10 and 11 in PAF (Pairwise mApping Format) files generated by minimap2 [121], program implemented in D-GENIES. (PDF) [file pgen.1010097.s002.pdf]

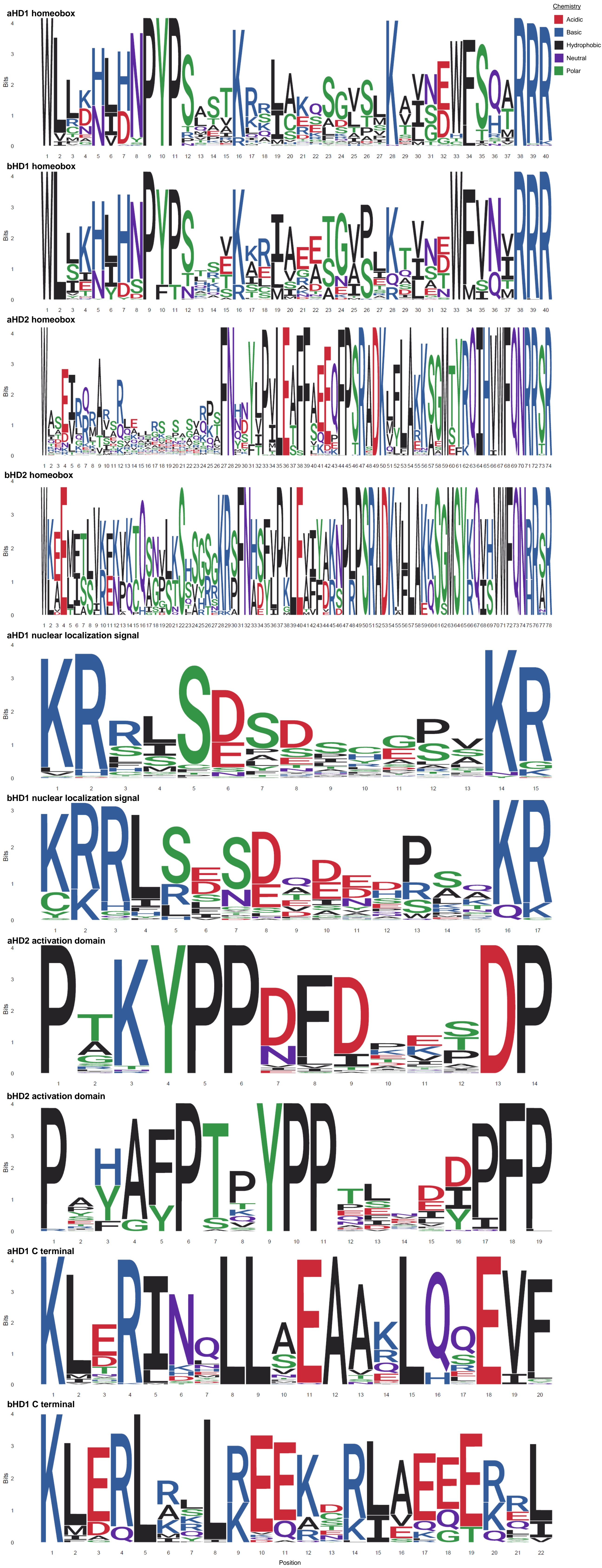

Supplement: S3 Fig — Sequence logo plots of protein domains involve in the function of homeodomain proteins. Although the C-terminal domain was not related to a function, it was displayed due to its high conservation in protein sequences. Amino acids are colored according to chemistry as indicated in to the legend. (PDF) [file pgen.1010097.s003.pdf]

A

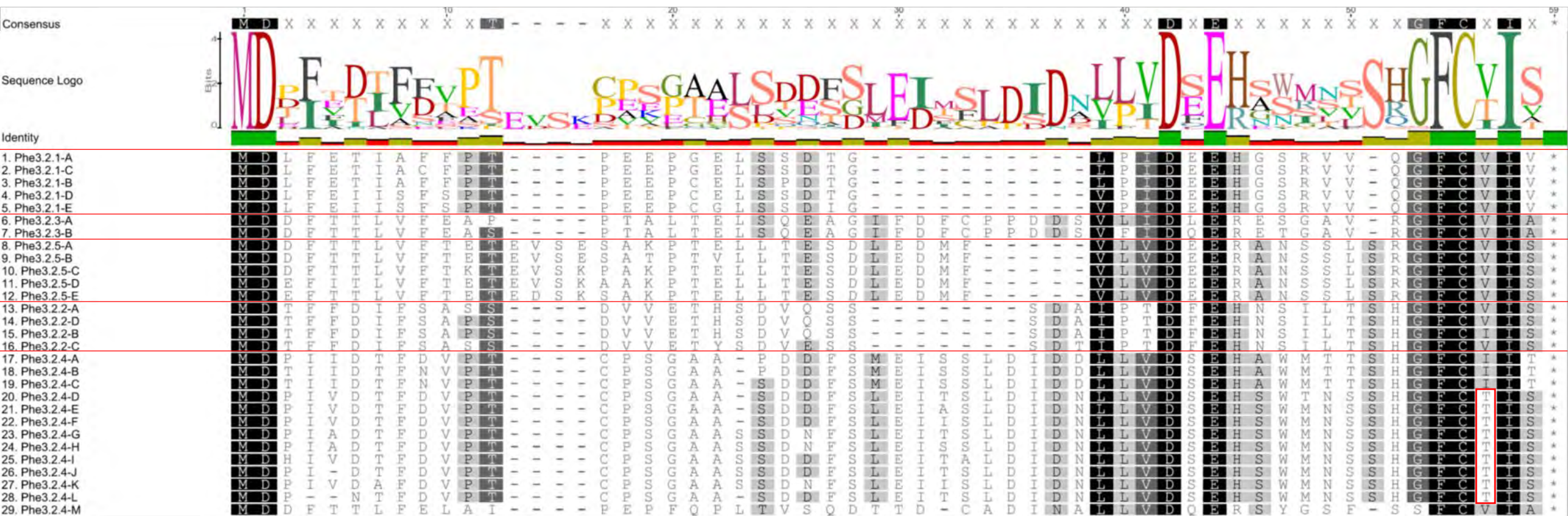

Maturation  
site

CaaX

□ Non-aliphatic amino acids in the CaaX motif

B

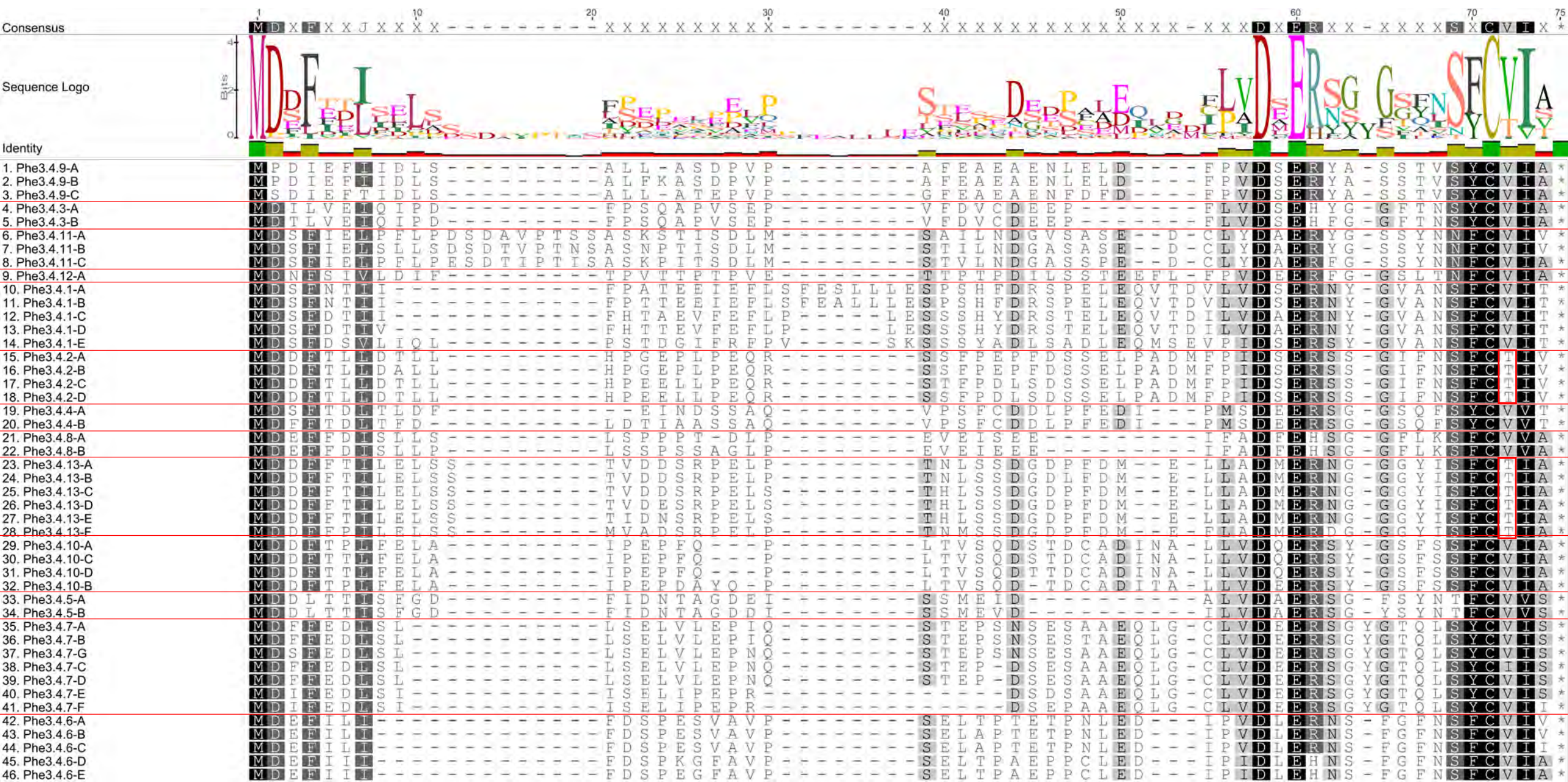

Supplement: S4 Fig — Phe3.2 and Phe3.4 sequence alignments of unique pheromone precursor proteins are represented in panels A) and B). Sequence logo, generated by Geneious R6, is represented at the top of each alignment to highlight conserved amino acids. Polar amino acids in the CaaX motif are squared in red. Red lines split the pheromone precursor sequences according to the allelic class of the closest mating-related pheromone receptor gene, as indicated in the sequence names on the left (PheX.X.Y, where Y is the allelic class). Letters in sequence names (i.e. -A, -B, etc) indicate unique sequences. (PDF) [file pgen.1010097.s004.pdf]

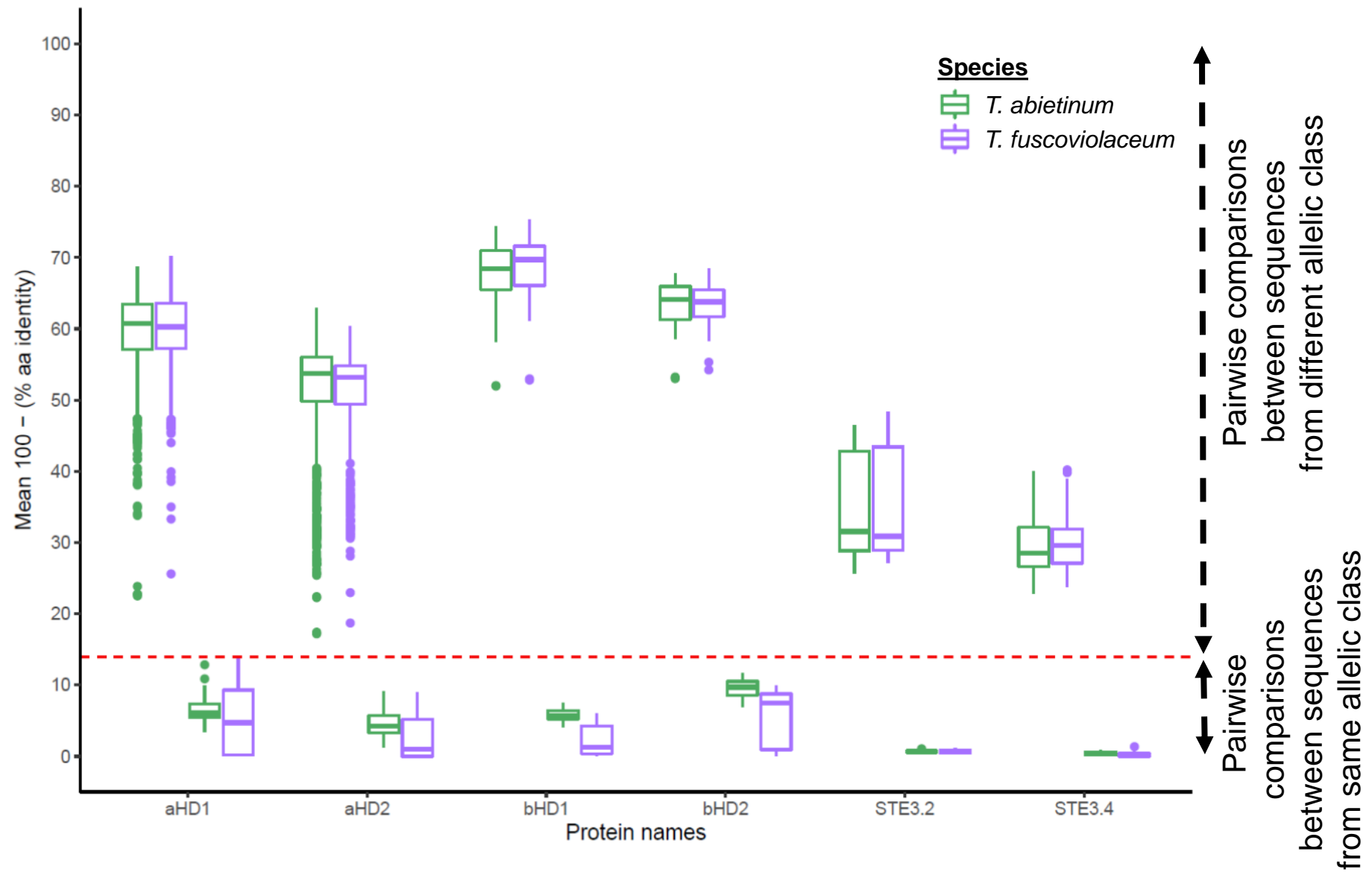

Supplement: S6 Fig — Pairwise amino acid identity was calculated for protein sequences within an allelic class and between protein sequences from different allelic classes. Dots represent the average value for within or between pairwise comparisons. Median values for all proteins are represented by horizontal lines inside the boxes, and the upper and lower whiskers represent the highest and lowest values of the 1.5 * IQR (inter-quartile range), respectively. Box plots and dots were colored according to the species where the pairwise comparison was performed. Horizontal dashed line represents the maximum value of 100 - % amino acid identity. We considered 86% amino acid identity a threshold to classify sequences in an allelic class. (PDF) [file pgen.1010097.s006.pdf]

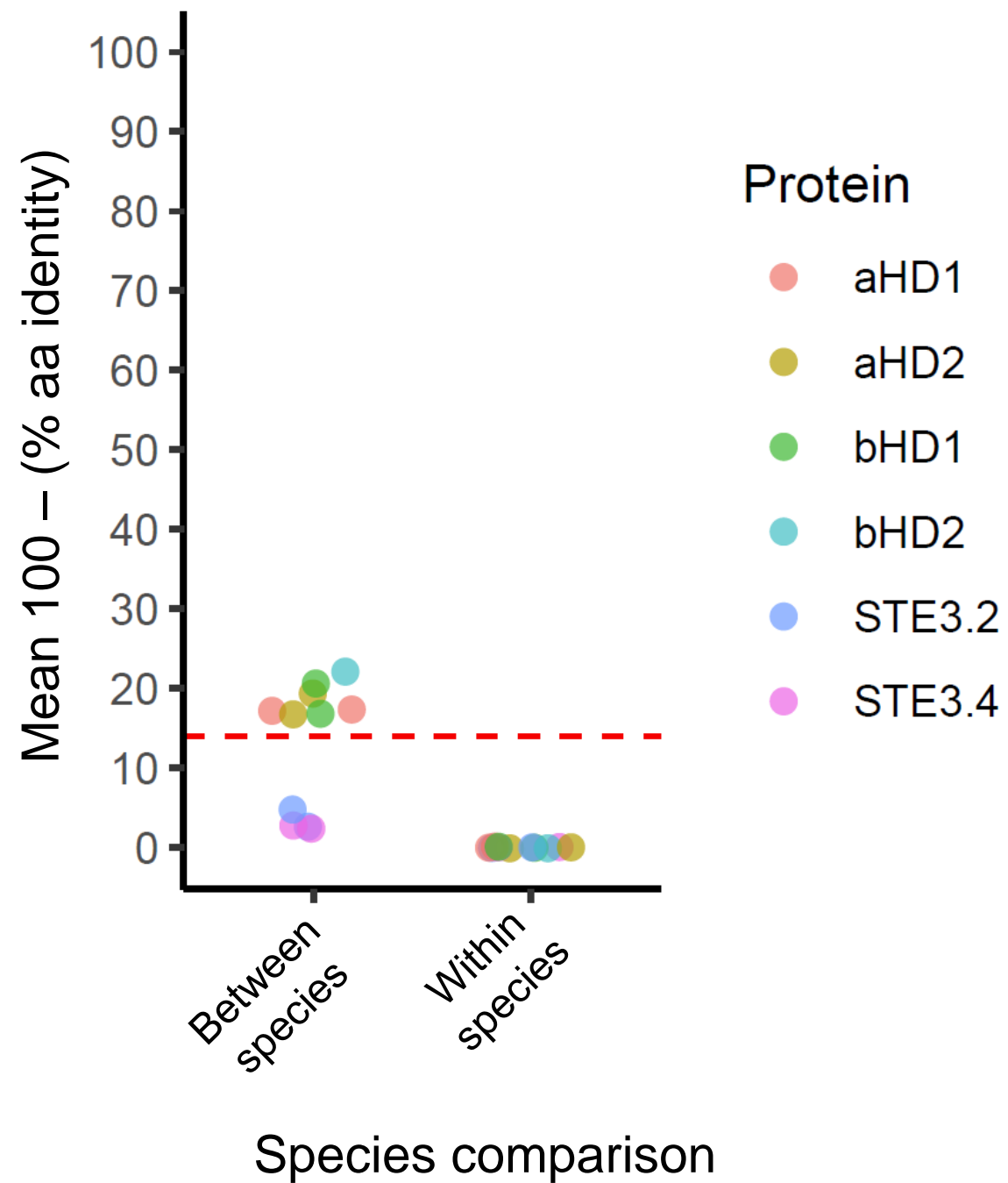

Supplement: S7 Fig — Pairwise amino acid identity was calculated for protein sequences within an allelic class of the same species (2 pairwise comparisons for T. fuscoviolaceum) and between species (2 pairwise comparisons between 2 T. abietinum and 2 T. fuscoviolaceum). Dots represent the average value for within or between pairwise comparisons. Horizontal dashed line represents the 86% amino acid identity threshold detected in S5 Fig. (PDF) [file pgen.1010097.s007.pdf]

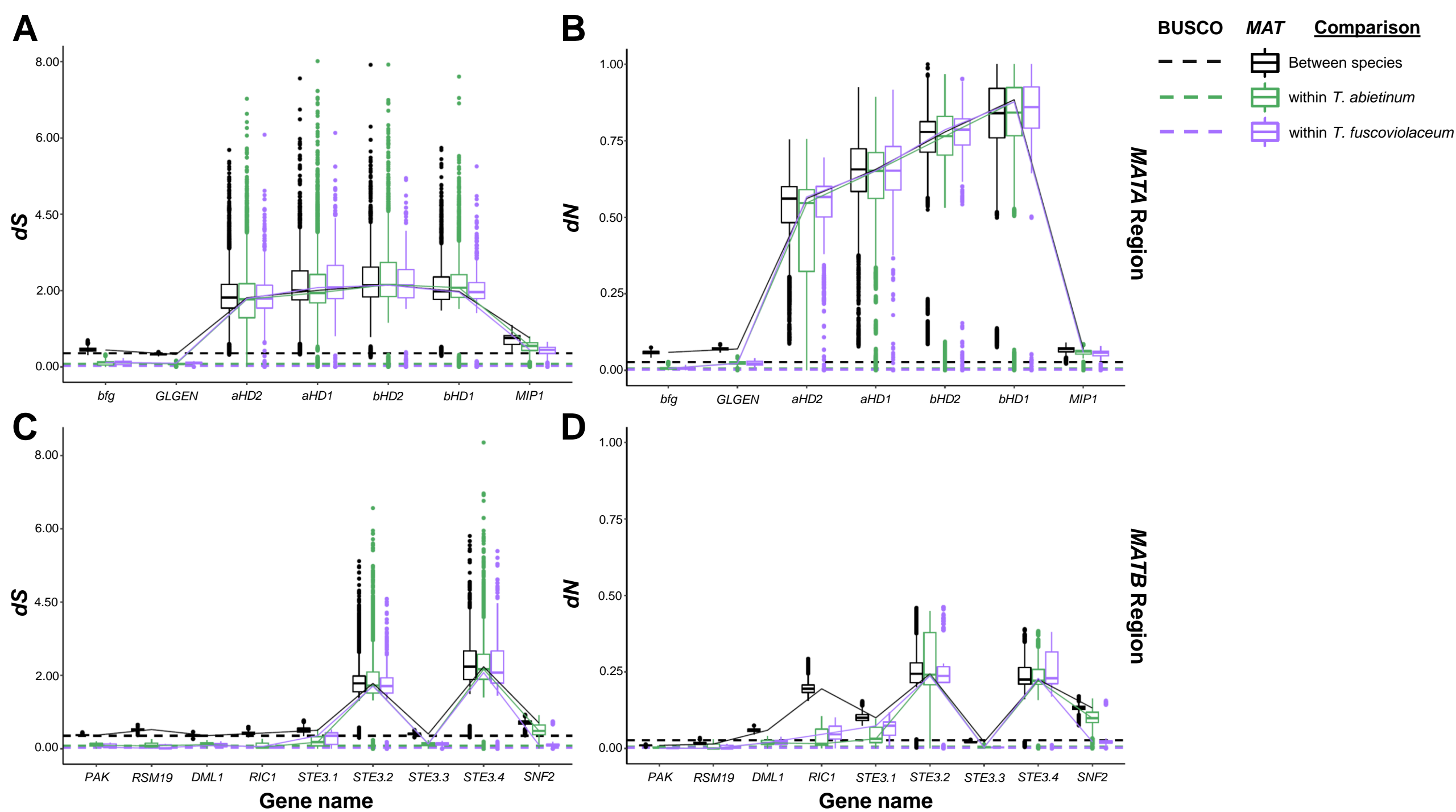

Supplement: S9 Fig — Panels A) and C) report the pairwise dS within each species (colored according to the legend) or between species (black) for each gene in the MATA and MATB regions, respectively. Similarly, panels B) and D) report the pairwise dN. Median values for all genes are represented by horizontal lines inside the boxes, and the upper and lower whiskers represent the highest and lowest values of the 1.5 * IQR (inter-quartile range), respectively. Median values for BUSCO genes are represented by horizontal dashed lines and they are colored according to the legend, green and purple for within T. abietinum and T. fuscoviolaceum comparisons, respectively, and black between species comparisons. (PDF) [file pgen.1010097.s009.pdf]

27296at155619

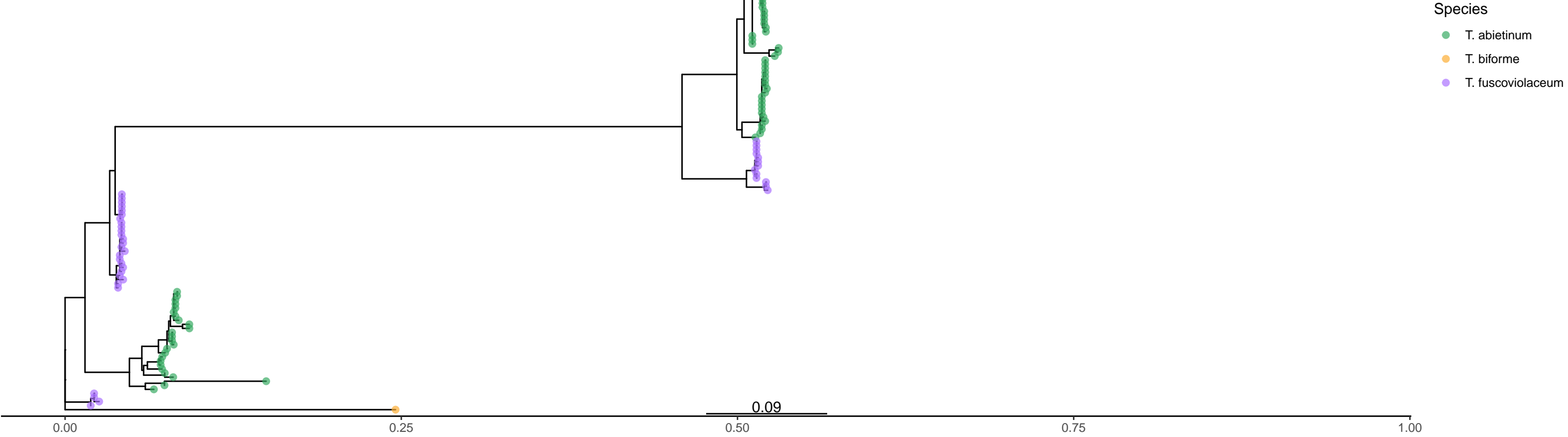

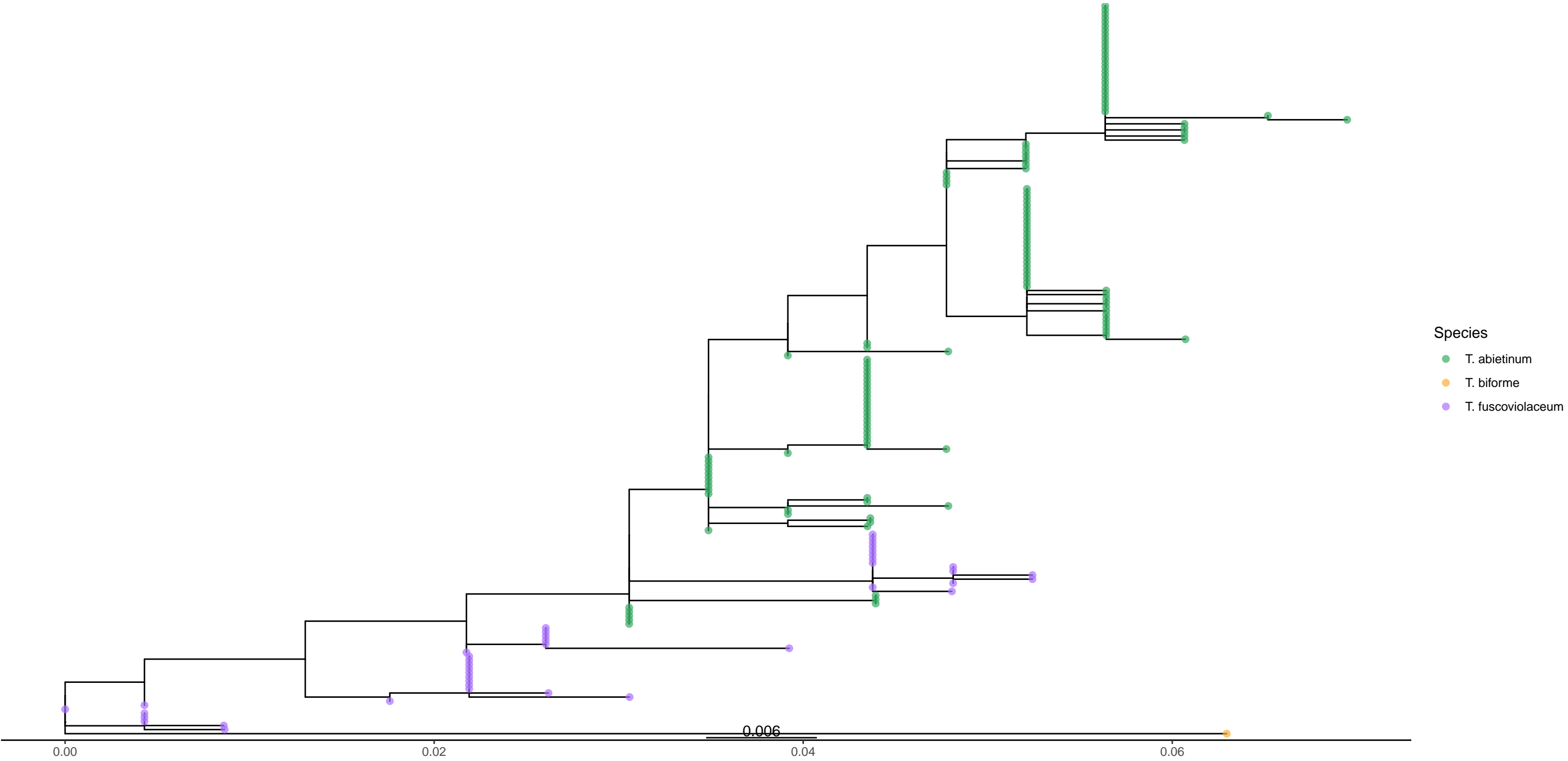

6755at155619

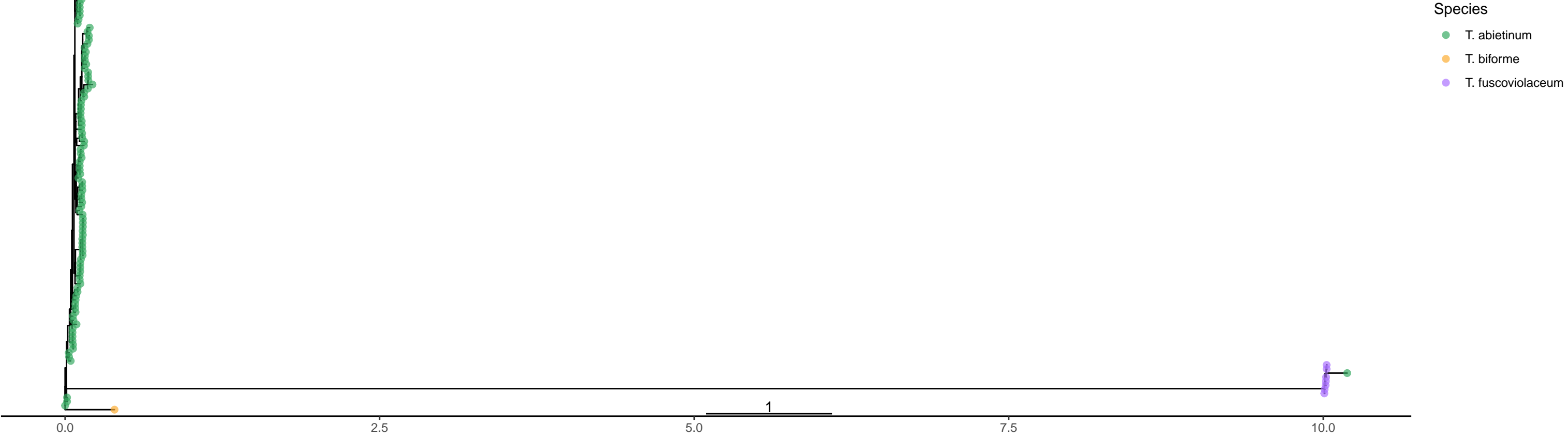

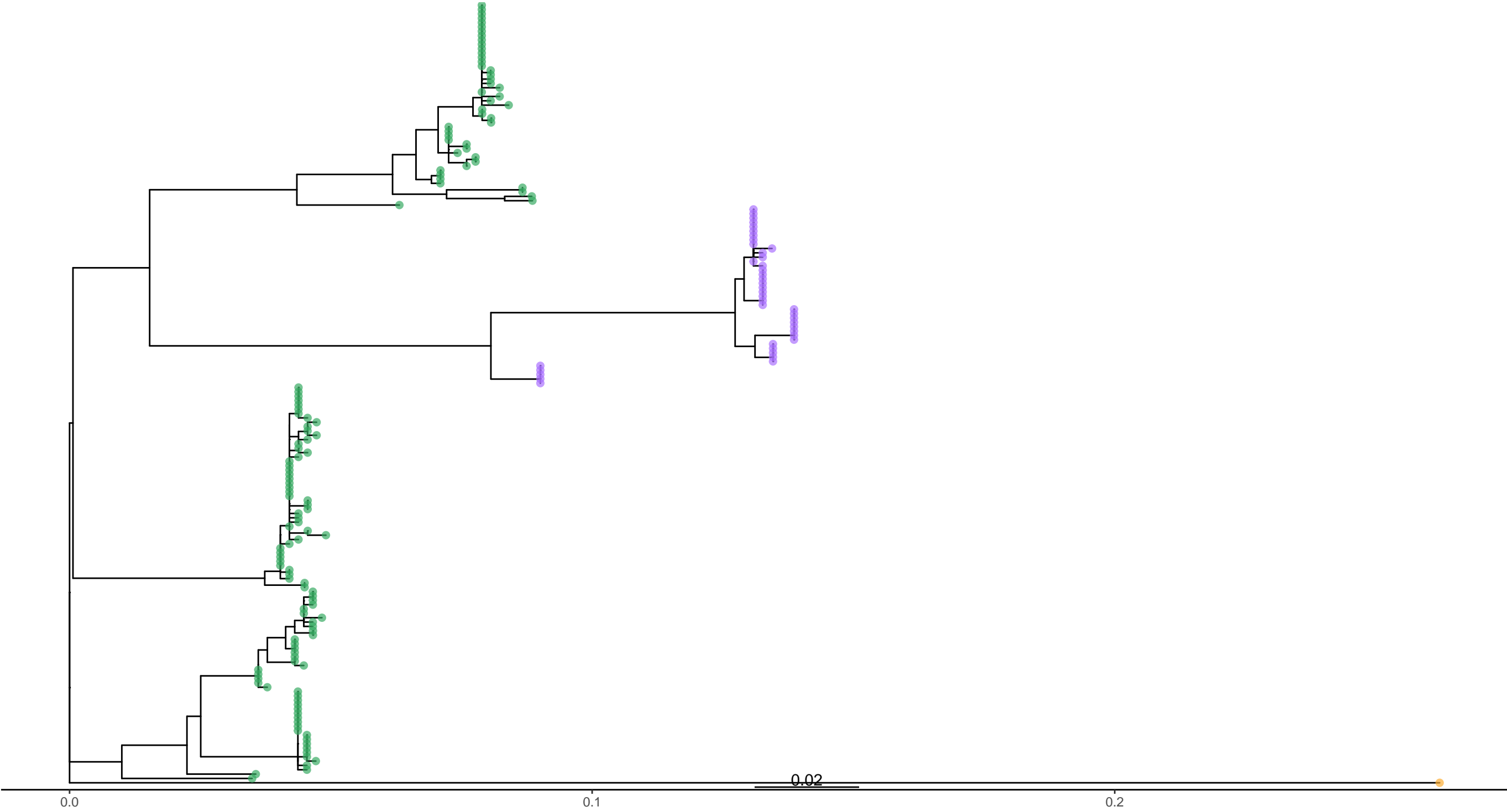

41864at155619

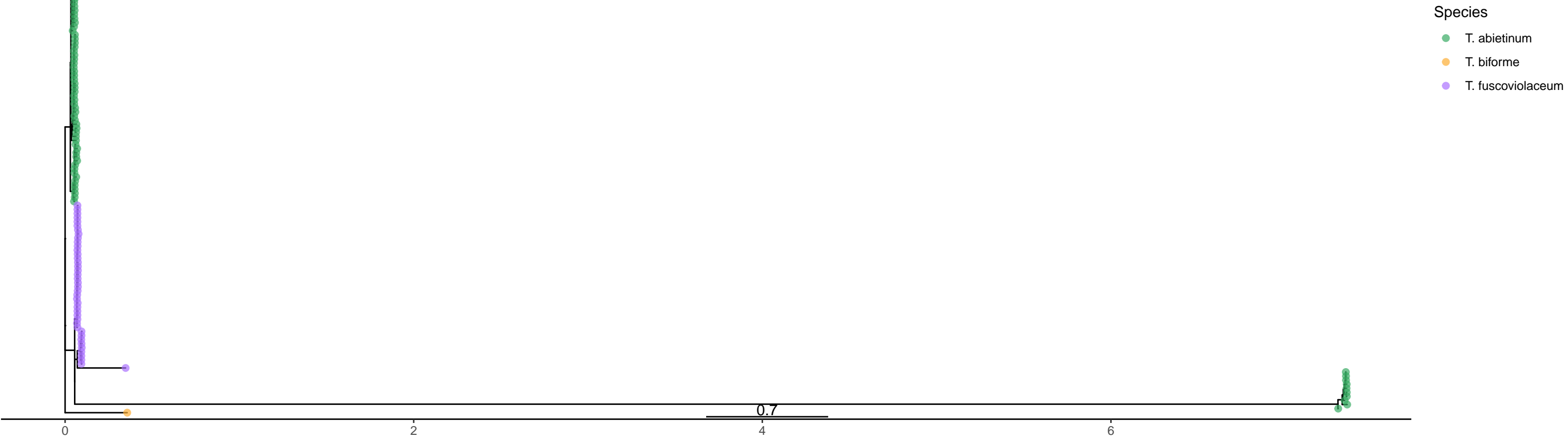

Supplement: S10 Fig — Maximum-Likelihood phylogenetic trees of five detected BUSCO genes based on nucleotide statistics (Fig 7) are represented. Scale bar is represented in number of nucleotide substitutions per site. (PDF) [file pgen.1010097.s010.pdf]

A

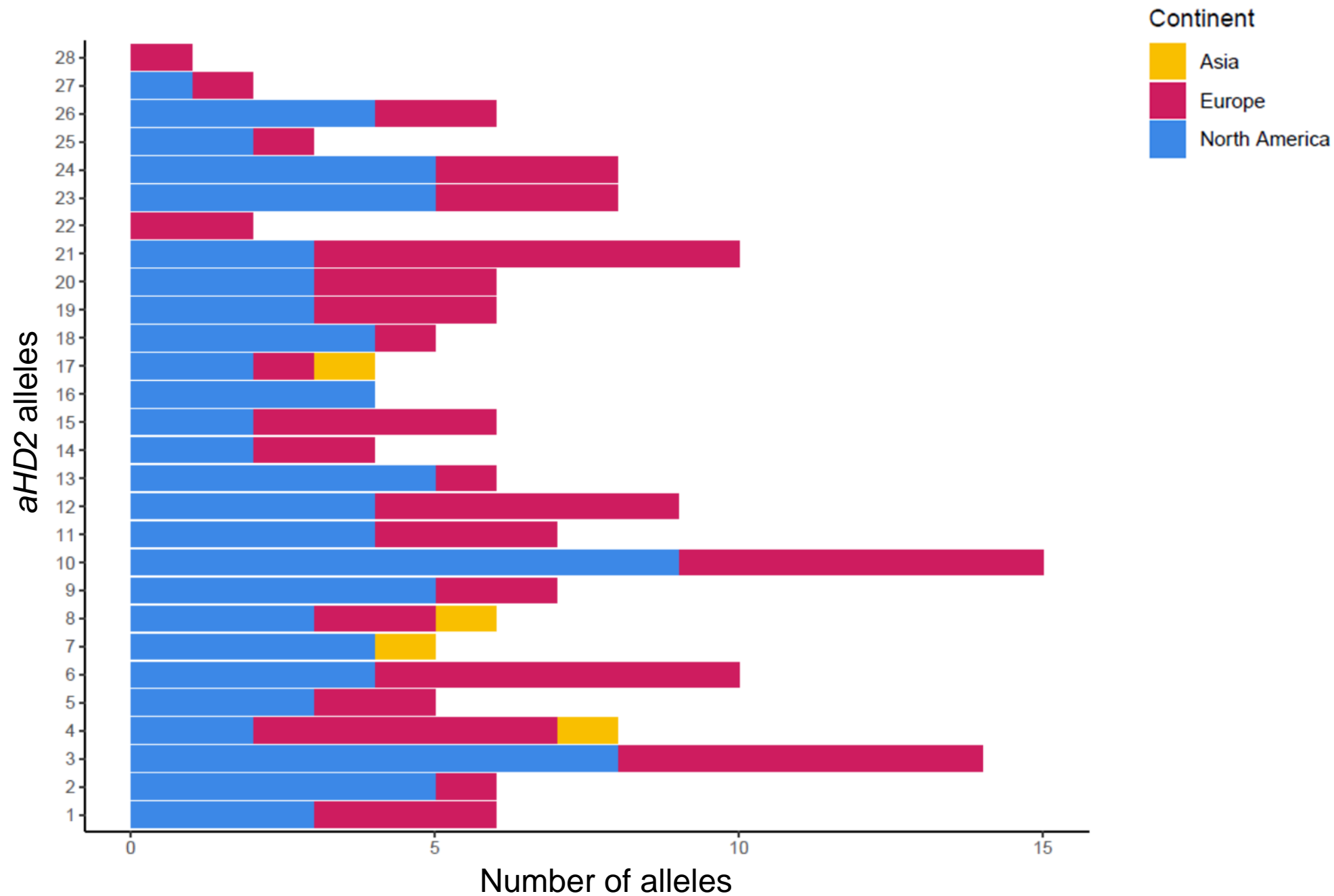

B

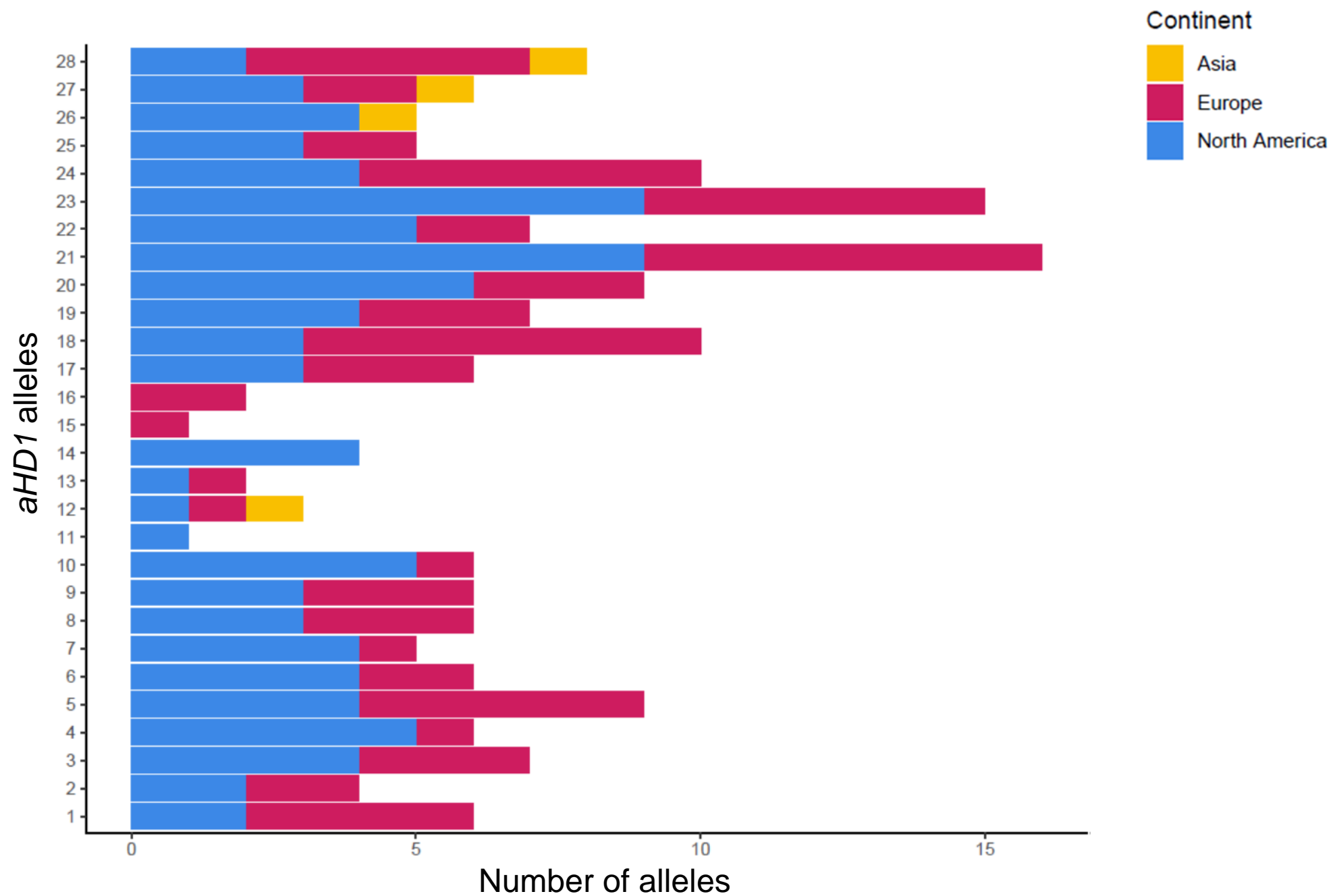

C

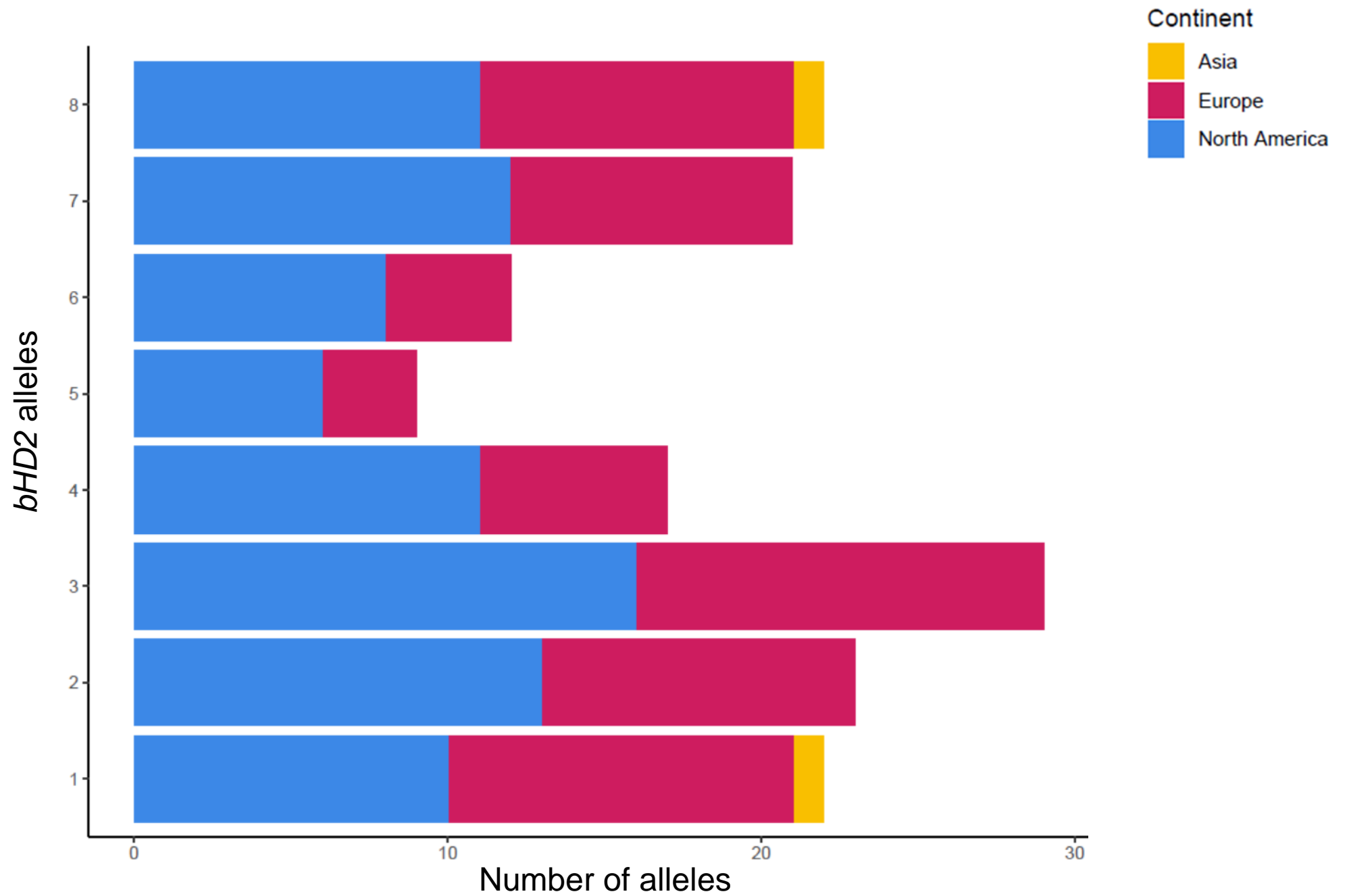

D

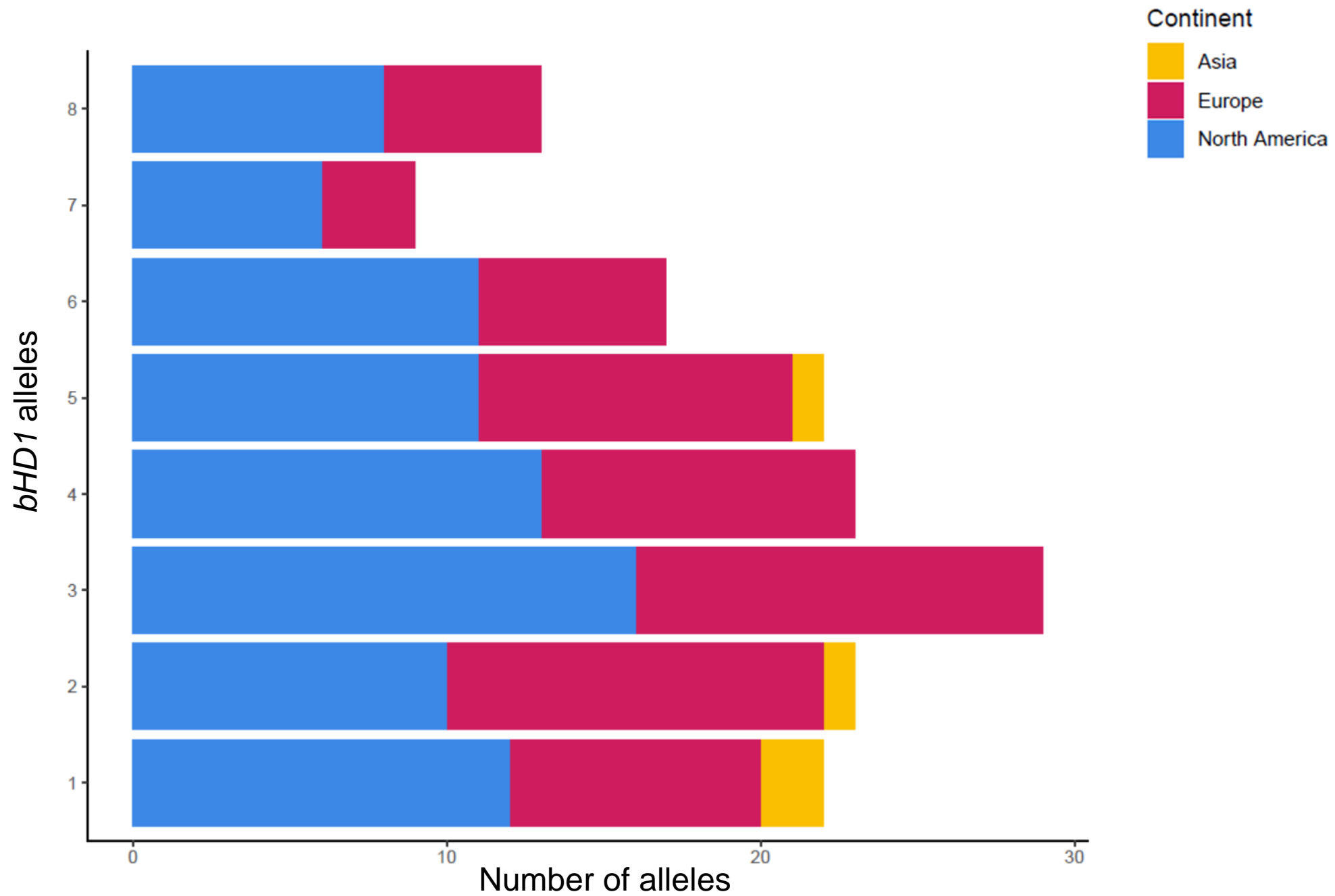

E

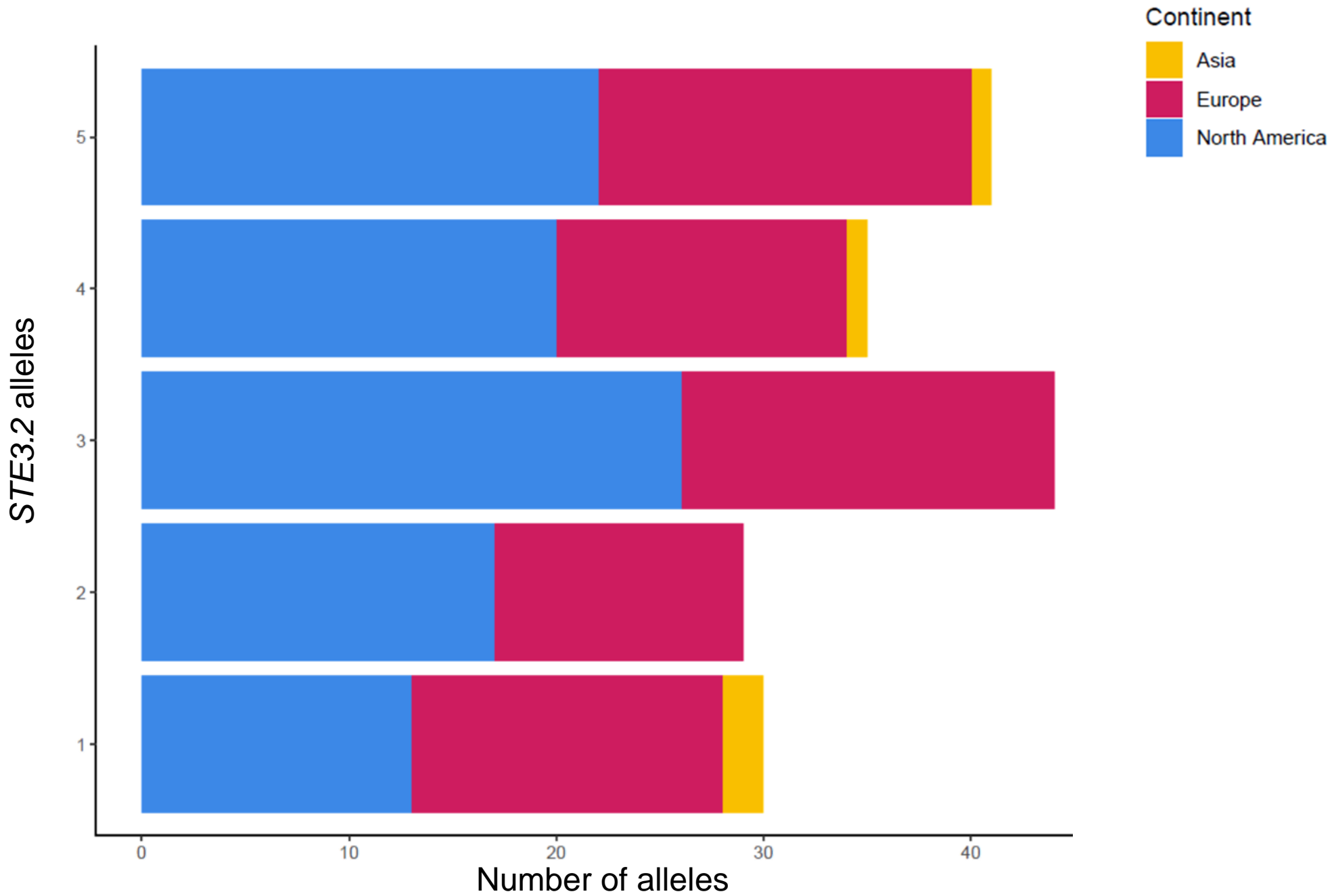

F

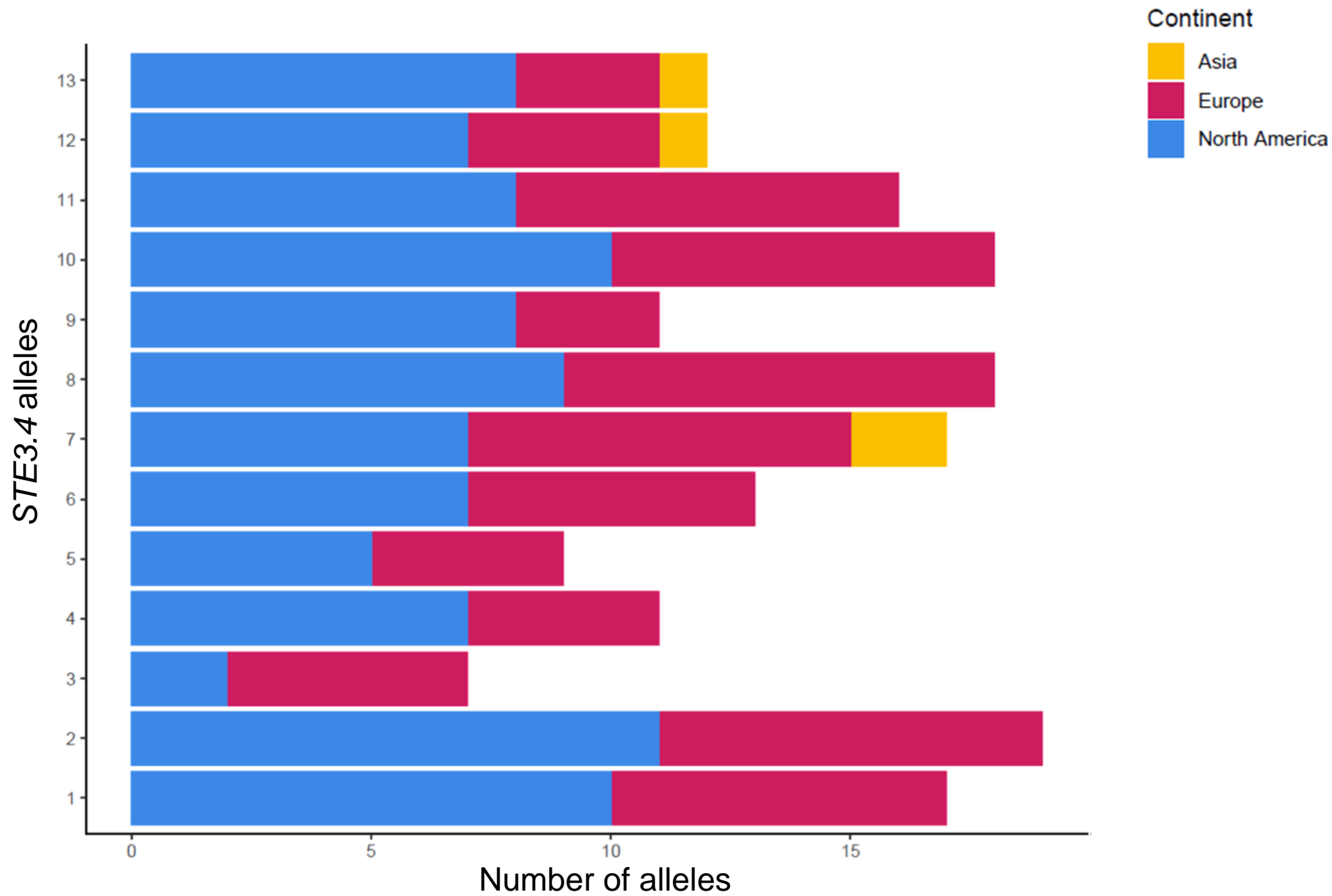

Supplement: S11 Fig — Stacked bar plots are represented for each mating gene. For each allelic class a bar colored according to the geographic location is drawn. (PDF) [file pgen.1010097.s011.pdf]

Tree scale: 1

→ *xHD2*

UF Bootstrap

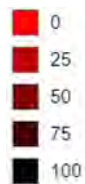

Duplication of aHD2.10

Duplication of aHD2.8

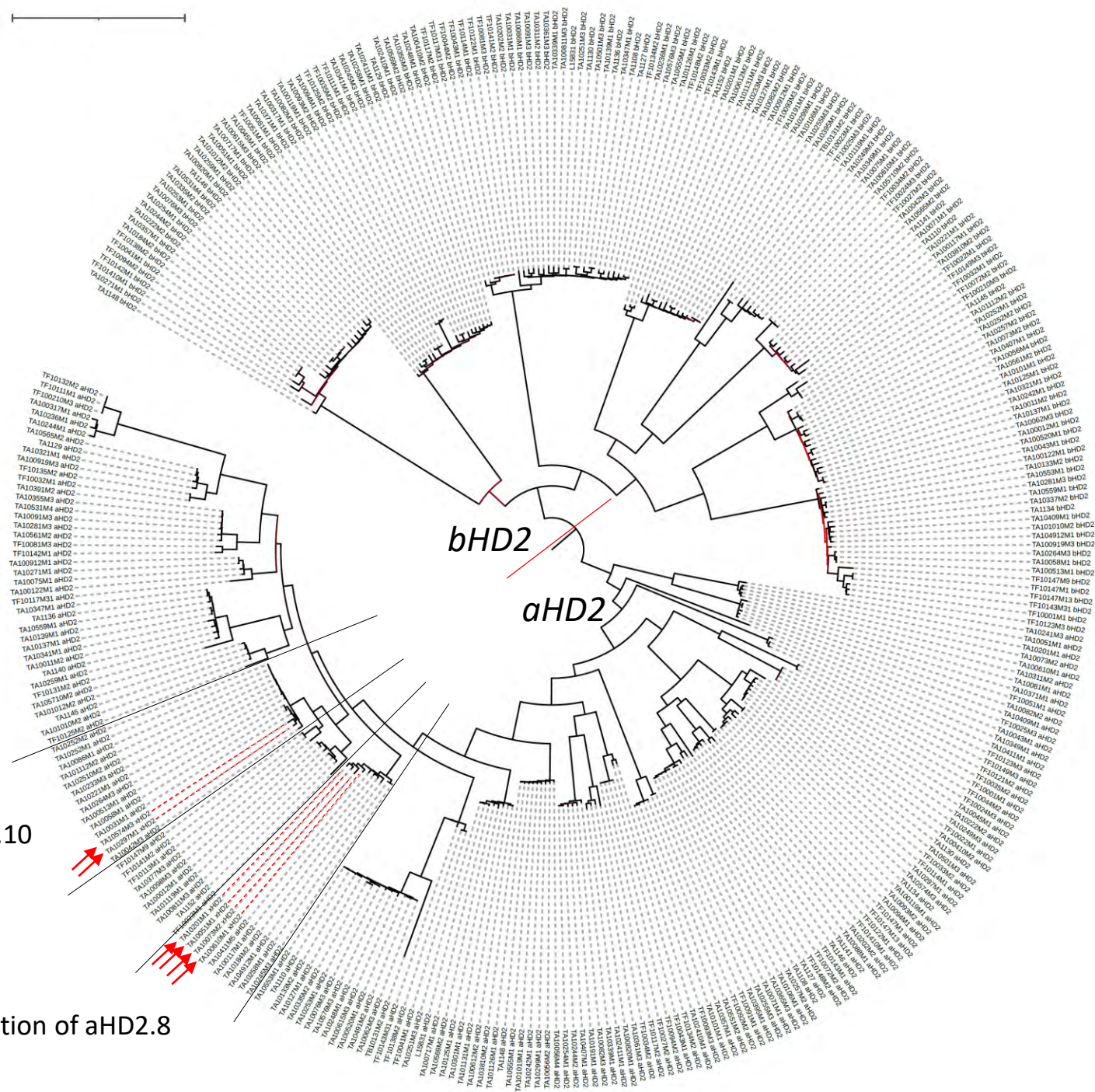

Supplement: S12 Fig — ML phylogenetic trees of a protein sequence alignment containing xHD2, aHD2 and bHD2. xHD2 sequences are highlighted with red arrows. Branch support was assessed using the ultrafast bootstrap (UF bootstrap) method. UF bootstrap is indicated in each branch by a gradient color according to the legend. Scale bar is represented in number of amino acid substitutions per site. (PDF) [file pgen.1010097.s012.pdf]
