# Supplementary material for: Large-scale fungal strain sequencing unravels the molecular diversity in mating loci maintained by long-term balancing selection
Source: PLoS Genet. 2022 Mar 31;18(3):e1010097. doi: 10.1371/journal.pgen.1010097 (PMC8970355; doi:10.1371/journal.pgen.1010097)
Supplement: S5 Fig — ML phylogenetic trees of individual proteins from the MATA and MATB regions are represented. Species designation and continental isolation are indicated by colored bars according to the legend. Branch support was assessed using the ultrafast bootstrap (UF bootstrap) method. UF bootstrap is indicated in each branch by a gradient color according to the legend. Scale bar is represented in number of amino acid substitutions per site. (PDF) [file pgen.1010097.s005.pdf]

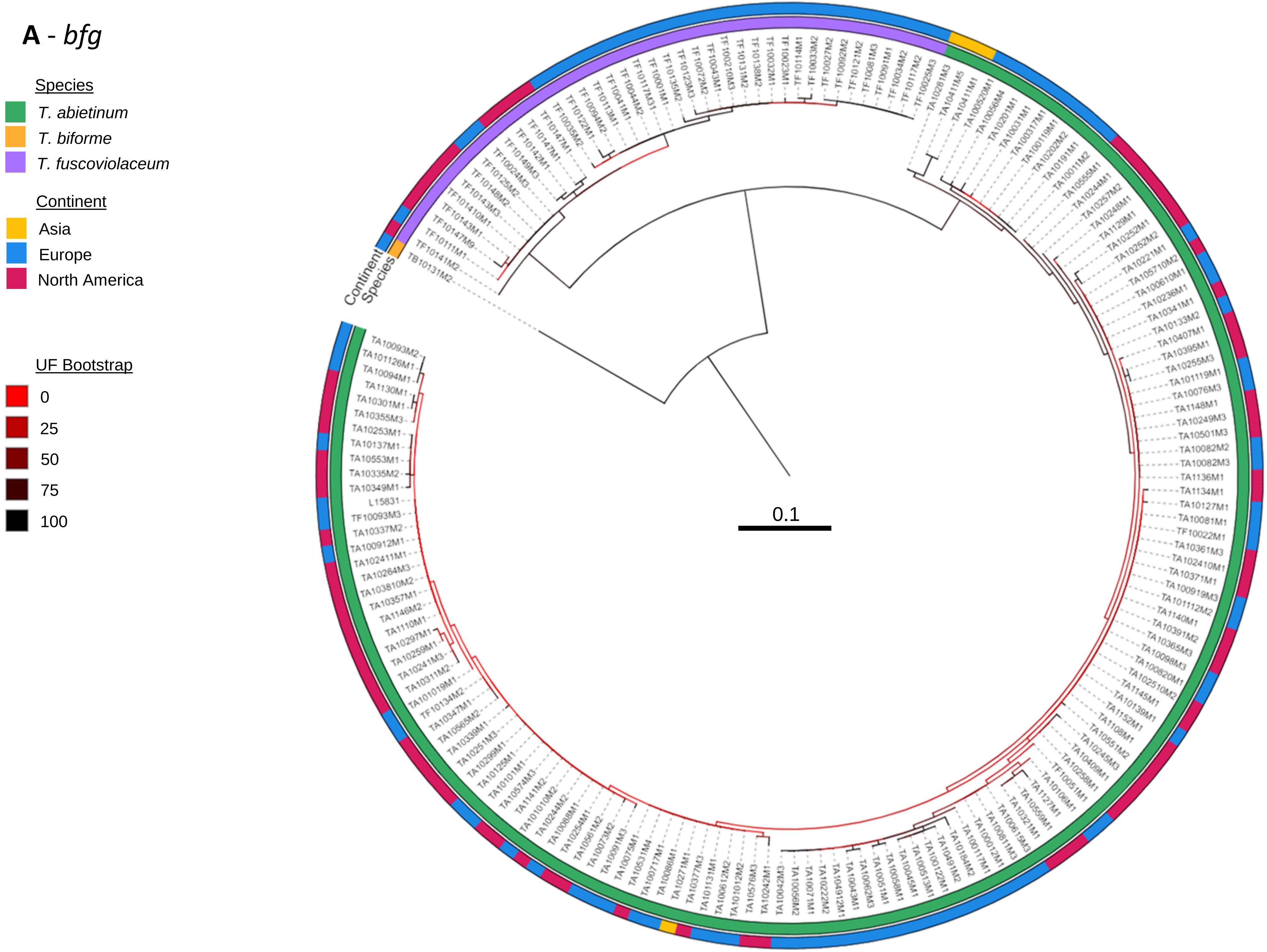
A - bfg
Species
T. abietinum
T. biforme
T. fuscoviolaceum
Continent
Asia
Europe
North America
UF Bootstrap
0
25
50
75
100
Continent
Species
0.1

**B** - *GLGEN*

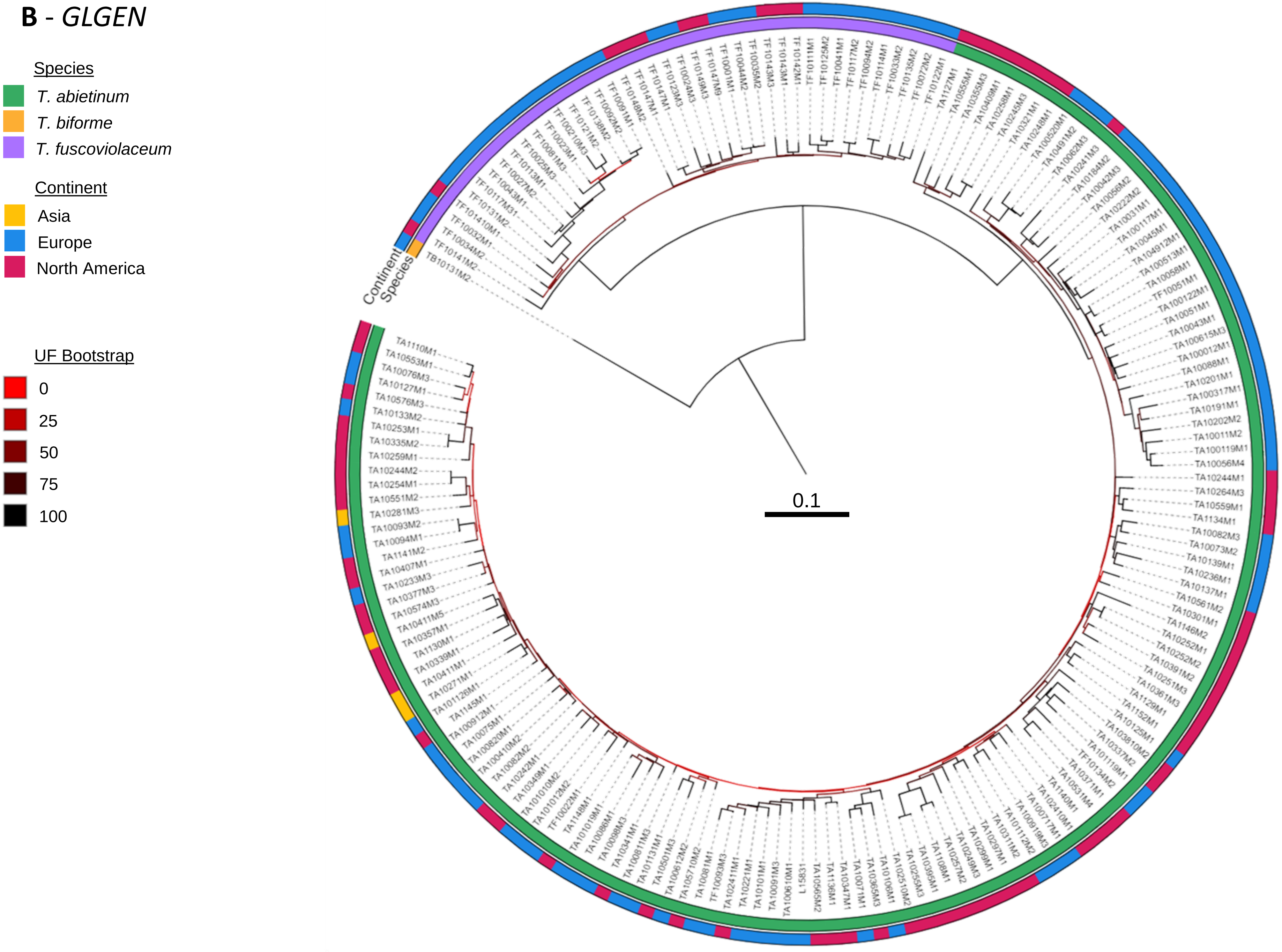
Species
T. abietinum
T. biforme
T. fuscoviolaceum
Continent
Asia
Europe
North America
UF Bootstrap
0
25
50
75
100
Continent
Species
0.1

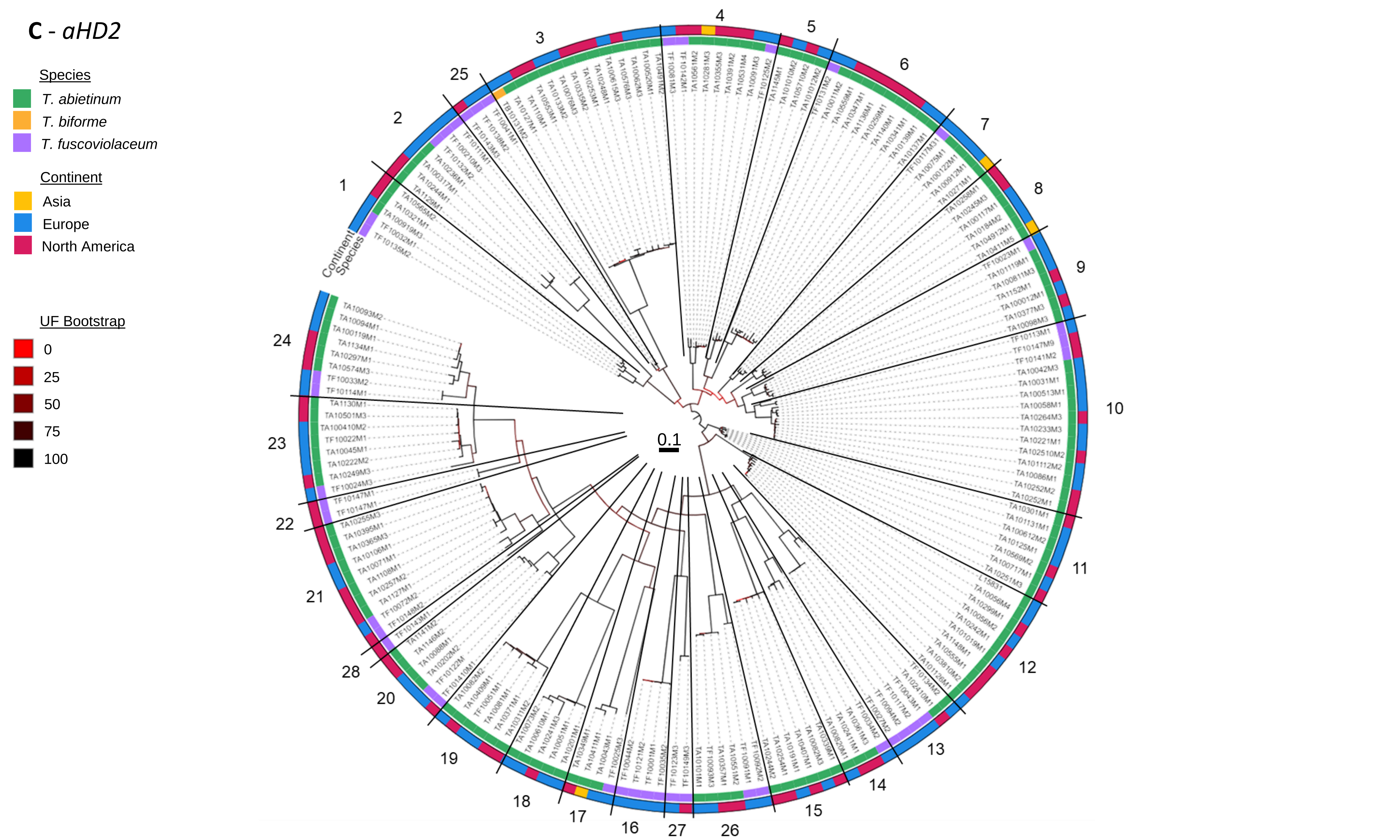

C - aHD2
Species
T. abietinum
T. biforme
T. fuscoviolaceum
Continent
Asia
Europe
North America
UF Bootstrap
0
25
50
75
100
Continent
Species
0.1
1
2
3
4
5
6
7
8
9
10
11
12
13
14
15
16
17
18
19
20
21
22
23
24
25
26
27
28

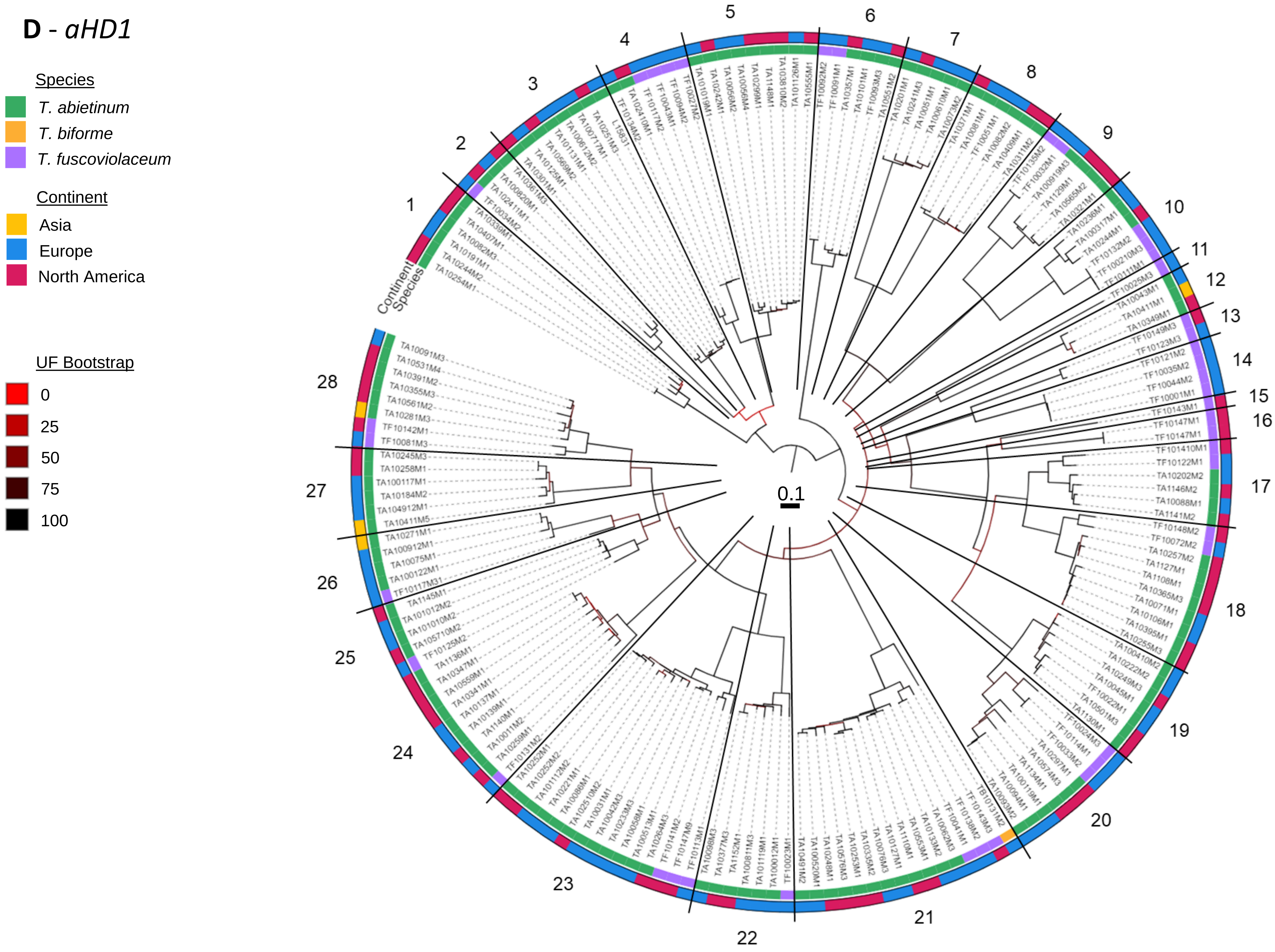
D - aHD1
Species
T. abietinum
T. biforme
T. fuscoviolaceum
Continent
Asia
Europe
North America
UF Bootstrap
0
25
50
75
100
Continent
Species
0.1
1
2
3
4
5
6
7
8
9
10
11
12
13
14
15
16
17
18
19
20
21
22
23
24
25
26
27
28

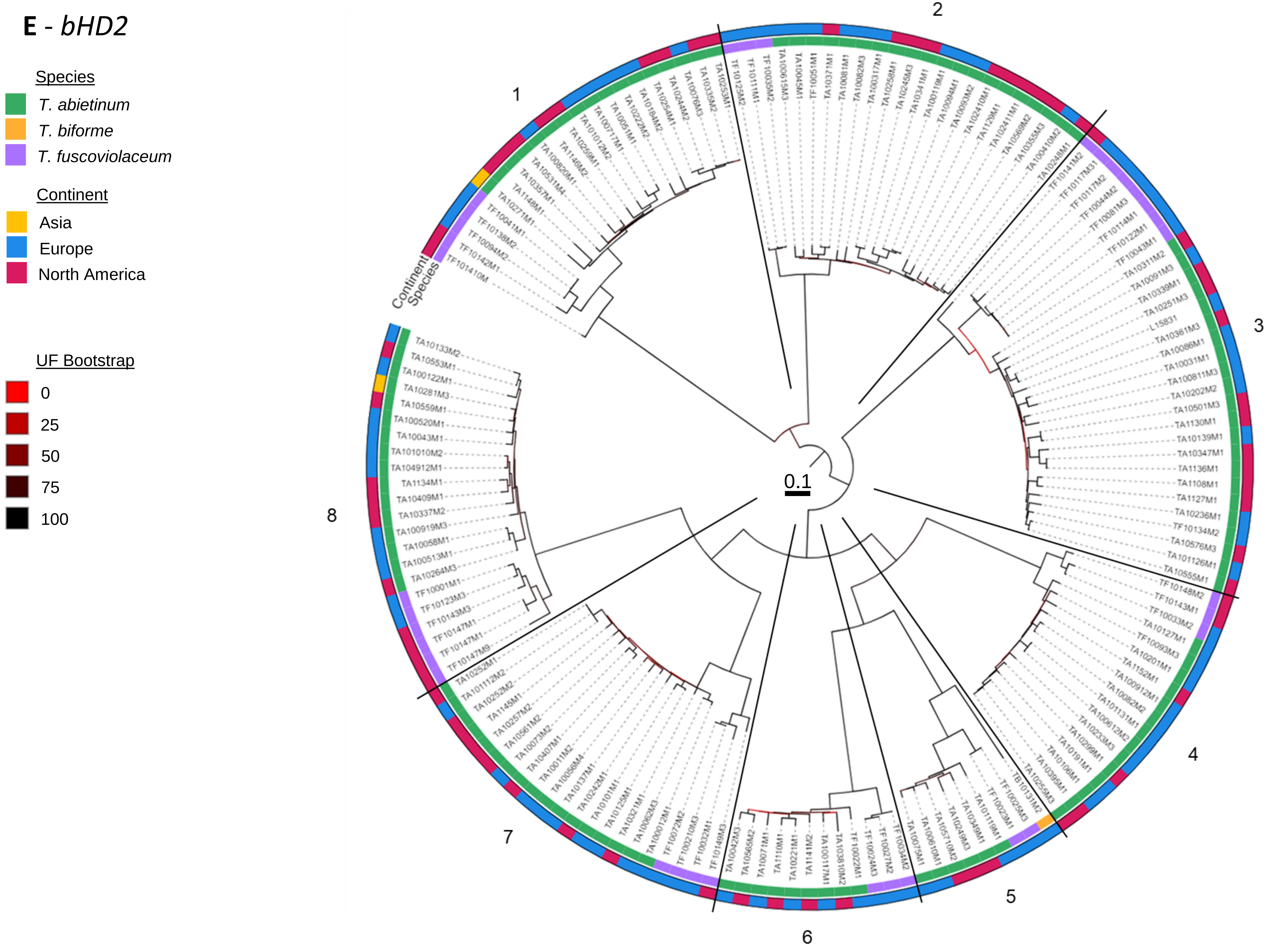
E - bHD2
Species
T. abietinum
T. biforme
T. fuscoviolaceum
Continent
Asia
Europe
North America
UF Bootstrap
0
25
50
75
100
Continent
Species
0.1
1
2
3
4
5
6
7
8

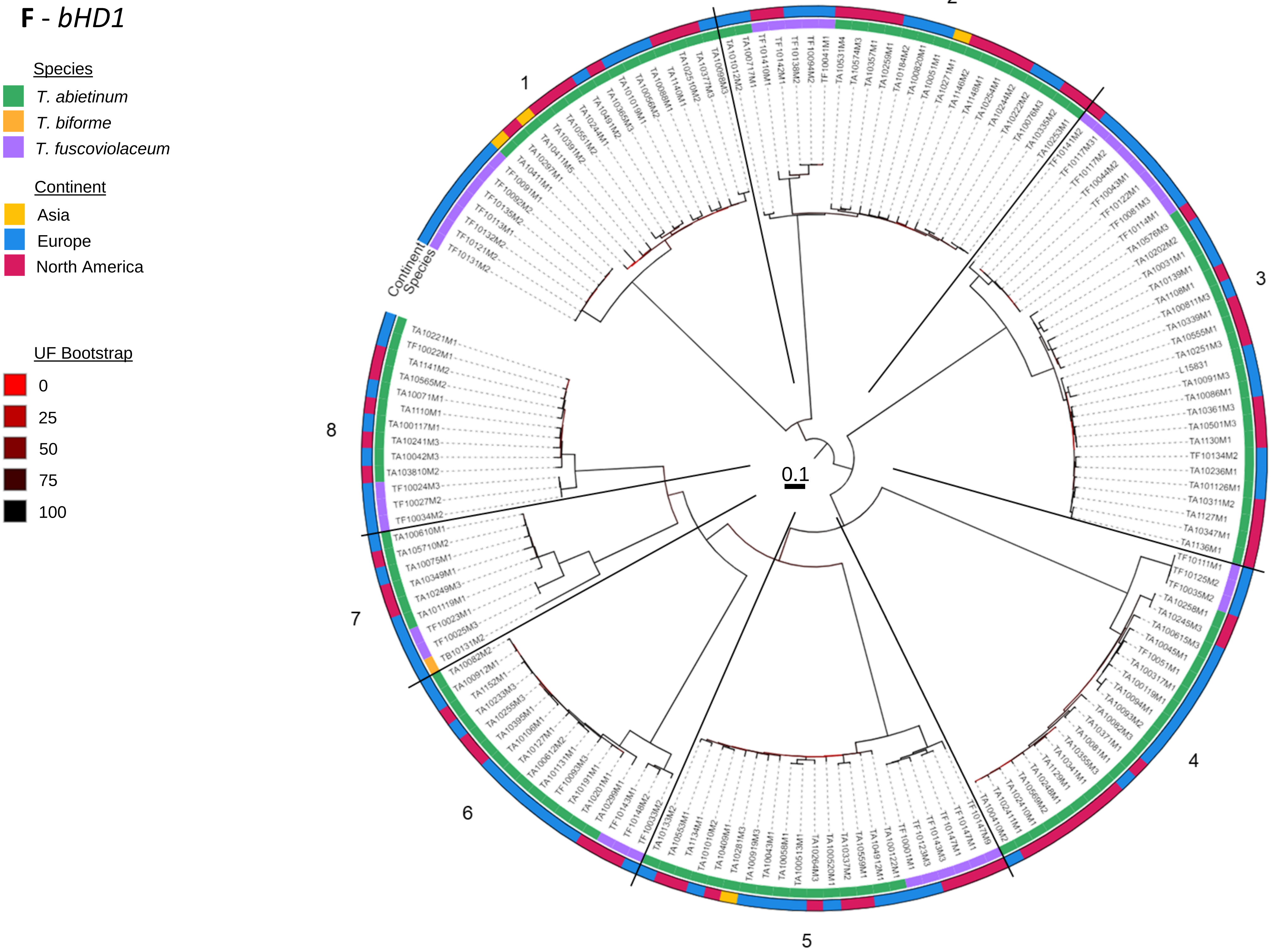

F - bHD1
Species
T. abietinum
T. biforme
T. fuscoviolaceum
Continent
Asia
Europe
North America
UF Bootstrap
0
25
50
75
100
Continent
Species
0.1
1
2
3
4
5
6
7
8

G - *MIP1*

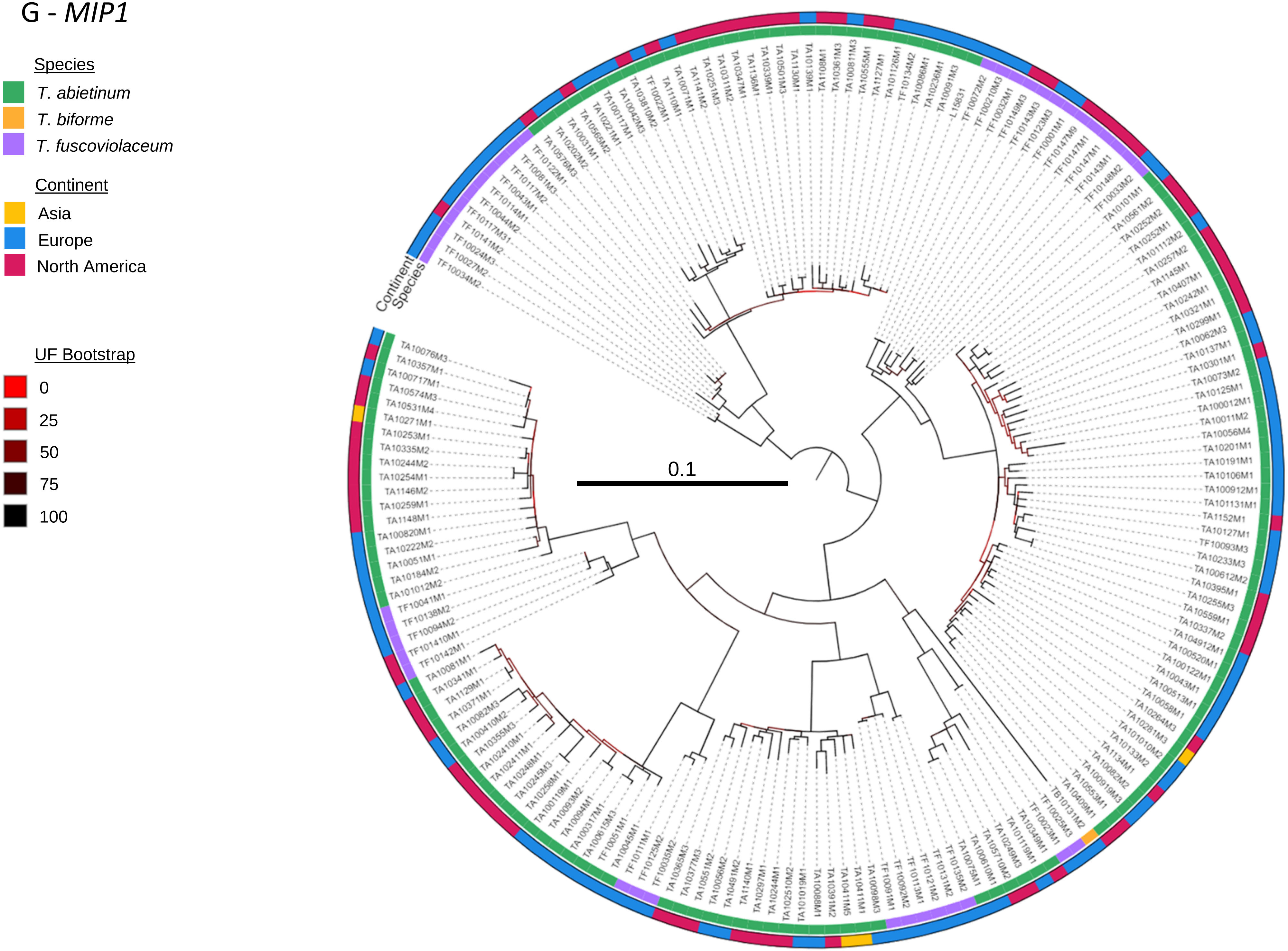
Species
T. abietinum
T. biforme
T. fuscoviolaceum
Continent
Asia
Europe
North America
UF Bootstrap
0
25
50
75
100
0.1
Continent
Species

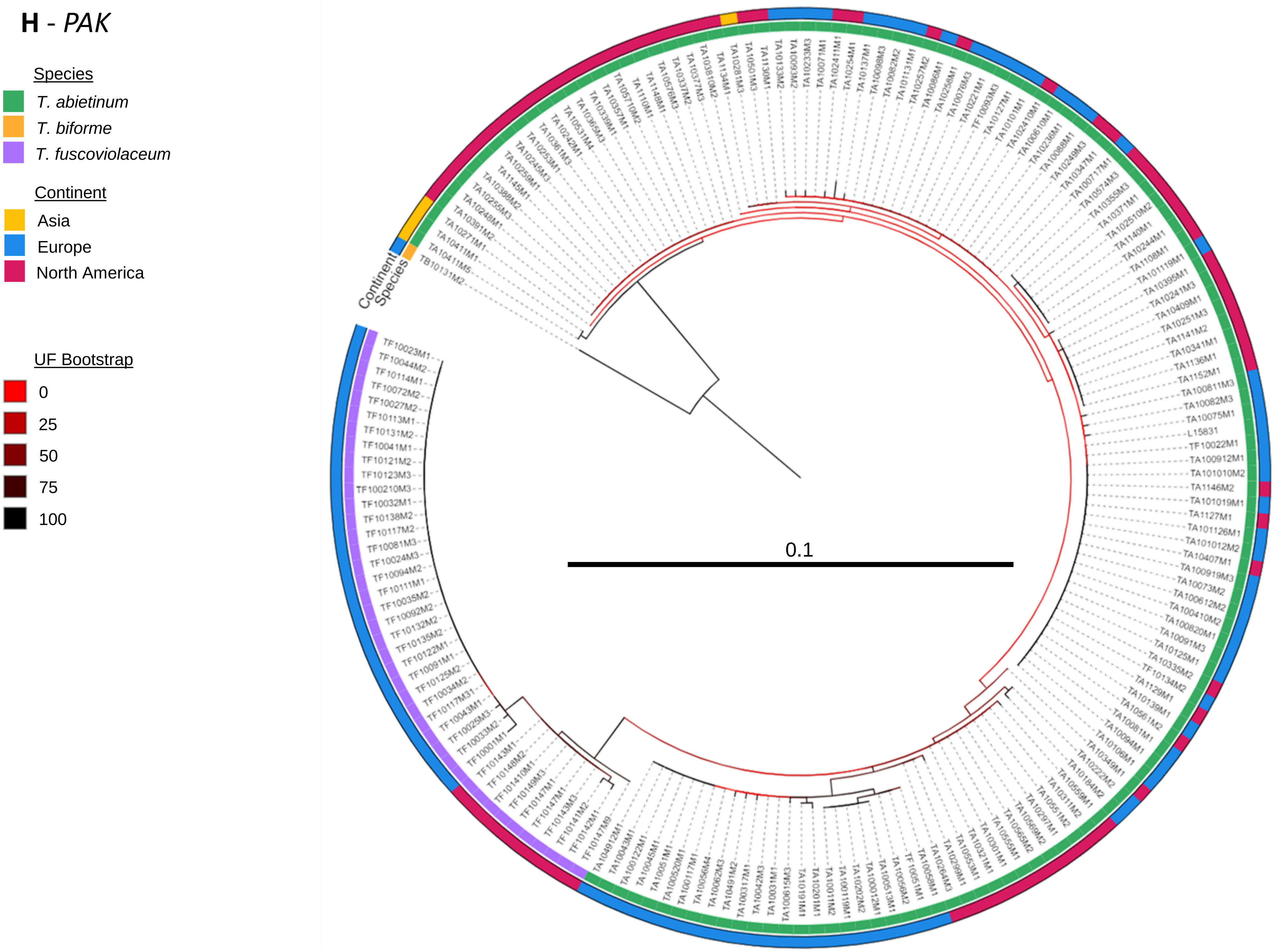
H - PAK
Species
T. abietinum
T. biforme
T. fuscoviolaceum
Continent
Asia
Europe
North America
UF Bootstrap
0
25
50
75
100
Continent
Species
0.1

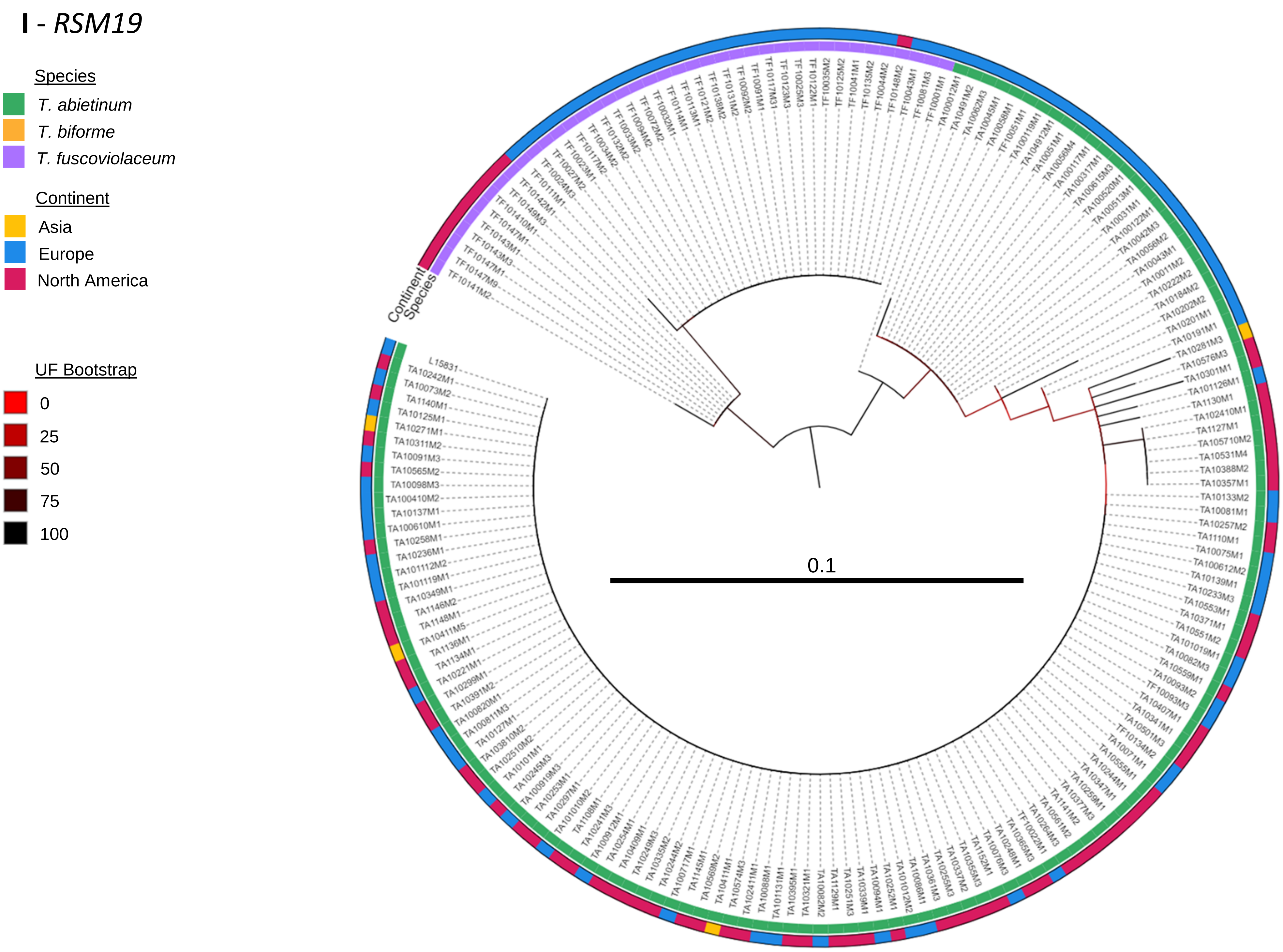

I - RSM19
Species
T. abietinum
T. biforme
T. fuscoviolaceum
Continent
Asia
Europe
North America
UF Bootstrap
0
25
50
75
100
Continent
Species
0.1

**J** - *DML1*

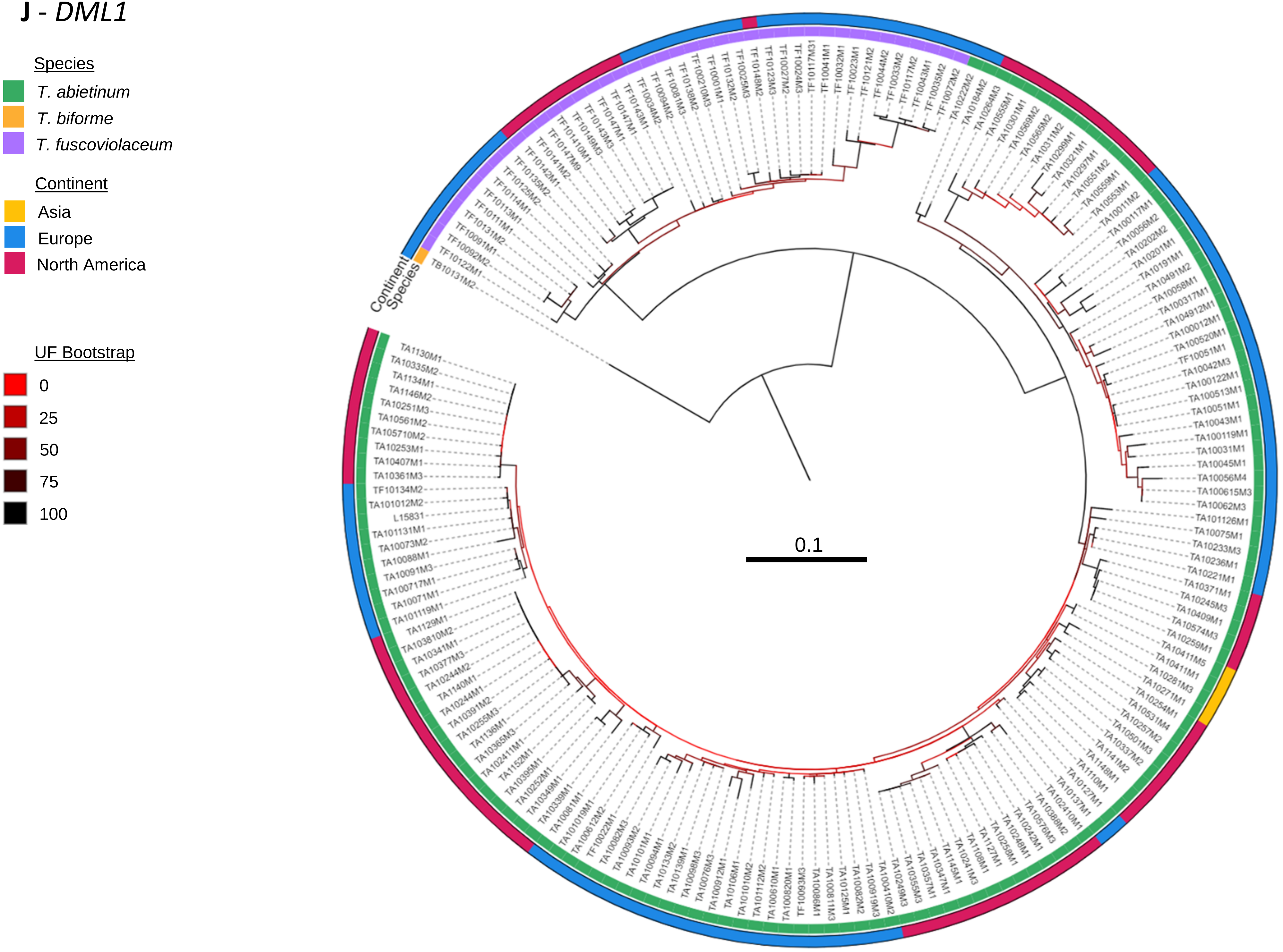

Species
T. abietinum
T. biforme
T. fuscoviolaceum
Continent
Asia
Europe
North America
UF Bootstrap
0
25
50
75
100
Continent
Species
0.1

**K** - *RIC1*

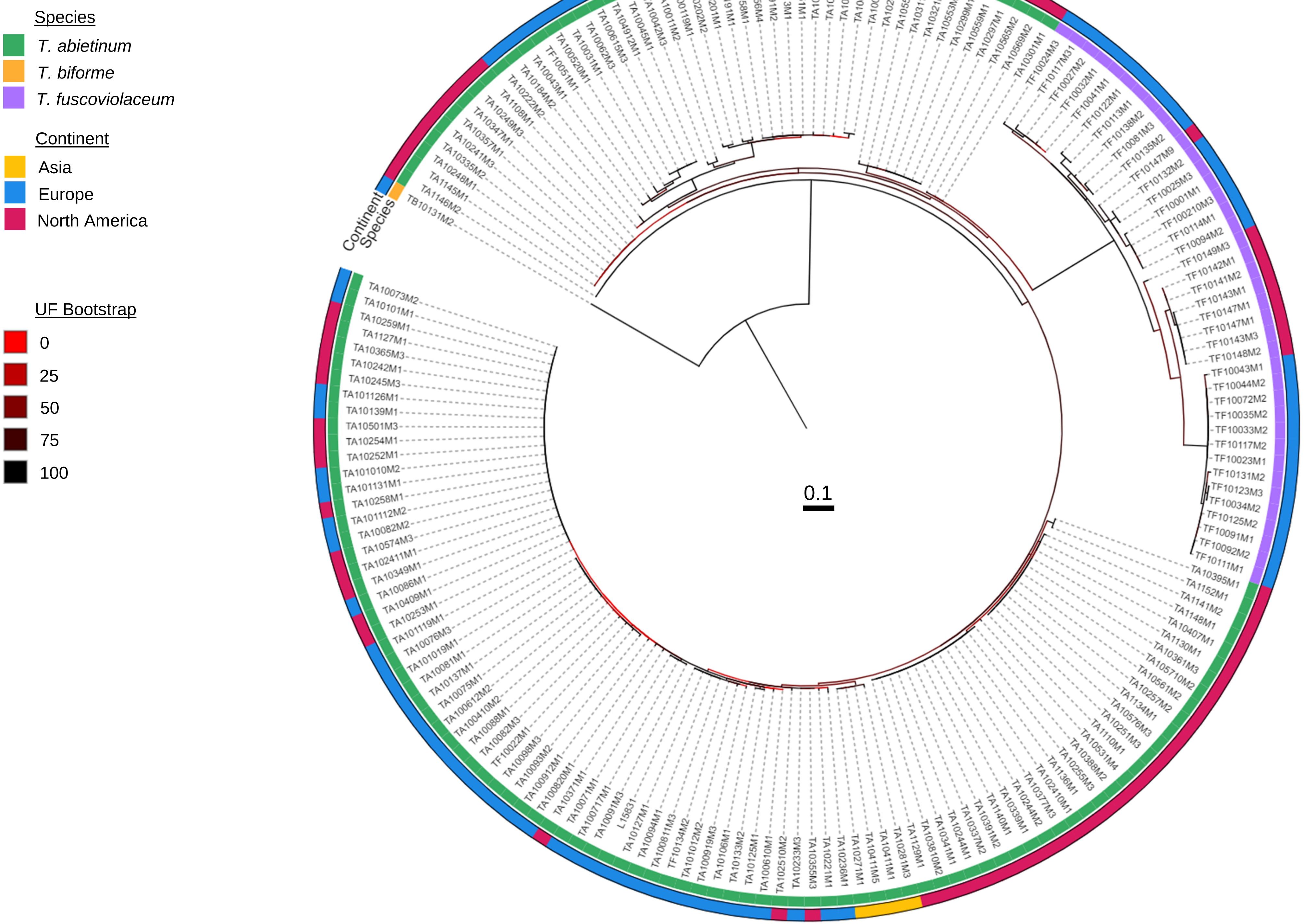
Species
T. abietinum
T. biforme
T. fuscoviolaceum
Continent
Asia
Europe
North America
UF Bootstrap
0
25
50
75
100
Continent
Species
0.1

**L** - *STE3.1*

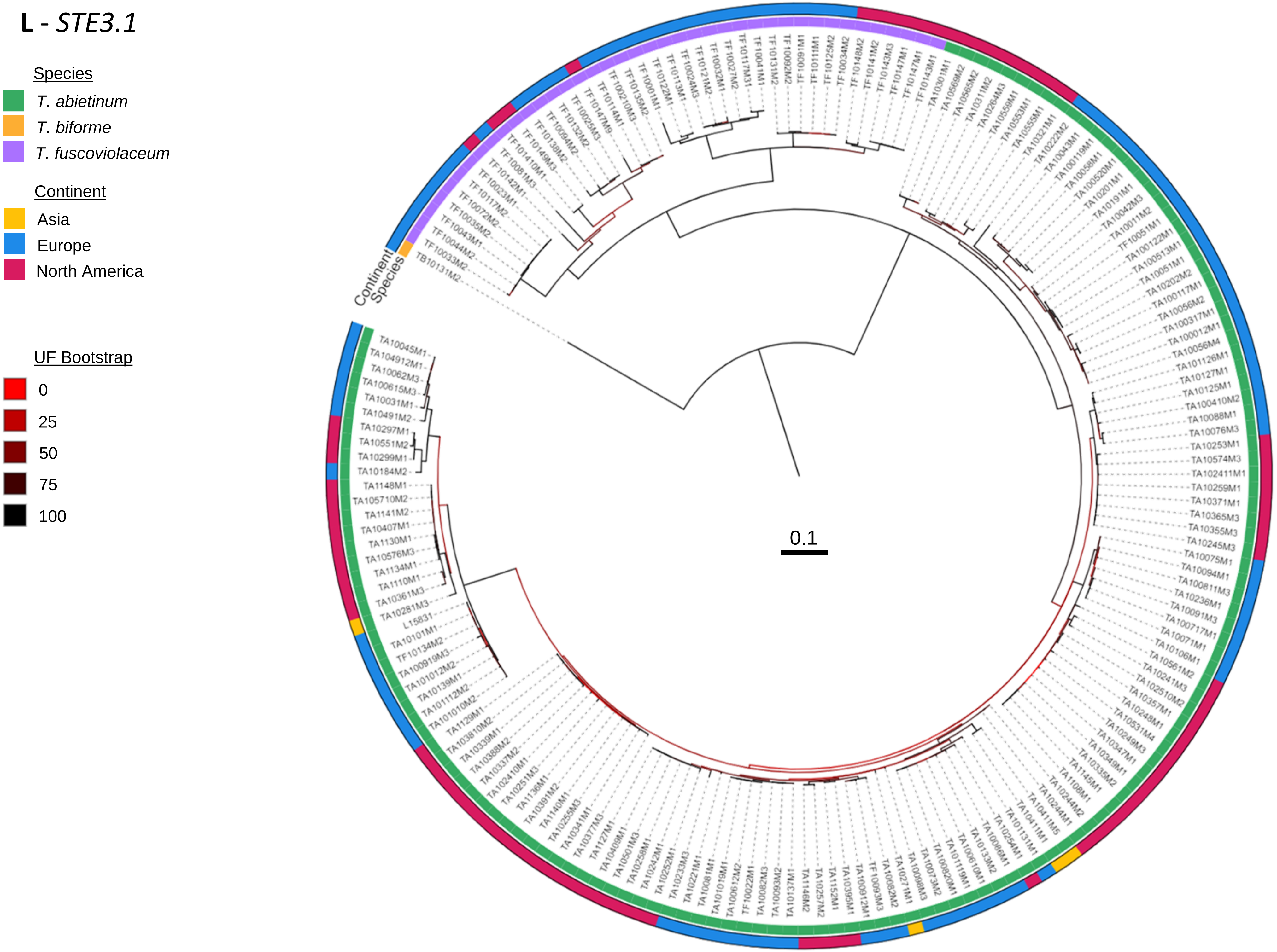
Species
T. abietinum
T. biforme
T. fuscoviolaceum
Continent
Asia
Europe
North America
UF Bootstrap
0
25
50
75
100
Continent
Species
0.1

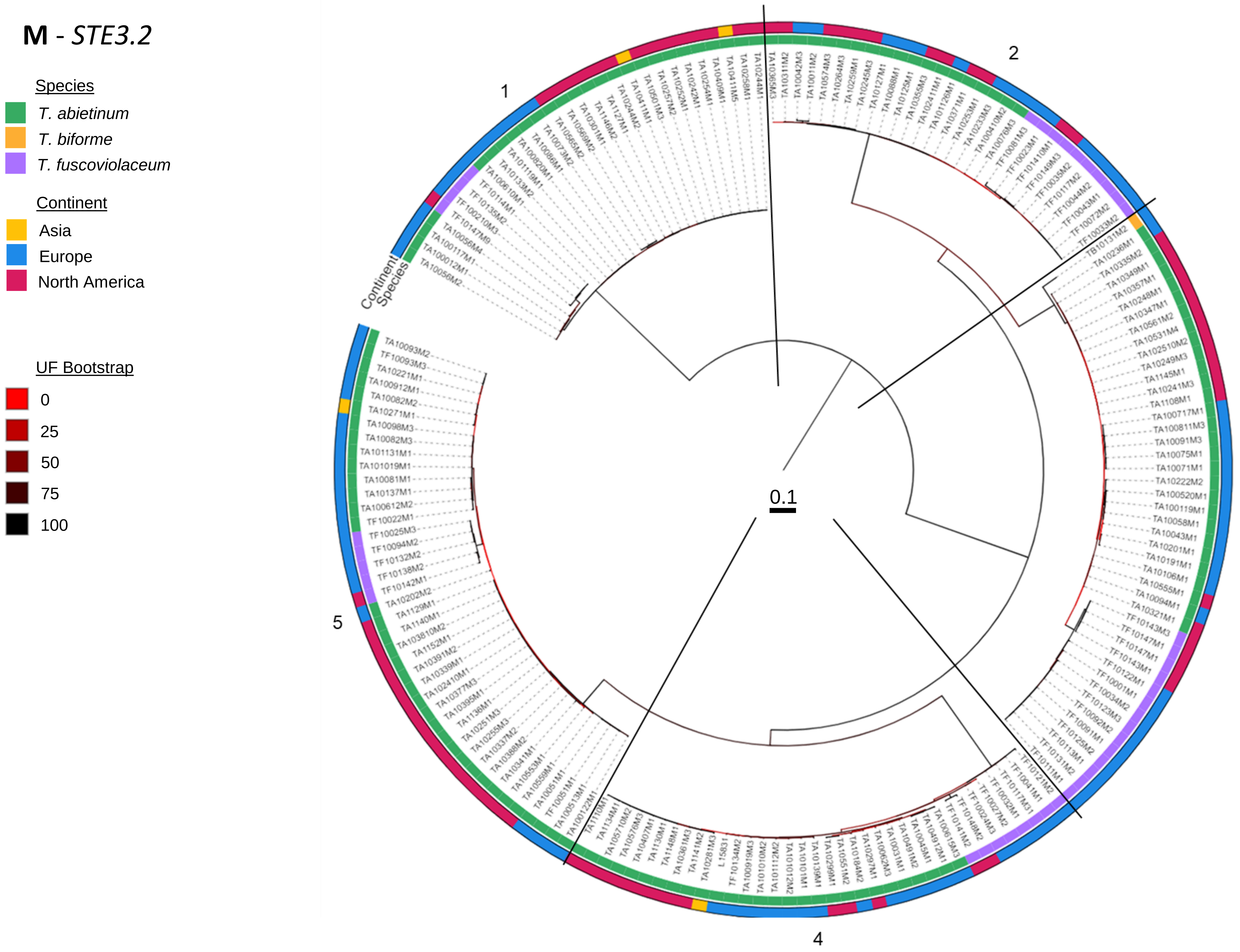
M - STE3.2
Species
T. abietinum
T. biforme
T. fuscoviolaceum
Continent
Asia
Europe
North America
UF Bootstrap
0
25
50
75
100
Continent
Species
0.1
1
2
3
4
5

**N** - *STE3.3*

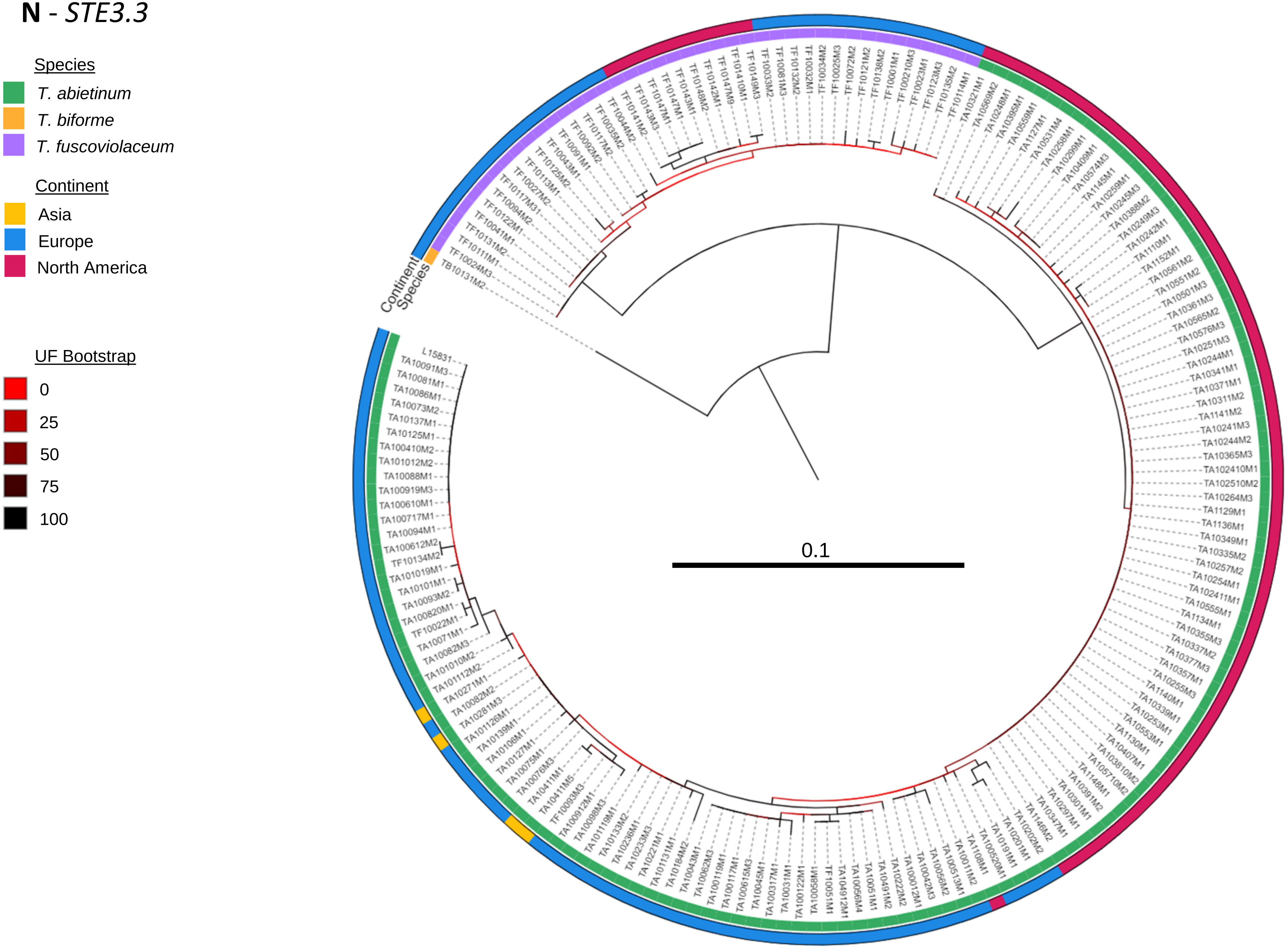
Species
T. abietinum
T. biforme
T. fuscoviolaceum
Continent
Asia
Europe
North America
UF Bootstrap
0
25
50
75
100
Continent
Species
0.1

**O** - *STE3.4*

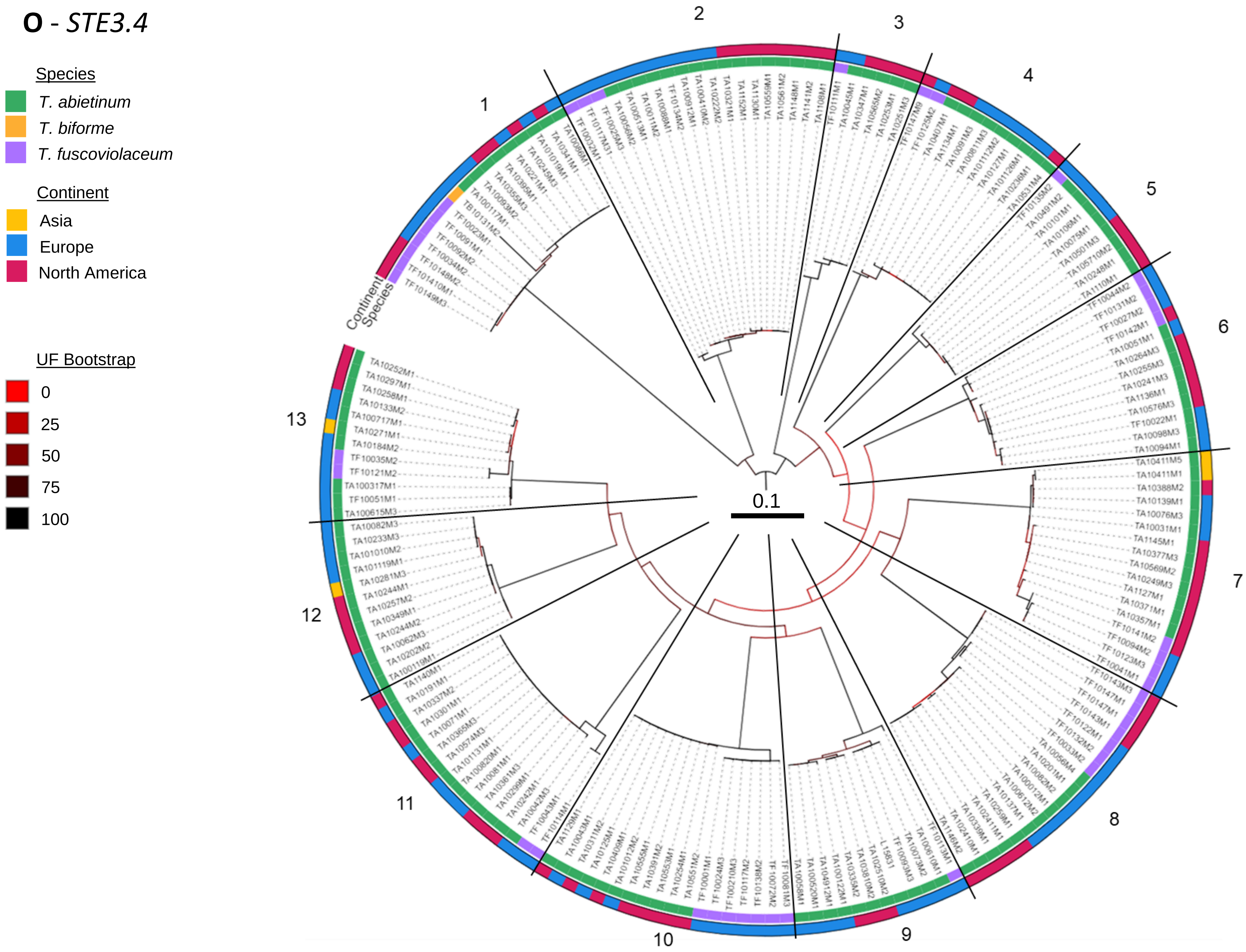
Species
T. abietinum
T. biforme
T. fuscoviolaceum
Continent
Asia
Europe
North America
UF Bootstrap
0
25
50
75
100
Continent
Species
0.1
1
2
3
4
5
6
7
8
9
10
11
12
13

**P** - *SNF2*

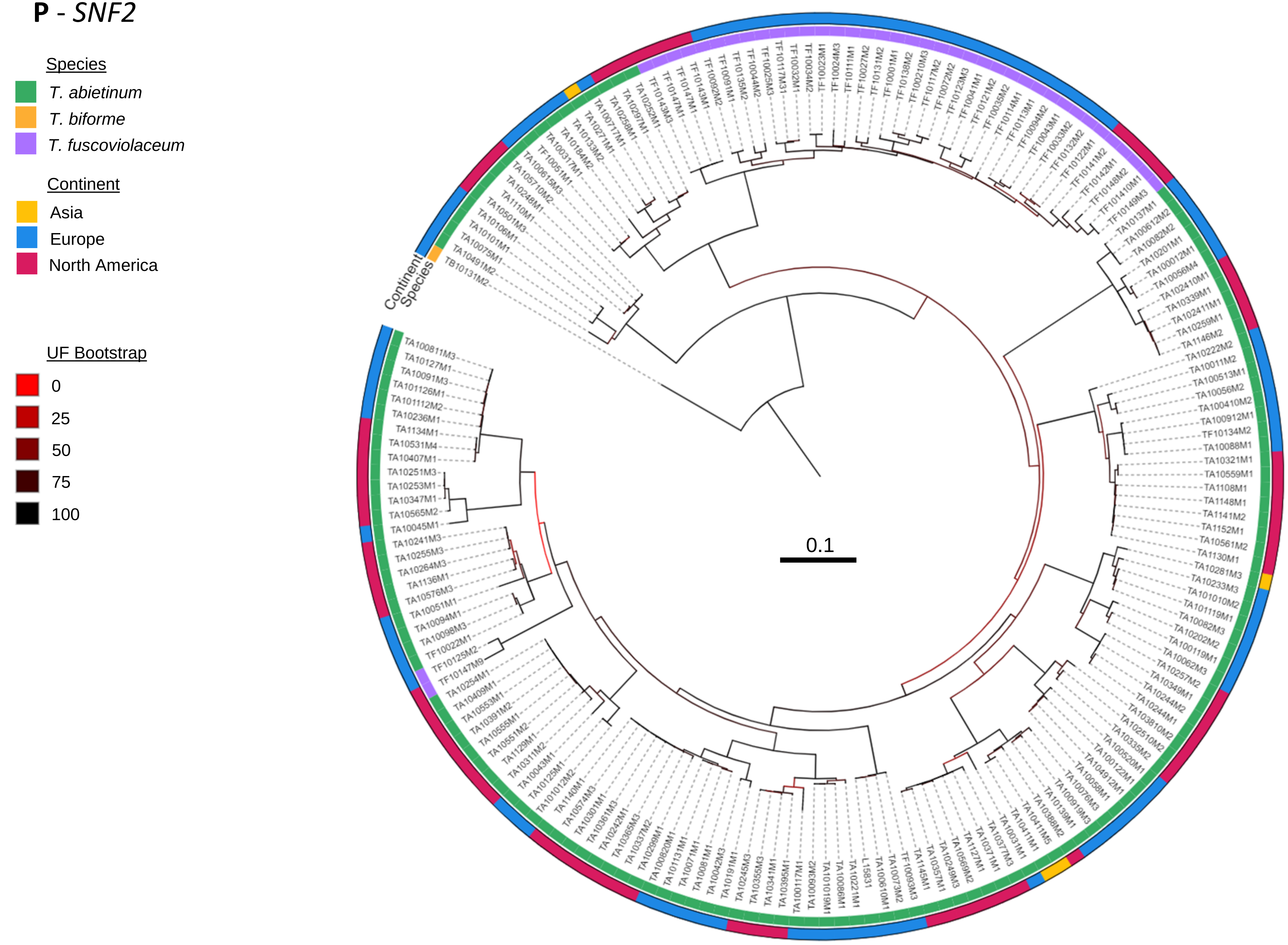
Species
T. abietinum
T. biforme
T. fuscoviolaceum
Continent
Asia
Europe
North America
UF Bootstrap
0
25
50
75
100
Continent
Species
0.1
