# Supplementary material for: Large-scale fungal strain sequencing unravels the molecular diversity in mating loci maintained by long-term balancing selection
Source: PLoS Genet. 2022 Mar 31;18(3):e1010097. doi: 10.1371/journal.pgen.1010097 (PMC8970355; doi:10.1371/journal.pgen.1010097)
Supplement: S8 Fig — Example plate and microscope pictures of the strain cross experiments are displayed on the left and on the right, respectively. Codes on the right, such as TFx1, indicate the type of cross (S5 Table). Pictures of additional crosses are indicated in S5 Table and they can be found in https://perisd.github.io/TriMAT/. When types were distinct in both mating loci clamp connections (red arrows) are observed in septae. Strain names and the inferred allelic classes for each mating gene (S2 Table) are displayed. Compatible MATA complexes or pheromone receptors are highlighted in green in each strain. Tabi, Trichaptum abietinum; Tfus, Trichaptum fuscoviolaceum. (PDF) [file pgen.1010097.s008.pdf]

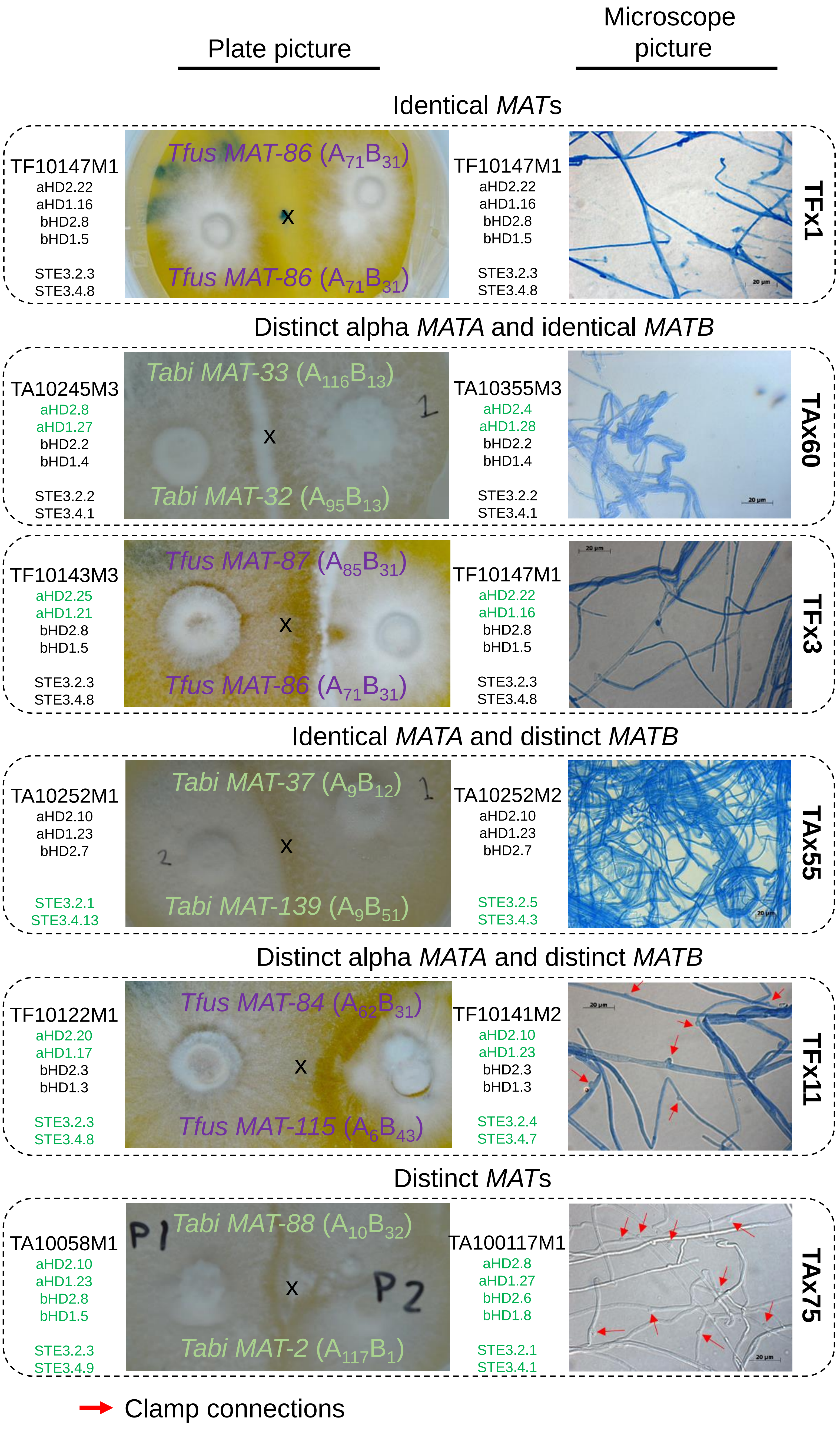

Plate picture
Microscope picture
Identical *MAT*s
TF10147M1
aHD2.22
aHD1.16
bHD2.8
bHD1.5
STE3.2.3
STE3.4.8
Tfus MAT-86 ($A_{71}B_{31}$)
x
Tfus MAT-86 ($A_{71}B_{31}$)
TF10147M1
aHD2.22
aHD1.16
bHD2.8
bHD1.5
STE3.2.3
STE3.4.8
20 µm
TFx1
Distinct alpha *MATA* and identical *MATB*
TA10245M3
aHD2.8
aHD1.27
bHD2.2
bHD1.4
STE3.2.2
STE3.4.1
Tabi MAT-33 ($A_{116}B_{13}$)
1
x
Tabi MAT-32 ($A_{95}B_{13}$)
TA10355M3
aHD2.4
aHD1.28
bHD2.2
bHD1.4
STE3.2.2
STE3.4.1
20 µm
TAx60
TF10143M3
aHD2.25
aHD1.21
bHD2.8
bHD1.5
STE3.2.3
STE3.4.8
Tfus MAT-87 ($A_{85}B_{31}$)
x
Tfus MAT-86 ($A_{71}B_{31}$)
TF10147M1
aHD2.22
aHD1.16
bHD2.8
bHD1.5
STE3.2.3
STE3.4.8
20 µm
TFx3
Identical *MATA* and distinct *MATB*
TA10252M1
aHD2.10
aHD1.23
bHD2.7
STE3.2.1
STE3.4.13
Tabi MAT-37 ($A_{9}B_{12}$)
1
2
x
Tabi MAT-139 ($A_{9}B_{51}$)
TA10252M2
aHD2.10
aHD1.23
bHD2.7
STE3.2.5
STE3.4.3
20 µm
TAx55
Distinct alpha *MATA* and distinct *MATB*
TF10122M1
aHD2.20
aHD1.17
bHD2.3
bHD1.3
STE3.2.3
STE3.4.8
Tfus MAT-84 ($A_{62}B_{31}$)
x
Tfus MAT-115 ($A_{6}B_{43}$)
TF10141M2
aHD2.10
aHD1.23
bHD2.3
bHD1.3
STE3.2.4
STE3.4.7
20 µm
TFx11
Distinct *MAT*s
TA10058M1
aHD2.10
aHD1.23
bHD2.8
bHD1.5
STE3.2.3
STE3.4.9
P1
Tabi MAT-88 ($A_{10}B_{32}$)
x
P2
Tabi MAT-2 ($A_{117}B_{1}$)
TA100117M1
aHD2.8
aHD1.27
bHD2.6
bHD1.8
STE3.2.1
STE3.4.1
20 µm
TAx75
Clamp connections
