# Supplementary material for: Large-scale fungal strain sequencing unravels the molecular diversity in mating loci maintained by long-term balancing selection
Source: PLoS Genet. 2022 Mar 31;18(3):e1010097. doi: 10.1371/journal.pgen.1010097 (PMC8970355; doi:10.1371/journal.pgen.1010097)
Supplement: S13 Fig — Selected regions of ML phylogenetic trees of trimmed (trimal–gt 0.8) protein sequence alignments containing HD2-HD1 and STE3 are displayed in panels A) and B), respectively. Branch support was assessed using the ultrafast bootstrap (UF bootstrap) method. UF bootstrap is indicated in each branch by a gradient color according to the legend. Scale bar is represented in number of amino acid substitutions per site. Trichaptum proteins are highlighted by red arrows or enclosed in a red bar. Allelic classes are indicated in the protein name (i.e. aHDX.Y, where Y is the allelic class). Protein sequences were retrieved from DOE-JGI MycoCosm and download from NCBI as indicated: 1. Hymneochaetales JGI protein list: Fomme: Fomitiporia mediterranea (MF3/22), Onnsc: Onnia scaura (P-53A), Phefer: Phellinidium ferrugineofuscum (SpK3Phefer14), Pheign: Phellinus ignarius (CCBS575), Phevit: Phellinus viticola (PhevitSig-SM15), Pheni: Phellopilus (Phellinus) nigrolimitatus (SigPhenig9), Porchr: Porodaedalea chrysoloma (FP-135951), Pornie: Porodaedalea niemelaei (PN71-100-IP13), Resbic: Resinicium bicolor (OMC78), Ricfib: Rickenella fibula (HBK330-10), Ricmel: Rickenella mellea (SZMC22713), Schpa: Schizopora paradoxa (KUC8140), Sidvul: Sidera vulgaris (OMC1730). 2. Downloaded from NCBI: [HYMENOCHAETALES] Fomitiporia mediterranea (MF3/22), Pyrrhoderma noxium (KPN91), Shanghuangporus baumii (Bpt 821), Rickenella mellea (SZMC22713); [AGARICALES] Laccaria bicolor (S238N-H82), Coprinopsis cinerea (Okayama7#130); [POLYPORALES] Rhodonia (Postia) placenta (Mad-698-R). To remove protein redundancy in protein collection of species retrieved from JGI, a blastp using the downloaded NCBI protein sequences and HDs and STE3s protein representatives of each allelic class was performed. For each input sequence two hits were used for sequence alignments, a protein sequence with the lowest e-value and the protein sequence with the highest coverage value. Complete ML phylogenetic trees are deposited in a s [file pgen.1010097.s013.pdf]

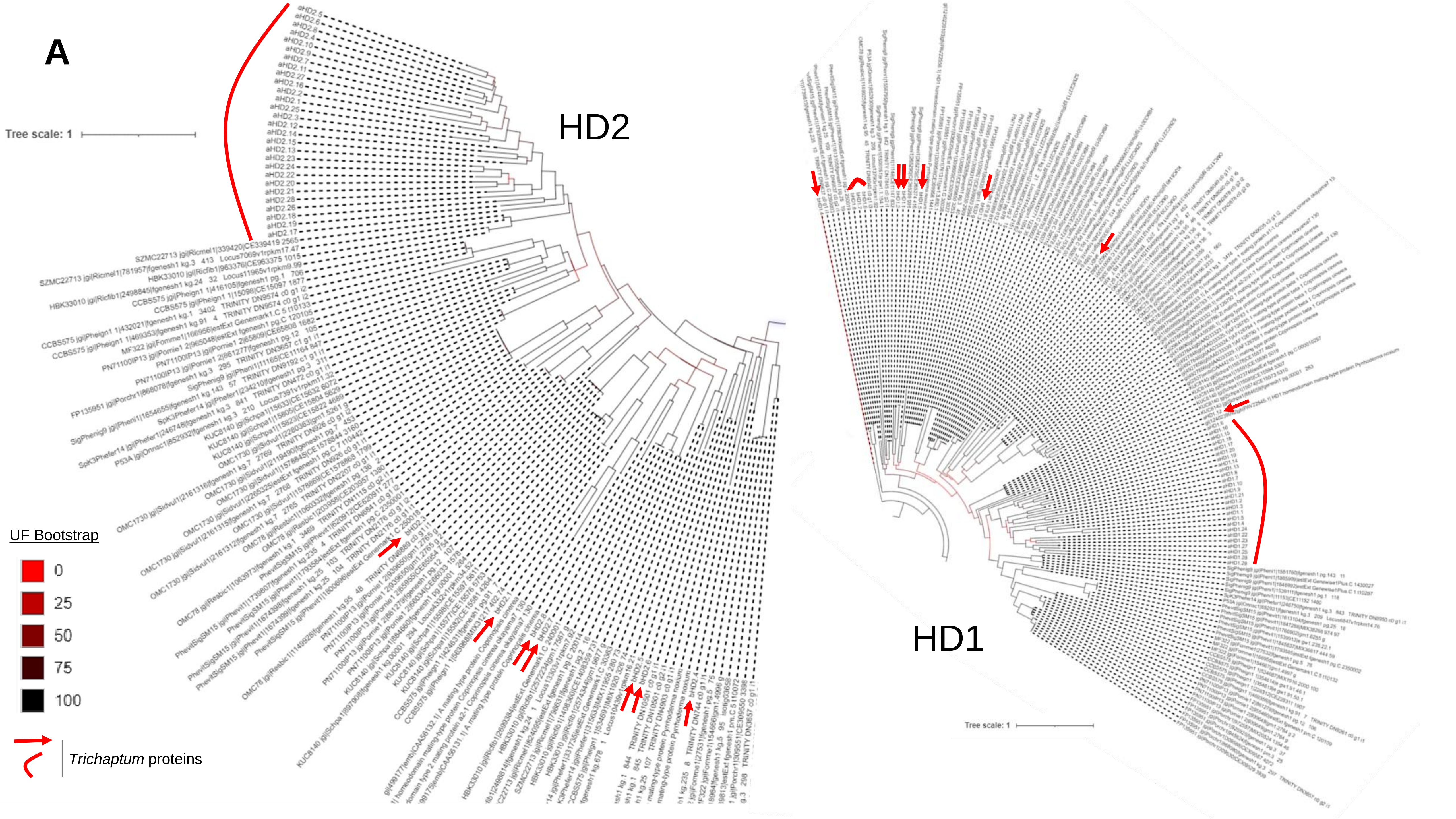
A
HD2
HD1
Tree scale: 1
Tree scale: 1
UF Bootstrap
0
25
50
75
100
Trichaptum proteins

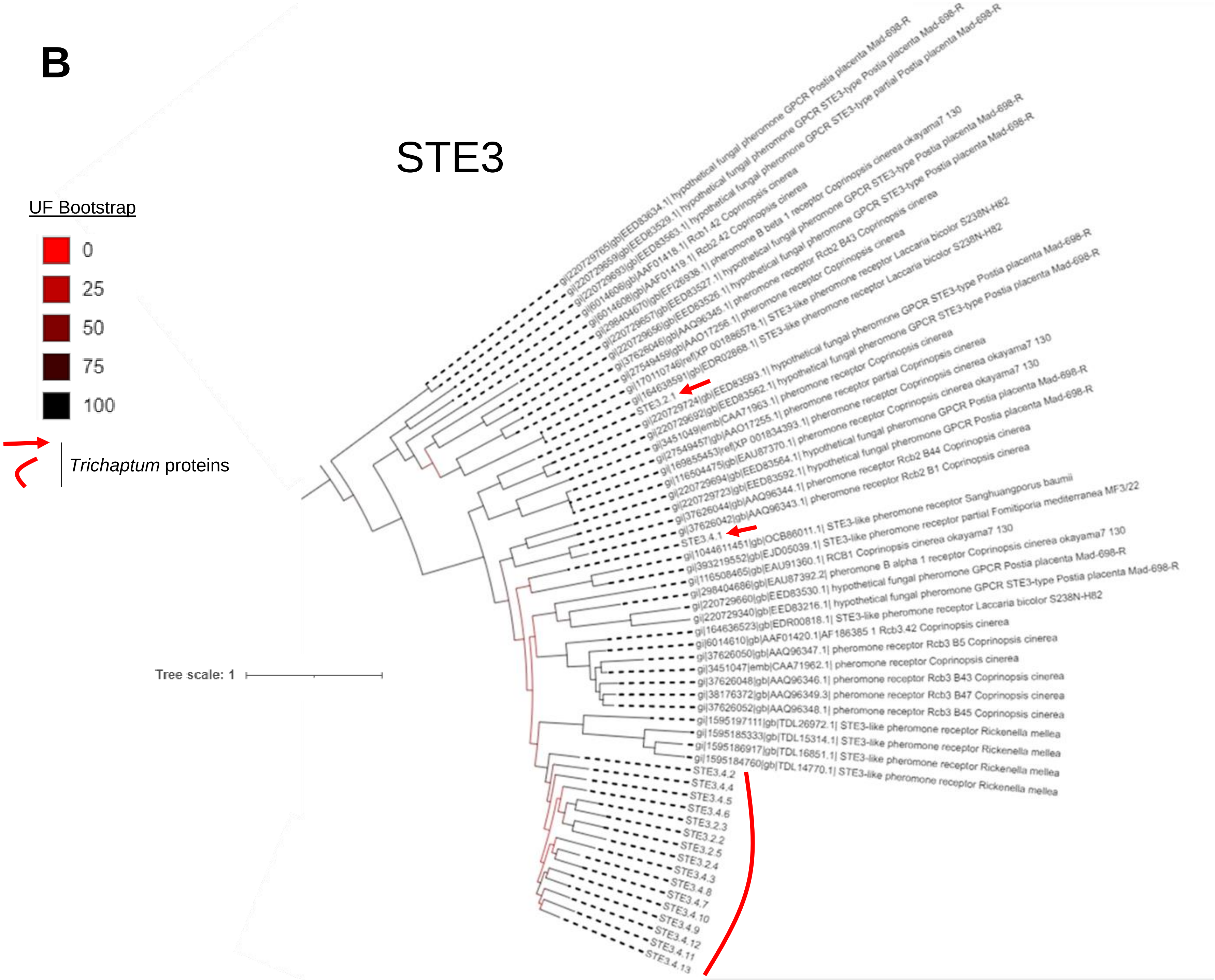
B
STE3
UF Bootstrap
0
25
50
75
100
Trichaptum proteins
Tree scale: 1
gi|220729765|gb|EED83634.1| hypothetical fungal pheromone GPCR Postia placenta Mad-698-R
gi|220729659|gb|EED83529.1| hypothetical fungal pheromone GPCR STE3-type Postia placenta Mad-698-R
gi|220729693|gb|EED83563.1| hypothetical fungal pheromone GPCR STE3-type partial Postia placenta Mad-698-R
gi|6014606|gb|AAF01418.1| Rcb1.42 Coprinopsis cinerea
gi|6014608|gb|AAF01419.1| Rcb2.42 Coprinopsis cinerea
gi|298404670|gb|EFI26938.1| pheromone B beta 1 receptor Coprinopsis cinerea okayama7 130
gi|220729657|gb|EED83527.1| hypothetical fungal pheromone GPCR STE3-type Postia placenta Mad-698-R
gi|220729656|gb|EED83526.1| hypothetical fungal pheromone GPCR STE3-type Postia placenta Mad-698-R
gi|37626046|gb|AAQ96345.1| pheromone receptor Rcb2 B43 Coprinopsis cinerea
gi|27549459|gb|AAO17256.1| pheromone receptor Coprinopsis cinerea
gi|170110746|ref|XP 001886578.1| STE3-like pheromone receptor Laccaria bicolor S238N-H82
gi|164638591|gb|EDR02868.1| STE3-like pheromone receptor Laccaria bicolor S238N-H82
STE3.2.1
gi|220729724|gb|EED83593.1| hypothetical fungal pheromone GPCR STE3-type Postia placenta Mad-698-R
gi|220729692|gb|EED83562.1| hypothetical fungal pheromone GPCR STE3-type Postia placenta Mad-698-R
gi|3451049|emb|CAA71963.1| pheromone receptor Coprinopsis cinerea
gi|27549457|gb|AAO17255.1| pheromone receptor partial Coprinopsis cinerea
gi|169855453|ref|XP 001834393.1| pheromone receptor Coprinopsis cinerea okayama7 130
gi|116504475|gb|EAU87370.1| pheromone receptor Coprinopsis cinerea okayama7 130
gi|220729694|gb|EED83564.1| hypothetical fungal pheromone GPCR Postia placenta Mad-698-R
gi|220729723|gb|EED83592.1| hypothetical fungal pheromone GPCR Postia placenta Mad-698-R
gi|37626044|gb|AAQ96344.1| pheromone receptor Rcb2 B44 Coprinopsis cinerea
gi|37626042|gb|AAQ96343.1| pheromone receptor Rcb2 B1 Coprinopsis cinerea
STE3.4.1
gi|1044611451|gb|OCB86011.1| STE3-like pheromone receptor Sanghuangporus baumii
gi|393219552|gb|EJD05039.1| STE3-like pheromone receptor partial Fomitiporia mediterranea MF3/22
gi|116508465|gb|EAU91360.1| RCB1 Coprinopsis cinerea okayama7 130
gi|298404686|gb|EAU87392.2| pheromone B alpha 1 receptor Coprinopsis cinerea okayama7 130
gi|220729660|gb|EED83530.1| hypothetical fungal pheromone GPCR Postia placenta Mad-698-R
gi|220729340|gb|EED83216.1| hypothetical fungal pheromone GPCR STE3-type Postia placenta Mad-698-R
gi|164636523|gb|EDR00818.1| STE3-like pheromone receptor Laccaria bicolor S238N-H82
gi|6014610|gb|AAF01420.1|AF186385 1 Rcb3.42 Coprinopsis cinerea
gi|37626050|gb|AAQ96347.1| pheromone receptor Rcb3 B5 Coprinopsis cinerea
gi|3451047|emb|CAA71962.1| pheromone receptor Coprinopsis cinerea
gi|37626048|gb|AAQ96346.1| pheromone receptor Rcb3 B43 Coprinopsis cinerea
gi|38176372|gb|AAQ96349.3| pheromone receptor Rcb3 B47 Coprinopsis cinerea
gi|37626052|gb|AAQ96348.1| pheromone receptor Rcb3 B45 Coprinopsis cinerea
gi|1595197111|gb|TDL26972.1| STE3-like pheromone receptor Rickenella mellea
gi|1595185333|gb|TDL15314.1| STE3-like pheromone receptor Rickenella mellea
gi|1595186917|gb|TDL16851.1| STE3-like pheromone receptor Rickenella mellea
gi|1595184760|gb|TDL14770.1| STE3-like pheromone receptor Rickenella mellea
STE3.4.2
STE3.4.4
STE3.4.5
STE3.4.6
STE3.2.3
STE3.2.2
STE3.2.5
STE3.2.4
STE3.4.3
STE3.4.8
STE3.4.7
STE3.4.10
STE3.4.9
STE3.4.12
STE3.4.11
STE3.4.13
